# Micro‐Petal Cobalt‐Doped FeMoB Framework for Efficient Hydrogen Generation in Ampere‐Level Water Electrolysis

**DOI:** 10.1002/smll.202511038

**Published:** 2025-11-24

**Authors:** Mehedi Hasan Joni, Sumiya Akter Dristy, Md Najibullah, Md Ahasan Habib, Shusen Lin, Jihoon Lee

**Affiliations:** ^1^ Department of Electronic Engineering College of Electronics and Information Kwangwoon University Nowon‐gu Seoul 01897 South Korea

**Keywords:** 3d‐block metal, doping, electrocatalyst, large‐current‐density, stability

## Abstract

Efforts to develop highly efficient, affordable replacements for noble/precious metal electrocatalysts for hydrogen generation via water electrolysis continue to face critical challenges in addressing global energy and environmental concerns. Herein, Co‐doped FeMoB micro‐petal (MP) novel framework is demonstrated via a two‐step hydrothermal approach, followed by thermal annealing treatment. The optimized Co/FeMoB MP exhibits significantly enhanced HER/OER performance, requiring only 54/257 mV at 100 mA cm^−2^ in 1 m KOH, ranking it among the most promising dual‐functional electrocatalysts. For overall water‐splitting, the bifunctional MP (− /̸̸ / +) system delivers an ultra‐low cell voltage of 2.87 V at 2000 mA cm^−2^ and maintains continuous stability for 250 h at 600 mA cm^−2^. In addition, the hybrid Co/FeMoB electrode delivers a record‐low cell voltage of 2.26 V at large‐current‐density in 6 m KOH at 60 °C, demonstrating excellent feasibility for large‐scale hydrogen production under harsh industrial conditions. The superior multifunctional properties of Co‐doped FeMoB are attributed to the electronic/structural modulation by 3d‐block metal effects, elemental synergism, abundant active sites, polycrystallinity, and expanded electrochemically active surface. This study demonstrates that a trace level of the introduced Co into the active bimetallic FeMoB matrix can significantly enhance its electrocatalytic activity toward next‐generation and commercial H_2_O electrolysis.

## Graphical abstract

Micro‐petal (MP) bifunctional system exhibits superior water electrolysis performance, delivering 2.87 V at a large‐current‐density (LCD) of 2000 mA cm^−2^, which is comparable to the benchmark setup. Significantly, the hybrid configuration achieves a record‐low 2‐E cell voltage of 2.26 V at LCD. Further, the bifunctional MP demonstrates remarkable stability, maintaining continuous operation for 250 h under 600 mA cm^−2^ in 1/6 M KOH.

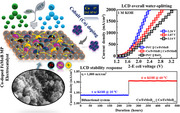

## 1 Introduction

Hydrogen is widely recognized as a highly promising alternative clean energy medium due to its zero‐carbon emission profile, ultrahigh energy density, and excellent recyclability, making it a key pillar in the global energy transition to resolve challenges of energy insecurity, environmental degradation, and long‐term climate threats.^[^ ^1^, ^2^ ^]^ Electrocatalytic water electrolysis (EWE) is a highly efficient method for the cathodic hydrogen evolution reaction (HER, 4H_2_O + 4e^−^ → 4OH^−^ + 2H_2_) and anodic oxygen evolution reaction (OER, 4OH^−^ → 2H_2_O + 4e^−^ + O_2_) for producing high‐purity hydrogen, and accelerating its slow kinetics requires high‐performance electrocatalysts.^[^ ^3^, ^4^ ^]^ Currently, noble metal‐focused catalysts, such as Pt‐ and Ru/Ir‐based materials serve as benchmarks for HER/OER;^[^ ^5^, ^6^ ^]^ however, their high cost and limited availability restrict their practical applications. Therefore, the construction of low‐cost, high‐performance, and stable electrocatalysts capable of operating at large currents is crucial for energy‐efficient H_2_O catalysis and industrial‐scale hydrogen production.

A wide range of advanced research focusing on transition metal boride (TM‐B) materials has been extensively attempted for water electrolysis applications due to their low price, earth abundance, metallic properties, and chemical stability.^[^ ^7^, ^8^, ^9^, ^10^ ^]^ In terms of base TM‐B framework, bimetallic iron (Fe) and molybdenum (Mo) components can regulate electronic structure through d‐band orbital hybridization, thereby optimizing adsorption/desorption of HER/OER intermediates, which can lead to enhanced intrinsic catalytic activity and improved conductivity. The Fe atoms can create super‐exchange pathways in a multi‐metallic crystal lattice through proper atomic arrangements. This may mediate electron transport between cationic centers, thereby improving the OER performance.^[^ ^11^ ^]^ For example, dual‐functional Fe/Ni@NSC nanoparticles exhibited enhanced OER and water‐splitting performance due to their excellent mass transport efficiency and higher specific active surface area.^[^ ^12^ ^]^ Meanwhile, the Mo can offer a high density of active surface electrons through electron acceptance, while its multivalent oxide states accelerate electrocatalytic efficiency and also provide superior mechanical strength with corrosion resistance during H_2_O separation process.^[^ ^13^, ^14^ ^]^ For instance, TM‐based alloy NiMoO_4_@g–CN nanostructure composite exhibited impressive HER/OER activity in multi‐pH systems due to higher morphological stability and robust redox activity by ion diffusion capacity.^[^ ^15^ ^]^ On the other hand, non‐metallic boron (B) can modify the TM matrix and exhibit robust multi‐bonding characteristics, demonstrating high charge transport promotion, catalytic efficiency, and structural durability.^[^ ^16^, ^17^ ^]^ In addition, the presence of B can enhance catalytic turnover through reversible and rapid oxidation‐reduction transitions, facilitated by the exposure of surface‐coordinatively unsaturated active sites.^[^ ^18^ ^]^ For example, the NiB–Zn@HP electrocatalyst exhibited accelerated reaction kinetics and improved cycling stability during large‐current H_2_O electrolysis, which can be attributed to structural modifications and enhanced surface hydrophilicity.^[^ ^19^ ^]^ To enhance catalytic activity and stability, the systematic 3d‐block metal doping (3d‐MD;, e.g., Fe, W, Co, Ni, Cr, etc.) in host material systems holds great potential, offering multiple advantages such as abundant active sites derived from added dopant atoms, optimized electronic structure, and compositional diversity.^[^ ^20^, ^21^, ^22^ ^]^ Among various 3d‐MD elements, the cost‐effective cobalt (Co) can be a highly efficient dopant for modifying the electronic structure and lowering the adsorption energies of H‐ and O‐containing intermediates (H*, O*, OH*, OOH*).^[^ ^23^, ^24^ ^]^ A small amount of low‐cost (≈$15.12/pound) Co doping into the TM‐B alloy structure can enhance catalytic capability by providing additional active sites and enriching electron density.^[^ ^25^, ^26^ ^]^ Besides that, the strong electron affinity, versatile redox functionality, and multiple valence states of cobalt (Co^2+^, Co^3+^, and Co^4+^) can facilitate the HER/OER processes and ultimately accelerate overall water (H–O–H) dissociation efficiency.^[^ ^24^, ^27^ ^]^ Moreover, the Co inclusion can improve the crystallinity and mechanical integrity of the framework catalysts, thereby suppressing material dissolution and aggregation under harsh electrochemical conditions, and consequently enhancing long‐term stability.^[^ ^28^ ^]^ As an effective dopant in cobalt‐integrated electrocatalysts, the Co–Fe_3_O_4_/IF nanosheets demonstrated superior bifunctional catalytic activity, which can be attributed to the generation of additional active sites, strong synergistic interactions and improved structural stability.^[^ ^24^ ^]^ More importantly, designing trimetallic TM‐based materials with non‐metallic elements can enhance free charge carrier density and improve conductive networks through unpaired d‐orbitals and interfacial electrostatic interactions.^[^ ^29^, ^30^ ^]^ In multi‐metal systems, Co can interact synergistically with Fe, Mo and B. This trimetallic electronic interaction with boron can modulate the charge distribution, facilitating faster electron/proton transfer and stabilizing specific oxidation states favorable for the catalytic WE process.^[^ ^30^ ^]^ Thus, Co‐doping into the FeMoB matrix can be an attractive strategy for developing advanced electrocatalysts with high efficiency and stability under rigorous industrial conditions, which has never been attempted yet.

In this work, Co‐doped FeMoB micro‐petals (MPs) electrocatalyst is successfully synthesized for the first time via a dual‐step hydrothermal approach followed by thermal annealing, as illustrated in Figure S1 (Supporting Information), for industrial‐level green hydrogen generation. The Co/FeMoB MPs demonstrate improved catalytic activity and stability for HER/OER. For overall water electrolysis, the Co/FeMoB MPs exhibit significantly low cell voltages, high current performance, and ampere‐level long‐term stability under various bi‐functional (Co/FeMoB /̸̸ / Co/FeMoB) and hybrid (Pt/C /̸̸ / Co/FeMoB) operational conditions, highlighting its potential for industrial applications. More significantly, a small amount of Co inclusion can optimize adsorption/desorption kinetics, enlarge the electrochemical surface area and increase active sites, consequently enhancing overall catalytic activity. This study provides new insights into doping engineering for the development of advanced multifunctional electrocatalysts aimed at scalable commercial H_2_ production.

## 2 Result and Discussion

### 2.1 Structural and Elemental Properties of Co/FeMoB MPs

**Figure 1:**
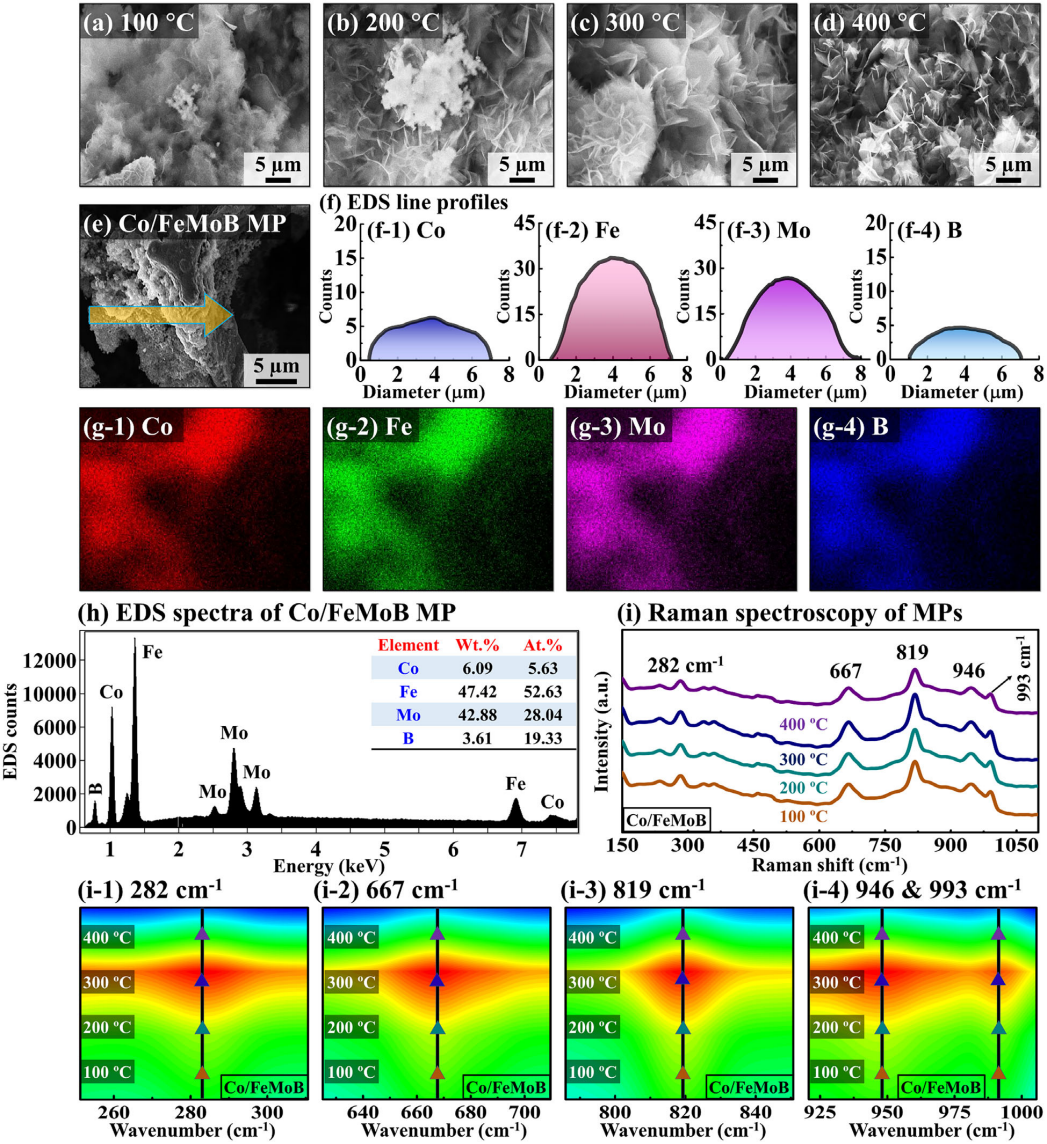
Co‐doped FeMoB micro‐petal (MP) electrodes with post‐annealing temperature optimization. a–d) Scanning electron microscopy (SEM) micrographs of Co/FeMoB MPs. e–f‐4) SEM image and energy dispersive x‐ray spectroscopy (EDS) line‐profiles. g‐1–g‐4) EDS elemental phase maps. h) EDS spectrum with weight and atomic ratios. i–i‐4) Raman spectra and corresponding counter plots of Raman signals.

**Figure 2:**
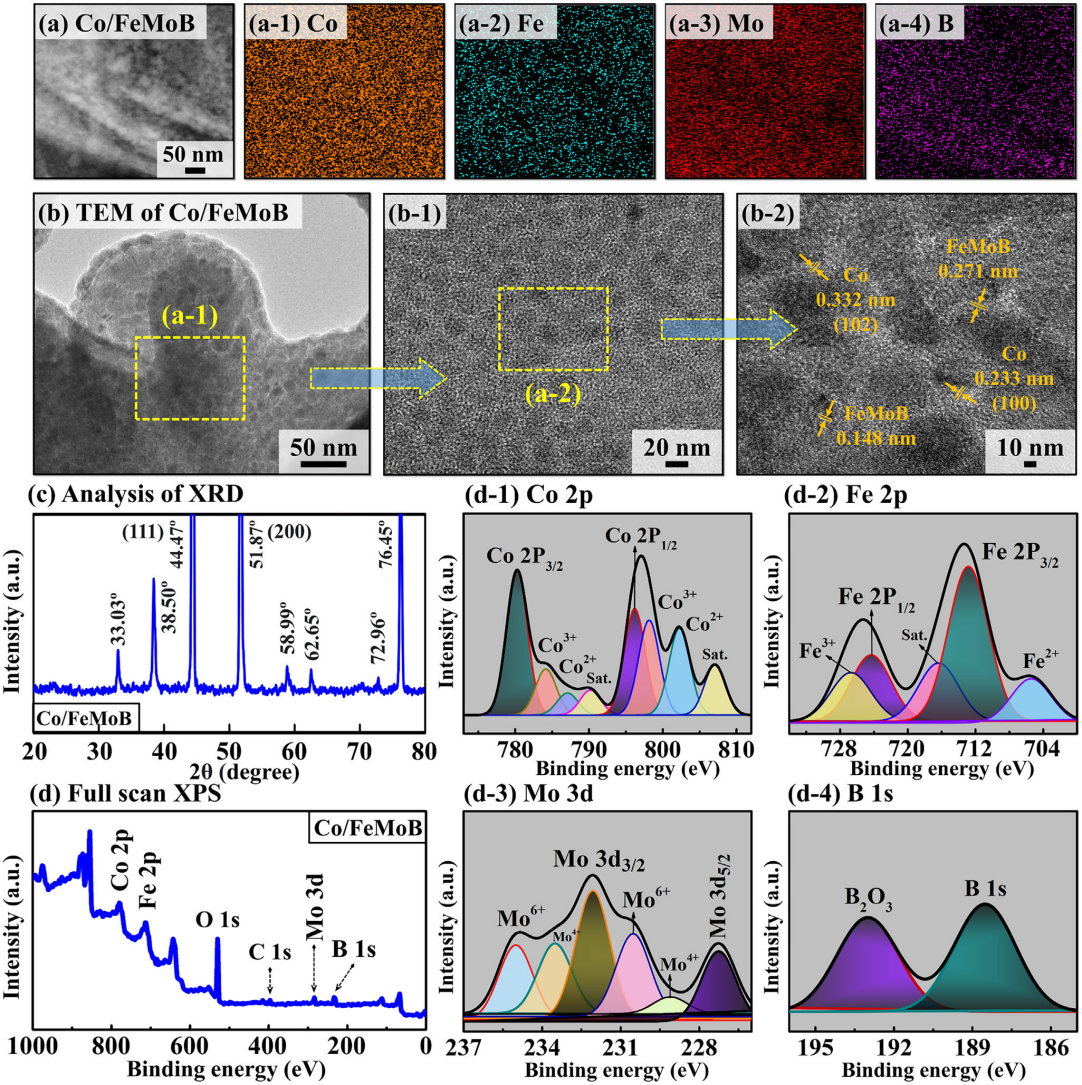
Physical characterization of the best Co/FeMoB MP (300 °C annealed) electrocatalyst. a–a‐4) Cross‐sectional (CS) side‐view EDS phase maps obtained from the electrode interior. b–b‐4) High‐resolution (HR) transmission electron microscopy (TEM). c) X‐ray diffraction (XRD) pattern. d) Full‐scan x‐ray photoelectron spectroscopy (XPS). d‐1–d‐4) High resolution XPS spectra of Co 2p, Fe 2P, Mo 3d and B 1s.

**Figure 3:**
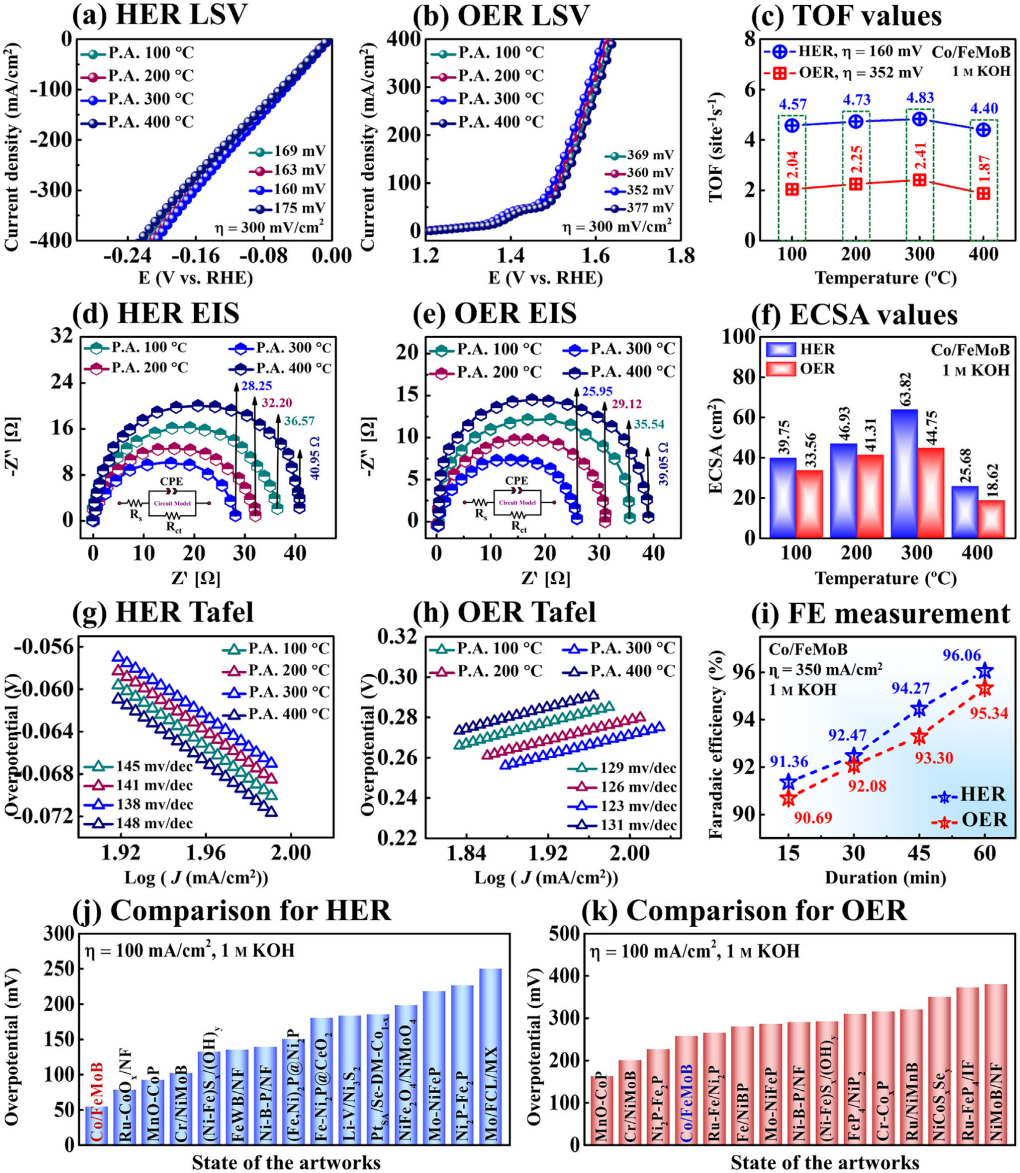
Three‐electrode (3‐E) electrochemical performance of Co/FeMoB MP in 1 M KOH for post‐annealing temperature control set. a–b) Linear sweep voltammetry (LSV) polarization curves for HER/OER with overpotential values. c) HER/OER turnover frequency (TOF). d–e) Electrochemical impedance spectroscopy (EIS) plots for HER/OER. f) HER/OER electrochemical surface area (ECSA) values. g,h) HER/OER Tafel slopes. i) Faradic efficiency (FE) measurement in alkaline media. j–k) HER/OER performance comparison with state‐of‐the‐artworks.

**Figure 4:**
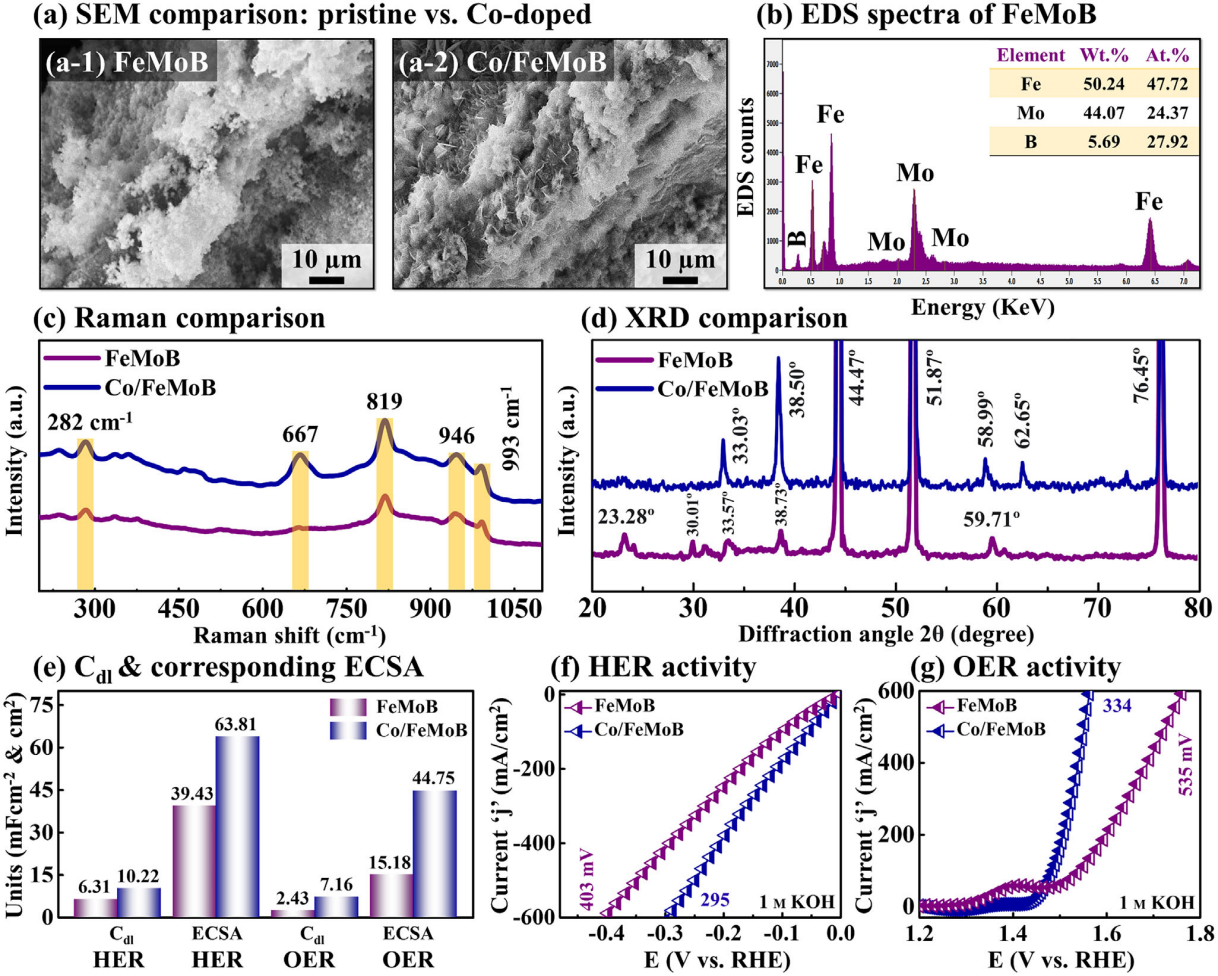
Before/after Co doping comparison and physical/electrochemical characteristics. a) SEM micrographs of pristine FeMoB framework and Co‐doped FeMoB MP. b) EDS spectra of base FeMoB. c) Raman spectroscopy comparison of before/after Co incorporation. d) XRD diffraction pattern comparison e) Electrochemical *C*_dl_ and corresponding ECSA. f–g) HER and OER performance comparison in 1 m KOH.

**Figure** **1** shows the optimization, morphological, and structural properties of the Co/FeMoB MP electrocatalysts with post‐annealing temperature control set. The temperature of the annealing treatment was varied from 100 to 400 °C for 30 min as shown in Figure 1a–d. In the SEM micrographs, the Co‐doped annealed electrodes exhibited well‐defined micro‐petal (MP)‐like surface morphologies, which were maintained up to 400 °C. The highly dense morphology can deliver significant benefits by maximizing the number of electrochemical reaction sites, enhancing structural integrity, and facilitating efficient mass transport during catalysis.^[^ ^31^, ^32^ ^]^ Further, the EDS line‐profiles showed consistent signals and intensity for Co, Fe, Mo, and B in Figure 1e–f‐4, providing clear evidence of Co doping and compositional/elemental uniformity throughout surface structure. Similarly, the EDS color mapping demonstrated the presence of Co, Fe, Mo, and B phases on the surface in Figure 1g‐1–g‐4, confirming successful Co atom incorporation and the homogeneous dispersion of all elements. In addition, the EDS spectral analysis validated the presence of each element along with their weight and atomic content ratios as shown in Figure 1h. The results revealed that a relatively low amount of Co was incorporated into the FeMoB framework (wt.%: 6.09 and at.%: 5.63). Then, Raman spectroscopy was analyzed to investigate the crystallinity and crystal quality of the MP electrodes in Figure 1i. The Co‐doped all the annealed samples (100, 200, 300, and 400 °C) revealed characteristic Raman peaks at 282, 667, 819, 946, and 993 cm^−1^. The observed Raman signals correspond to distinct vibrational modes associated with variations in atomic coordination and bonding characteristics. The peaks at 282 and 993 cm^−1^ can be assigned to B atom vibrations and B─B bonds. The 819 cm^−1^ peak likely corresponds to Mo─B bonding, while the 946 cm^−1^ peak can be associated with stretching vibrations of metal oxides or oxygen‐containing species (M─O/M─O─OH).^[^ ^33^ ^]^ Notably, the appearance of an additional Raman peak at 667 cm^−1^ after doping can be attributed to Co─O vibrational modes, indicating structural changes and effective doping of Co atoms.^[^ ^34^ ^]^ Moreover, the 300 °C MP sample exhibited the strongest Raman band intensity, and this trend was also distinctly visible in the corresponding contour plots in Figure 1i‐1–i‐4. The highest Raman peak intensities, observed for the MP (300 °C), suggested the best crystallinity and overall crystal quality compared to the other samples. Significantly, the best crystal quality suggests more availability of active sites, better electron transportation capability, and stable catalytic surface of the electrode.^[^ ^35^ ^]^ The double‐step hydrothermally synthesized unannealed Co‐doped MP samples may contain structural defects, inner‐site crystallographic mismatches, and atomic‐level dislocations due to forced Co atoms injection under high‐pressure/temperature conditions.^[^ ^36^, ^37^ ^]^ Here, the post‐annealing plays an important role and can help to improve crystallization, adsorption efficiency and lower charge transfer resistance by reducing atomic imperfections, defects, and lattice mismatches.^[^ ^38^, ^39^ ^]^ Conversely, excessive thermal energy during annealing may lead to a loss of crystallinity and degradation of material catalytic properties.^[^ ^30^ ^]^ Overall, the best‐optimized 300 °C MP demonstrated effective Co incorporation along with overall enhanced physical and electrochemical properties. More details on Co‐doped FeMoB electrocatalyst fabrication and related others can be found in Section S‐1.1–S1.3 (Supporting Information).

### 2.2 Physical Characteristics of the Optimized Co/FeMoB MP

**Figure** **2** demonstrates solid‐state structural and compositional analyses of the multifunctional Co/FeMoB MP (300 °C annealed) electrocatalyst by CS‐EDS, TEM, XRD, and XPS techniques. After careful focused ion beam (FIB) preparation, cross‐sectional (CS) EDS and high‐resolution (HR‐TEM) investigations were performed on the interior (micron‐scale depth) of the MP electrode. The CS‐EDS phase maps verified the successful existence of Co, Fe Mo, and B elements as shown in Figure 2a–a‐4. Despite the low content of Co, it was uniformly distributed throughout the micro‐scale internal regions, indicating consistent Co incorporation in Figure 2a‐1. Further, the HR‐TEM analysis was carried out at different nanometer‐scale resolutions to investigate a specific internal structural feature, including atomic‐level crystal structure and phase composition identification in Figure 2b–b‐2. The HR‐TEM analysis revealed that the initially developed FeMoB framework possesses polycrystalline structure, whereas the Co atoms were successfully incorporated as randomly oriented nanocrystals within the matrix. The observed lattice spacings (plane‐to‐plane distances) of 0.148 and 0.271 nm can correspond to the FeMoB phases as shown in Figure 2b‐2. Meanwhile, the interplanar d‐spacing of 0.332 and 0.233 nm can be attributed to Co nanocluster phases. The lattice fringes of Co and FeMoB mostly appearing at the periphery of the dark particles, which is a common phenomenon observed in HRTEM images, are likely due to variations in specimen thickness and electron beam orientation.^[^ ^40^, ^41^ ^]^ The peripheral region can be relatively more thinner and/or crystalline than the central region, allowing better electron transmission and clearer visualization of the atomic lattice fringes in the Co/FeMoB material. Thus, the Co atoms were inserted into the FeMoB crystal matrix as nanoclusters with heterogeneously oriented lattice planes, suggesting an overall polycrystalline nature. Here, the specific crystallographic planes could not be clearly identified due to the unique polycrystalline formation of the material.^[^ ^42^ ^]^ This indicates that Co atoms were successfully doped into the FeMoB matrix via high‐pressure, high‐temperature hydrothermal optimization and subsequent thermal treatment. Overall, the HR‐TEM analysis demonstrated that the MP composition consisted of numerous small crystalline domains, and the internal investigation revealed the polycrystalline nature. The XRD pattern analysis was performed to investigate phase composition and crystal structure, and crystallinity as shown in Figure 2c. The strong diffraction peaks located at 44.47° and 51.87° can be attributed to the (111) and (200) crystal planes originating from the nickel foam (NF) substrate.^[^ ^43^ ^]^ The sample exhibited major characteristic peaks were observed at 33.03°, 38.50°, 58.99°, 62.65°, and 76.45° within the 2θ range, indicating the co‐existence of multiple crystalline phases in the sample material. The presence of multiple XRD peaks can be attributed to the different lattice planes and random orientation of crystallites, characteristic of a polycrystalline composition, which is decently consistent with the TEM observations. After Co doping, the observed slight peak shifts toward lower/higher angles suggested increased lattice constants and expansion MP clusters, indicating partial atomic or ionic substitution into the host FeMoB matrix,^[^ ^44^ ^]^ leading to phase modulation and successful incorporation of Co species.^[^ ^45^ ^]^ According to previous literature, polycrystalline materials often outperform single crystals due to enriched charge recombination capacity, increased active sites, and enlarged ECSA.^[^ ^4^ ^]^

The elemental composition, chemical states, and surface electronic environment of the Co/FeMoB electrocatalyst were investigated using XPS, as shown in Figure 2d–d‐4. The full‐scan XPS spectrum reveals distinct peaks for cobalt (Co 2p), iron (Fe 2p), molybdenum (Mo 3d), and boron (B 1s), verifying the elemental composition of the MP material as shown in Figure 2d. In addition, oxygen‐ and carbon‐containing species (O 1s and C 1s) were commonly detected in the full‐scan XPS spectrum, likely arising from unavoidable surface oxidation induced by air exposure/during hydrothermal synthesis and from binding energy (BE) calibration.^[^ ^42^, ^46^, ^47^ ^]^ One the one hand, the Fe 2p_1/2_ peak exhibited a positive shift of 4.17 eV from its elemental position from 720.10 to 724.27 eV in the high‐resolution Fe 2p spectrum as shown in Figure 2d‐2, indicating electron donation. In addition, the Fe 2p_3/2_ peak showed a substantial positive shift of 5.78 eV. These positive shifts in binding energy suggest strong electronic interactions within the matrix, likely due to electron transfer or redistribution. The peaks located at 705.34 and 726.68 eV can be corresponded to the presence of Fe^2+^ and Fe^3+^ ionic states, respectively. Another shakeup satellite peak can be found at 716.31 eV. Further, the Mo 3d spectrum in Figure 2d‐3 exhibited a negative binding energy shift of 0.75 eV for the Mo 3d_5/2_ peak, indicating electron gain by the Mo species.^[^ ^42^ ^]^ In another characteristic peak was found 232.06 eV. Moreover, the peaks at 229.10 and 233.51 eV can be attributed to Mo⁴⁺ species, corresponding to MoO_2_, while the peaks at 230.51 and 235.00 eV are assigned to Mo⁶⁺ species, likely corresponding to MoO_3_. The coexistence of multiple oxidation states suggests surface oxidation of metallic Mo species. Similar to Mo, the B 1s peak was observed at 188.52 eV, showing a negative shift of 0.88 eV from the pristine handbook value of 189.4 eV in Figure 2d‐4. This negative shift indicates an increase in electron density around the B atoms. Additionally, the peak at 192.98 eV is attributed to B_2_O_3_, suggesting the presence of oxidized boron species (B─O) on the surface. Finally, the dopant Co 2p spectrum demonstrated two distinct peaks corresponding to Co 2p_3/2_ at 780.25 and Co 2p_1/2_ at 796.19 eV, as shown in Figure 2d‐1. The two peaks revealed positive shifts of 1.95 and 3.10 eV compared to the pristine values (778.30 eV for Co 2p_3/2_ and 793.09 for Co 2p_1/2_). These positive binding energy (BE) shifts suggest modulation of the electronic structure and an increase in electron density around the host clusters, which can be attributed to lattice strain within the crystal structure and charge/electron redistribution within the matrix induced by Co doping. The observed electron transfer among Fe, Mo, and B indicates strong ionic interactions, facilitating the formation of the FeMoB framework. Then, the catalytically active Co was doped as nanoscale clusters into the host framework via hydrothermal‐annealing process, increasing local electron density and catalytic HER/OER activity and confirming the successful formation of hybrid Co/FeMoB electrocatalyst.

### 2.3 Characteristics and Mechanisms for Electrochemical HER/OER

**Figure** **3** demonstrates the HER/OER performance and catalytic properties of Co/FeMoB electrocatalysts for post‐annealing temperature‐controlled set using a standard three‐electrode (3‐E) system. The LSV polarization curves of Co‐doped multifunctional electrodes revealed distinct HER/OER activity variations with annealing temperature in 1 m KOH. The HER/OER profiles demonstrated that catalytic performance progressively improved with elevating annealing temperatures from 100 to 300 °C. The optimized annealing can facilitate electrolyte infiltration into the internal sub‐nanostructure and promote efficient release of gaseous H_2_/O_2_ molecules.^[^ ^44^ ^]^ This can contribute to temperature‐dependent structural and compositional modifications that improve catalytic performance.^[^ ^48^ ^]^ Nonetheless, the 400 °C MP electrode exhibited reduced catalytic activity, likely due to excessive thermal disruption of the nano–microstructure. Excessive diffusion energy may be generated during post‐annealing, which can compromise the structural integrity of the crystalline network, leading to degradation and reduced catalytic efficiency as evident at 400 °C.^[^ ^30^, ^48^ ^]^ The 300 °C Co/FeMoB MP sample demonstrated the lowest HER/OER overpotential values of 160/352 mV at 300 mV cm^−2^ as shown in Figure 3a–b, indicating superior electrocatalytic performance. Further, the turnover frequency (TOF) quantifies the generation rate of H_2_ and O_2_ molecules at each active site per unit time, serving as a key indicator of intrinsic catalytic activity.^[^ ^49^ ^]^ The 300 °C MP electrode exhibited the highest HER/OER TOF values (4.83/2.41 site^−1^s^−1^) in Figure 3c, demonstrating effective activation of catalytic sites and faster reaction kinetics for H_2_/O_2_ evolution.^[^ ^50^ ^]^ A detailed TOF calculation‐related text can be found in Section S‐1.7 (Supporting Information). The EIS was conducted in 1 M KOH at 15 mA cm^−2^ along with the corresponding equivalent Randles circuit diagram to evaluate the charge transfer characteristics and interfacial conductivity of the MP electrocatalysts in Figure 3d–e. Here, the *R*_s_ represents the solution resistance, *R*_ct_ denotes the charge transfer resistance, and CPE refers to the constant phase element in the Randles circuit model.^[^ ^10^ ^]^ The EIS Nyquist plots were analyzed to extract the *R*_ct_ values; where a smaller semicircle radius reflects lower resistance to electron transport, indicating accelerated charge‐transfer dynamics at the electrode–electrolyte boundary.^[^ ^51^ ^]^ Remarkably, the 300 °C MP showed the smallest semicircle, corresponding to the lowest *R*_ct_ values of 28.25/25.95 Ω for HER/OER, indicating superior interfacial charge/electron transportation capability.^[^ ^52^ ^]^ The electrochemical surface area (ECSA) was obtained based on the double‐layer capacitance (*C*_dl_) using the equation: $\mathrm{ECSA}=\frac{\mathrm{Cdl}}{\mathrm{Cs}}$, where Cs (0.04 mF cm^−2^) represents the specific capacitance of a flat electrode surface. The ECSA values of the MP annealed samples are shown in Figure 3f, where the 300 °C MP exhibited the highest values (63.82/44.75 cm^2^ for HER/OER), indicating a significantly expanded catalytically active surface area. The electrocatalytic activity of materials is strongly influenced by their ECSA, and the highest ECSA values can be attributed to their well‐defined micro‐petal morphology, abundant accessible active sites, and excellent intrinsic catalytic properties.^[^ ^53^ ^]^ In addition, the ECSA is directly proportional to *C*_dl_ values. The *C*_dl_ values were derived from cyclic voltammetry (CV) profiles recorded at multiple scan rates within the non‐faradaic potential region, and from this, the ECSA was calculated. The corresponding CV curves of the Co/FeMoB samples for HER/OER are presented in Figures S22 and S23 (Supporting Information). The 300 °C MP exhibited the highest C_dl_ values of 10.21 and 7.16 mF/cm^2^ for HER/OER in Figures S24 and S25 (Supporting Information), indicating improved ion diffusion and the largest ECSA resulting from higher accessibility to the active sites.^[^ ^53^, ^54^ ^]^ The Tafel slopes were adapted from the linear regions of the LSV plots to gain insights into the reaction kinetics, using the Tafel equation:^[^ ^55^ ^]^ η = a + b log j, where *“b”* stands for the Tafel slope and *“j”* is for the current density in Figure 3g–h. Among the MP samples, the 300 °C MP showed the lowest Tafel slopes for HER/OER (138/123 mV dec^−1^), indicating faster reaction kinetics and catalytic efficiency.^[^ ^56^ ^]^

The HER mechanism involves the Volmer step (H_2_O + e^−^ + M → M─H*_ads_ + OH^−^) followed by either the Heyrovsky step (M─H*_ads_ + H_2_O + e^−^ → H_2_ + M + OH^−^) or the Tafel step (M─H*_ads_ + M–H*_ads_ → H_2_ + 2 M), where H*_ads_ and M represent proton absorption/desorption HER intermediates and metallic sites, respectively.^[^ ^57^ ^]^ A high surface coverage of M– H*_ads_ intermediates facilitate the Tafel pathway for H_2_ evolution. In parallel, the proceeds through four electron‐transfer steps:^[^ ^46^ ^]^ i) M + OH^−^ → M–OH* + e^−^, ii) M─OH* + OH^−^ → M─O* + H_2_O + e^−^, iii) either 2M─O* → 2 M + O_2_ or M─O* + OH^−^ → M─OOH* + e^−^ and iv) M─OOH* + OH^−^ → O_2_ + H_2_O + M + e^−^. These multi‐step redox processes involve the formation and transformation of oxygen‐containing intermediates (M─OH*, M–O*, and M─OOH*) ultimately leading to O_2_ generation.^[^ ^57^ ^]^ The high density of electroactive species M (Co, Fe, and Mo) enhances adsorption–desorption kinetics and lowers the reaction energy barrier, which significantly facilitates H_2_O dissociation.^[^ ^53^ ^]^ In addition, a very minor redox peak commonly observed in the OER LSV curves as observed in Figure 3b may result from the transformation of metallic species into higher valence states.^[^ ^58^ ^]^ The rapid redox conversion of adsorbed oxygenated intermediates such as O*, OH*, and OOH* is also likely to mitigate the sluggish kinetics of the rate‐determining step (RDS), thereby facilitating enhanced OER activity.^[^ ^59^ ^]^ Therefore, all the physical and electrochemical results discussed above demonstrate that the 300 °C Co/FeMoB MP sample represents the optimal condition in the final temperature‐controlled annealing optimization set. Additional electrochemical HER/OER characteristics (e.g., CA, LSV versus CV current response, CV‐repeatability, CP‐stability, LSV comparison, pH‐dependent LSV comparison with benchmarks and different pH TOF activity) of the optimal Co/FeMoB MP electrocatalyst are provided in Section S‐1.8 (Supporting Information). Moreover, the intrinsic activity toward the HER/OER was evaluated by normalizing the geometric current density to the electrochemically active surface area (ECSA) according to the equation: jECSA = j_geom_/ECSA, where j_geom_ represents the current density based on the geometric surface area.^[^ ^60^ ^]^ The ECSA‐normalized activity of the optimized Co/FeMoB (300 °C‐annealed) catalyst for HER/OER is presented in Figure S37 (Supporting Information), demonstrating its intrinsic catalytic properties independent of surface area effects. Further, the Faradaic efficiency (FE) of Co‐doped FeMoB was measured using the gas‐displacement method in 1 M KOH in Figure 3i. The MP catalyst demonstrated FE values of 96.06% and 95.34% for HER/OER, respectively. A photograph of the water‐gas displacement setup is provided in Figure S38 (Supporting Information). The experimentally observed H_2_/O_2_ volume ratio remained close to the theoretical value of 2:1 over various time intervals (Figure S39, Supporting Information), indicating efficient conversion with negligible side reactions, supporting its practical applicability. The detailed calculation and procedures of FE are available in Section S‐1.9 (Supporting Information). Further, the HER/OER performances were benchmarked against state‐of‐the‐artworks at 100 mA cm^−2^ in 1 M KOH as shown in Figure 3j–k. The Co‐doped multifunctional catalyst was ranked as the third‐best for both HER/OER, highlighting its potential as a promising multifunctional electrocatalyst. Moreover, the HER/OER performance metrics are summarized in Tables S1–S3 (Supporting Information), and comparative analysis with advanced electrocatalysts at high current densities (100, 300 and, 600 mA cm^−2^) is detailed in Tables S8 and S9 (Supporting Information).

### 2.4 Effect of Co Doping on FeMoB Framework Catalyst

The physical and electrochemical characteristics of the FeMoB framework before/after cobalt (Co) doping were comprehensively compared and characterized using SEM, EDS, Raman spectroscopy, XRD, EIS, *C*_dl_, ECSA, LSV, ECSA‐normalized LSV, CP‐stability, and HER/OER comparison as shown in **Figure** **4** and Figures S40–S47 (Supporting Information). The SEM micrographs revealed slight morphological differences between the undoped FeMoB and Co‐doped FeMoB samples as observed in Figure 4a‐1–a‐2. The observed variations can be attributed to the integration of Co atoms into the FeMoB framework, facilitated by the hydrothermal reaction and subsequent thermal annealing. The EDS spectra of FeMoB and Co/FeMoB shown in Figure 4b and Figure S40 (Supporting Information), confirmed the successful incorporation of Co into the FeMoB matrix with distinct Co signals of 5.88 wt.% and 5.37 at.%. The Raman analysis before/after Co doping is shown in Figure 4c. The pristine FeMoB exhibited characteristic Raman bands at 282, 819, 946, and 993 cm^−1^. After doping, the Co/FeMoB showed increased peak intensities along with an additional strong signal at 667 cm^−1^, which likely corresponds to the formation of Co–O vibrational modes.^[^ ^34^ ^]^ These changes indicate the new M─O bonding environments and local structural modifications. The XRD diffraction patterns of doped and undoped samples are compared in Figure 4d, where both electrodes showed strong peaks at around 43° and 52°. These specific peaks can be assigned to the (111) and (200) crystal planes of the NF substrate. The FeMoB electrode showed diffraction peaks at 23.25°, 30.01°, 33.48°, 38.81°, 59.74°, and 76.45° in the 2θ range, whereas the Co/FeMoB sample exhibited enhanced intensities with minor peak shifts. In addition, the disappearance of 23.28° and 30.01° peaks along with the emergence of a new peak at 62.65° indicate an overall structural reconfiguration induced by Co doping. These phase transitions can be attributed to the low‐level of Co incorporation into the FeMoB host framework, consistent with the Raman observations. Importantly, the increased XRD and Raman peak intensities indicate improved crystallinity and crystal quality of the Co/FeMoB matrix, which is highly beneficial for facilitating the catalytic HER/OER activity. To understand the electrochemical behavior before/after Co doping, the EIS analysis was conducted and compared between the FeMoB and Co/FeMoB electrodes as shown in Figure S41 (Supporting Information). The Co‐doped FeMoB exhibited lower charge transfer resistance (R_ct_) values compared to the undoped FeMoB for both HER (28.25 versus 34.50 Ω) and OER (25.95 versus 31.43 Ω) as shown in Figure S41a,b (Supporting Information). These reductions in *R*_ct_ values indicate enhanced electrical conductivity and improved charge transfer efficiency resulting from Co doping. Furthermore, the C_dl_ values obtained from the CV curves (S42 and S43, Supporting Information) and the corresponding ECSA were evaluated and compared, as shown in Figure 4e. The pre‐catalyst FeMoB exhibited much lower *C*_dl_ for HER/OER (6.31/2.43 mF cm^−2^) than doped catalyts Co/FeMoB MP for HER/OER (10.22/7.16 mF cm^−2^). Due to its direct proportional relationship with C_dl_, the ECSA exhibited significantly higher values for HER/OER (FeMoB: 39.43/15.18 versus Co/FeMoB: 63.81/44.45 cm^2^). This considerable enhancement in ECSA indicates an increased number of catalytic active sites and improved intrinsic activity attributed to Co doping into the FeMoB host catalyst. Finally, the HER/OER LSV performances were compared in 1 M KOH as shown in Figure 4f–g and Figure S44 (Supporting Information). The Co‐doped FeMoB MP electrode showed markedly improved activity, requiring only 295/334 mV for HER/OER at 600 mA cm^−2^, compared to 403/535 mV for undoped FeMoB electrode. The reduced overpotentials (by 108 mV for HER and 201 mV for OER) confirm the effective incorporation of Co nanoclusters into the FeMoB matrix, which enhances the catalytic reaction kinetics through the synergistic effects of the multi‐element composition.^[^ ^61^ ^]^ In particular, the Co/FeMoB catalyst exhibited significantly higher ECSA‐normalized LSV curves than FeMoB as illustrated in Figure S45a,b (Supporting Information), highlighting the beneficial effect of Co doping on the intrinsic catalytic properties.^[^ ^62^ ^]^ Furthermore, the HER/OER catalytic stability of FeMoB and Co/FeMoB electrodes was evaluated at 100 mA cm^−2^ current density in 1 M KOH as shown in Figure S46 (Supporting Information). The FeMoB framework catalyst exhibited stable HER/OER performance for 60 h, while the Co/FeMoB material demonstrated superior stability, maintaining consistent stable responses for 100 h under the same operational conditions. This enhanced electrochemical stability can be ascribed to the incorporation of Co heteroatoms into the FeMoB framework via hydrothermal synthesis and thermal treatment, which improves the structural integrity and catalytic activity. Further, the HER/OER performances of Co/FeMoB MP were directly compared with those of recently reported Co‐doped transition metal (TM)‐based electrocatalysts at 100 mA cm^−2^ (Figure S47 and Table S10, Supporting Information), where Co/FeMoB MP ranked first for HER and third for OER. Overall, the incorporation of metallic Co atoms, serving as electron donors, enhances the catalytic process by promoting the rapid formation of reaction intermediates, which provide stronger water adsorption sites and reduce ion diffusion pathways for EWE activity.^[^ ^23^ ^]^

### 2.5 Bifunctional System and Natural Waters Application

**Figure 5:**
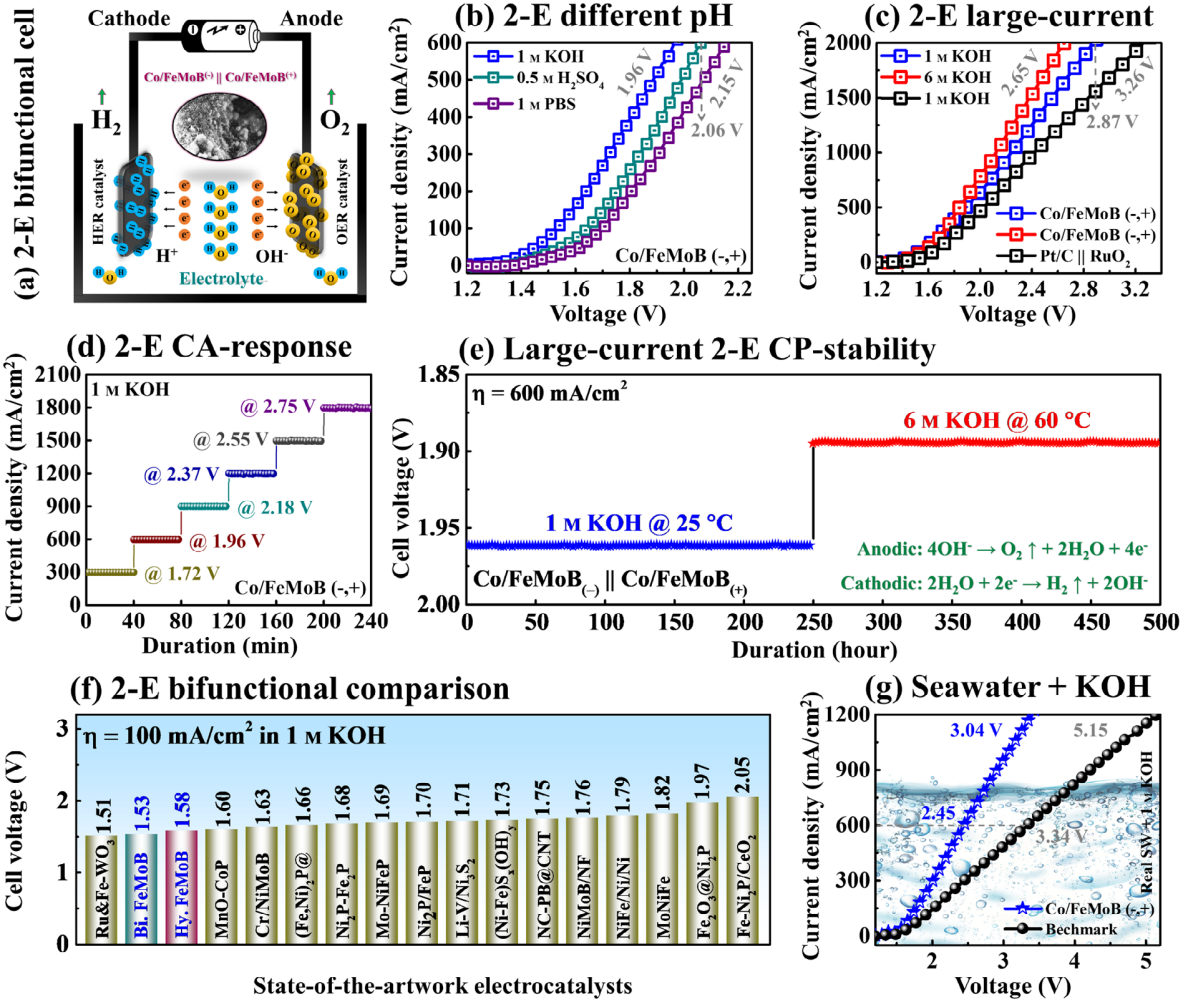
Two‐electrode (2‐E) bifunctional characteristics under various electrochemical conditions. a) Schematic view of the bifunctional cell construction of Co/FeMoB (–,+). b) 2‐E LSV performance in different pH electrolytes as 1 M KOH, 0.5 M H_2_SO_4,_ and 1 M PBS. c) Large‐current‐density (LCD) activity comparison in 1/6 M KOH solutions. d) Steady‐state chronoamperometry (CA) response in 1 M KOH. e) Long‐term stability assessment for 250 h by two‐step chronopotentiometry (CP) operation. f) 2‐E performance comparison with recently reported advanced electrocatalysts. g) 2‐E overall water electrolysis (OWE) in alkaline seawater (SW + 1 M KOH).

**Figure** **5** demonstrates the overall water electrolysis (OWE) performance of the bifunctional Co/FeMoB‐MP catalyst in a two‐electrode (2‐E) configuration in different operational conditions. Inspired by the impressive HER/OER, the Co‐doped MPs were used as both the cathode and anode: Co/FeMoB_(−)_ / / Co/FeMoB_(+)_, and the performance was benchmarked against the conventional Pt/C_(−)_ / / RuO_2(+)_ system. The schematic of the 2‐E bifunctional OWE cell configuration is shown in Figure 5a. The Co‐doped bifunctional system outperformed the benchmark across all pH conditions as shown in Figure 5b. Specifically, the Co/FeMoB (– /̸̸ / +) configuration exhibited low cell voltages of 1.81 V in 1 m KOH, 2.06 V in 0.5 m H_2_SO_4,_ and 2.15 V in 1 m PBS at 600 mA cm^−2^. In contrast, the benchmark system required higher voltages of 2.03, 2.56, and 2.95 V under the same conditions (Figure S48, Supporting Information). These results highlight the Co/FeMoB catalyst rapid reaction kinetics and excellent OWE performance across a wide pH range. For industrial applications, electrocatalysts must perform reliably under practical conditions such as large‐current‐density (LCD) and long‐term operation.^[^ ^63^ ^]^ The LCD adaptability with stable performance is a critical requirement, as it accelerates hydrogen production, reduces capital costs, and improves economic feasibility for commercial H_2_ production. Notably, the OWE performance was evaluated at LCD up to 2000 mA cm^−2^ in alkaline media as shown in Figure 5c. The bifunctional Co/FeMoB MP catalyst outperformed the benchmark system in both 1/6 m KOH, demonstrating excellent potential for industrial‐scale large‐current applications. Notably, the Co‐doped electrode exhibited a low cell voltage of 2.87 V at an ampere‐level current density of 2000 mA cm^−2^ in 1 m KOH, compared to the benchmark value of 3.26 V. In addition to using high alkaline concentrations, industrial electrolysis processes generally employ elevated temperatures to enhance reaction kinetics. Under such conditions, the Co‐doped MP demonstrated further improved performance in 6 m KOH at 60 °C, achieving a reduced cell voltage of only 2.65 V at 2,000 mA cm^2^. These results highlight the superior OWE performance of the Co/FeMoB (– /̸̸ / +) settings, which outperforms many previously reported systems in terms of high‐current and low cell voltage operation, underscoring its strong potential for industrial H_2_O electrolysis applications. In addition, the high‐alkaline concentration provides a greater abundance of conductive ions (K⁺/OH^−^), which enhances ionic conductivity, accelerates electron transport, and lowers the activation energy for WE.^[^ ^30^ ^]^ The elevated temperatures further improve performance by reducing diffusion lengths, enhancing electrolyte–electrode interactions, increasing reactant availability, and accelerating the kinetics of H_2_O oxidation.^[^ ^64^ ^]^

In addition to catalytic activity, long‐term stability at high current densities is a critical requirement for large‐scale OWE. To assess the bifunctional (– / / +) stability, the CA measurements were performed at a series of applied voltages: 1.72, 1.96, 2.18, 2.37, 2.55, and 2.75 V in Figure 5d. During high current operation, substantial H_2_/O_2_ gas bubble accumulation on the electrode surfaces can increase ohmic resistance and hinder mass transport by forming physical barriers.^[^ ^65^ ^]^ Notably, the Co‐doped electrode exhibited stable CA responses for OWE, which correlated well with the corresponding LSV current profiles (Figure S49, Supporting Information). This consistency suggests efficient bubble‐release behavior with minimal adhesion or blockage on the MP surface. After 1500 cyclic CV operations at scan rate of 100 mV s^−1^ over 12 h, the LSV curves show no noticeable change, indicating catalytic repeatability for HER/OER (Figure S50, Supporting Information). Importantly, the bifunctional (– /̸̸ / +) system demonstrated outstanding dual‐stage long‐term stability at 600 mA cm^−2^ in both 1/6 m KOH (≈25/60 °C) alkaline solutions under harsh conditions, maintaining stable performance for over 250 h (>10 days) as shown in Figure 5e. These results indicate the catalyst's robust mechanical strength, strong catalytic activity, and excellent thermal stability, demonstrating its suitability for industrial applications even under elevated temperatures without significant degradation. The industrial‐level durability of the Co‐doped FeMoB MP can be attributed to the balanced adsorption/desorption kinetics, metallic protective M─O/M─O─OH layer formation, improved corrosion resistance, and enlarged ECSA.^[^ ^66^ ^]^ Furthermore, the OWE activity is compared against state‐of‐the‐art catalysts at 100 mA cm^−2^ in 1 m KOH as presented in Figure 5f. The Co/FeMoB (– /̸̸ / +) MP exhibited highly competitive activity, ranking as the second‐best bifunctional electrocatalyst among the compared systems. The corresponding comparisons of top‐performing electrodes at higher current densities (100, 500, and 1000 mA cm^−2^) are summarized in Table S11 (Supporting Information). Motivated by its excellent catalytic performance in neutral (PBS) electrolyte, the 2‐E OWE configuration was further subjected to evaluation in alkaline natural seawater (SW + 1 m KOH) as presented in Figure 5g. The bifunctional (– /̸̸ / +) demonstrated exceptional catalytic performance in alkaline seawater electrolysis (SWE), achieving a significantly reduced overall cell voltage of 3.04 V at 1,200 mA cm^−2^, substantially outperforming conventional benchmarks, which required 5.15 V under the same conditions. This performance significantly exceeds the reference catalysts, underscoring the (– /̸̸ / +) system's potential for large‐scale SWE and sustainable real‐world H_2_ production. Although the SWE performance was inferior to that observed in deionized (DI) water, the system was operated in SW + KOH to enhance ionic conductivity and accelerate reaction kinetics. Generally, the SWE remains inherently challenging due to the complex matrix of dissolved ions (e.g., Ca^2+^, Mg^2+^, Na^+^, Cl^−^, F^−^, and Br^−^), high salt concentration (NaCl ≈ 0.5 m), and presence of various contaminants, including particulates, microorganisms, and organic matter.^[^ ^67^ ^]^ The insoluble white precipitation observed during electrolysis may be attributed to the formation of Mg(OH)_2_/Ca(OH)_2_. These factors can cause the loss of active species, electrode passivation and promote competitive side reactions such as Cl_2_ evolution and ultimately reduce the performance.^[^ ^68^ ^]^ However, the Co/FeMoB (– /̸̸ / +) MP demonstrated superior performance relative to the benchmarks in both bare seawater (SW) and alkaline river water (Figure S51, Supporting Information). Furthermore, the MP electrode exhibited remarkable durability for 25 h at 300/500 mA cm^−2^ (Figure S52, Supporting Information), highlighting its strong corrosion resistance and surface stability against diverse ions, chemicals, and organic species. An additional bifunctional stability can be found in Figure S53 (Supporting Information).

### 2.6 Hybrid OWE Characteristics of Co/FeMoB MP

**Figure 6:**
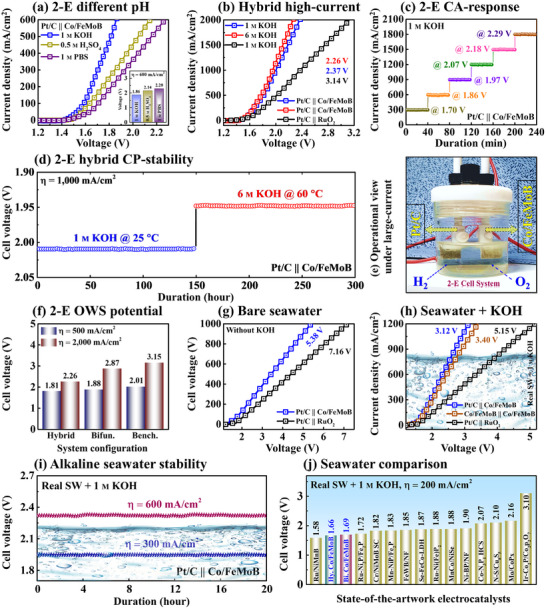
2‐E hybrid configuration activity of Pt/C_(−)_ /̸̸ / Co/FeMoB_(+)_. a) 2‐E LSV performance in different pH media. b) Hybrid LCD performance comparison in 1/6 M KOH. c) State‐state CA in 1 M KOH. d) Dual‐step CP stability tests in alkaline solutions. e) Real‐view image of hybrid cell configuration. (f) OWE potential comparison for different 2‐E cell systems. g) 2‐E hybrid LSV activity in bare seawater (SW). h) OWE performance in alkaline mixed seawater. i) Double‐step stability operation in SW + 1 M KOH. j) State‐of‐the‐art electrocatalysts comparison in alkaline seawater.

**Figure** **6** shows 2‐E hybrid OWE activity for advanced industrial‐scale hydrogen production, where Pt/C (cathode) was paired with Co/FeMoB (anode) to construct a hybrid cell as Pt/C_(−)_ /̸̸ / Co/FeMoB_(+)_ based on the superior OER properties of the MP catalyst. The hybrid system exhibited lower cell voltages (alkaline: 1.86, acidic: 2.14, and neutral: 2.28 V) at 600 mA cm^−2^ as shown in Figure 6a, significantly surpassing the benchmarks in all pH media (Figure S54, Supporting Information). Further, the 2‐E LSV curve extended up to 2000 mA cm^−2^ LCD in 1/6 m KOH (25/60 °C), where the Co/FeMoB hybrid system delivered ultra‐low cell voltages of 2.37/2.26 V, significantly outperforming the benchmark value of 3.14 V in Figure 6b. The hybrid exhibited stable CA performance under various operational voltages of 1.70, 1.86, 1.97, 2.07, 2.18, and 2.29 V in Figure 5c, and the CA/LSV comparisons showed minimal current differences (Figure S55, Supporting Information), indicating better configurational stability. The polarization curves of the Co‐doped hybrid setting remained unchanged after 1500 CV cycles (≈12 h) in both 1/6 m KOH (Figure S56, Supporting Information), indicating excellent LCD repeatability. Additionally, the hybrid setup demonstrated multi‐step ampere‐level (1000 mA cm^−2^) stability for over 150 h in both 1/6 M KOH in Figure 6d, confirming stability for alkaline H_2_ evolution. The use of industrially relevant high‐concentration electrolyte (6 m KOH) at elevated temperature (60 °C) demonstrates the successful construction of the hybrid system and improved large‐scale electrolysis capability.^[^ ^69^ ^]^ A real‐time view of the hybrid cell operating at LCD is presented in Figure 6e, and OWE performance comparisons of the hybrid setting with bifunctional and benchmark systems confirmed that Co/FeMoB MP is a promising, low‐cost, and efficient alternative to RuO_2,_ as shown in Figure 6f. Finally, the hybrid system was tested in natural waters, where the MP electrode outperformed the benchmark in fresh SW, requiring only 5.38 V at 1000 mA cm^−2^ as shown in Figure 6g. Furthermore, the hybrid Co/FeMoB exhibited a remarkably low cell potential of 3.12 V, surpassing both bifunctional/benchmark systems (3.40/5.15 V) in SW + KOH at LCD as observed in Figure 6h. Similarly, the hybrid MP outperformed the benchmarks in bare SW and RW + KOH (Figure S57, Supporting Information). The dual‐step CP tests at 300/600 mA cm^−2^ for 20 h in SW + KOH confirmed seawater robustness of the hybrid setup in Figure 6i. The seawater activities of the hybrid and bifunctional Co/FeMoB systems in SW + KOH were compared with state‐of‐the‐art works in Figure 6j and Table S12 (Supporting Information), ranking them as the 2nd and 3rd best electrodes at 100 mA cm^−2^, highlighting their status as top‐ranked electrocatalysts. The post‐stability physical and electrochemical characterizations, including SEM, XPS, Raman, XRD, and LSV are provided in Figures S58–S64 and Section S‐1.10 (Supporting Information).

## 3 Conclusion

The Co/FeMoB micro‐petal (MP) electrocatalyst was synthesized via a dual‐step hydrothermal reaction followed by post‐annealing, whereas the FeMoB framework was first prepared and then Co was systematically doped to enhance HER/OER kinetics, consequently accelerating the overall water‐splitting process. The incorporation of Co endowed the material with excellent intrinsic properties, optimized adsorption/desorption kinetics, and strong electrocatalytic robustness. The optimized MP electrode showed excellent HER/OER overpotentials of 54/257 mV at 100 mA cm^−2^ in 1 m KOH, outperforming many state‐of‐the‐art electrocatalysts. More importantly, the Co/FeMoB (−,+) exhibited ultra‐low cell voltage of 1.54 V at 100 mA cm^−2^ in 1 m KOH, outperforming the benchmark up to 2000 mA cm^−2^. Also, it maintained long‐term stability for 250 h at 600 mA cm^−2^ under extreme industrial conditions (1/6 m KOH, 25/60 °C), demonstrating strong anti‐corrosion properties. Along with excellent OER, the prepared hybrid system delivered significantly low cell voltages of 2.37/2.26 V at 2000 mA cm^−2^ in 1/6 m KOH, respectively, with stable operation. The superior OWE properties can be attributed to efficient charge transfer, an increased number of active sites, and enlarged electrochemical surface area. Overall, this work provides key insights into designing stable and advanced electrocatalysts, offering a promising alternative to traditional benchmarks and advancing the future hydrogen economy.

## Conflict of Interest

The authors declare no conflict of interest.

## Author Contributions

**M.H.J**. contributed to methodology, investigation, data curation, formal analysis, and validation, wrote the original draft, and wrote, reviewed, and edited the final manuscript. **S.A.D. carried out** the investigation and data curation. **M.N**., **A.H., and S.L**. carried out the investigation and data curation. **J.L**. contributed to conceptualization, project administration, and supervision and acquired funds, wrote‐reviewed, and edited the final manuscript.

## Supporting information



Supporting Information

## Data Availability

The data that support the findings of this study are available from the corresponding author upon reasonable request.
