# Supplementary material for: Micro‐Petal Cobalt‐Doped FeMoB Framework for Efficient Hydrogen Generation in Ampere‐Level Water Electrolysis
Source: Small. 2025 Nov 24;22(3):e11038. doi: 10.1002/smll.202511038 (PMC12802543; doi:10.1002/smll.202511038)
Supplement: Supplementary file 1 — Supporting Information [file SMLL-22-e11038-s001.docx]

**Supplementary Information (SI)**

**Micro-Petal Cobalt-Doped FeMoB Framework for Efficient Hydrogen Generation in Ampere-Level Water Electrolysis**

Mehedi Hasan Joni^1^, Sumiya Akter Dristy^1^, Md Najibullah^1^, Md. Ahasan Habib^1^, Shusen Lin^1^ and Jihoon Lee^1^*

^1^Department of Electronic Engineering, College of Electronics and Information, Kwangwoon University, Nowon-gu Seoul, 01897, South Korea

Correspondence: Jihoon Lee*, E-mail: [jihoonlee@kw.ac.kr](mailto:jihoonlee@kw.ac.kr)

**S-1. Detailed Experimental Section**

**S-1.1. Chemical and Precursors Preparation**

**S-1.2. Base Electrode FeMoB Fabrication**

**S-1.3. Synthesis of Co-Doped FeMoB MP**

**S-1.4. Physical Characterization**

**S-1.5. Electrochemical Characterization**

**S-1.6. Benchmark HER/OER Electrodes**

**S-1.7. Turnover Frequency (TOF) Calculation**

**S-1.8. HER/OER Stability and Different pH Activity**

**S-1.9. Faradic Efficiency (FE) Measurement**

**S-1.10. Post-Stability Characterization of Co/FeMoB MP**

**S-1.1. Chemicals and Precursors Preparation**

All analytical-grade chemicals and reagents were sourced from Sigma-Aldrich (USA), and utilized directly without further purification. In the first step, the base FeMoB electrode precursor solution was formulated by dissolution of iron (III) nitrate nonahydrate (Fe(NO_3_)_3_•9H_2_O), sodium molybdate dihydrate (Na_2_MoO_4_ •2H_2_O) and boric acid (H_3_BO_3_) in 30 mL of deionized (DI) water as sources of Fe, Mo and B, respectively. Urea (CH_4_N_2_O) and ammonium fluoride (NH_4_F) were added as surface-active agents to enhance solution conductivity and stabilize the reaction process. In the second step, cobalt nitrate nonahydrate (Co(NO_3_)_2_•9H_2_O) was dissolved of DI water (30 mL) and used as the Co dopant precursor. The Co atoms were incorporated into the FeMoB base template to enhance electrocatalytic activity, generate high density of active sites and improve the overall conductivity of the electrode. Natural river water (RW) and seawater (SW) were taken from the Han River in Seoul and the Yellow Sea in Incheon, South Korea, respectively. These natural waters were used after undergoing filtration and sedimentation.

**S-1.2. Base Electrode FeMoB Fabrication**

The Co-doped FeMoB micro-petal (Co/FeMoB-MP) electrode was synthesized through a two-step hydrothermal strategy, followed by a thermal annealing process as illustrated in Fig. S1. Firstly, bimetallic FeMoB micro-cloud cluster (MCC) electrode was fabricated on a three-dimensional (3-D) porous nickel foam (NF) substrate using a straightforward hydrothermal approach. Secondly, Co doping was introduced on the FeMoB template through a subsequent hydrothermal reaction and the resulting electrode was thermally annealed in a rapid thermal processing (RTP) chamber. The 3-D porous NF served as a conductive substrate for the overall fabrication of the Co-doped FeMoB MP electrode. To begin with, the bare NF was cut into 1 cm × 1 cm pieces and ultrasonically cleaned in 6 M hydrochloric acid (HCl) solution for 20 minutes, followed by thorough rinsing with DI water to remove surface contaminants. The morphological, elemental and electrochemical characterizations of the NF substrate are provided in Figs. S2–3. The NF exhibited HER/OER overpotentials of 296/780 mV at 50 mA/cm^2^. This low electrochemical HER activity in 1 M KOH was primarily attributed to the absence of catalytically active species on the bulk NF substrate, which could be the reason for the roughly nonlinear response observed in the polarization curve.^[1,2]^

For the hydrothermal synthesis of the FeMoB MCC base framework electrode, a combination of Fe-, Mo- and B-containing precursors, specifically iron (III) nitrate nonahydrate (Fe(NO_3_)_3_•9H_2_O), sodium molybdate dihydrate (Na_2_MoO_4_•2H_2_O) and boric acid (H_3_BO_3_) were used along with urea (CH_4_N_2_O) and ammonium fluoride (NH_4_F) as growth controlling surface-modifying agents. The precursor materials were subjected to solubilization in 30 mL of DI water and magnetically stirred until completely dissolved, and the pre-cleaned NF substrate was immersed in the resulting solution. The mixture solution was then placed in a Teflon-lined stainless-steel autoclave and subsequently subjected to a hydrothermal synthesis process at 180 °C for 12 hours using a microwave-assisted system. The fabricated FeMoB electrode, serving as a base template framework, was removed from the deposition medium and then thoroughly rinsed with DI water and air-dried for further experimental uses. The morphological, elemental and electrochemical characterizations are presented in Figs. S4–7. The bare FeMoB framework exhibited micro-cloud-cluster (MCC)-like surface morphology, which is favorable for high OER/HER catalytic activity and structural stability in Fig. S4(a). The EDS spectra confirmed the presence of Fe, Mo and B elements in Fig. S4(b). Furthermore, the EDS mapping and line profile analyses verified their uniform and homogeneous distribution throughout the structure as shown in Fig. S5. Raman and XRD characterizations of the FeMoB electrode as shown in Fig. S6. Further, the MCC electrode demonstrated HER/OER overpotentials of 230/408 mV at 300 mA/cm^2^ in Fig. S7. The enhanced catalytic activity of the base FeMoB framework is driven by the synergistic interplay of its multicomponent composition, presence of abundant active sites and robust structural integrity.

**S-1.3. Fabrication of Co-Doped FeMoB MP**

The morphologically compact FeMoB micro-cloud cluster electrode was subsequently used as a template for Co doping via a hydrothermal reaction. The bimetallic FeMoB can serve as an excellent doping template due to its intrinsically high HER/OER performance and structural durability. For instance, iron (Fe) is well known for its excellent electrocatalytic properties, attributed to its favorable interaction with reactants and high adsorption–desorption reaction kinetics during water catalysis.^[3]^ Molybdenum (Mo) may modify the electronic structure of the material, thereby enhancing the adsorption of reaction intermediates at active sites and improving HER/OER performance.^[4]^ Additionally, boron (B) exhibits a multi-bonding nature and can induce stronger ionic interactions with transitional metal Fe and Mo atoms, which helps lower the overpotential and enhances the crystallographic structure of the electrode material.^[5]^ The incorporation of heteroatom atoms such as Ni, Co, Ru and Cr can serve as an effective strategy to enhance water-splitting performance by modulating the electronic structure and reducing the kinetic barriers of the HER/OER process.^[6–10]^ Among them, cobalt (Co) is a low-cost transition metal ($ 0.94/oz), significantly cheaper than noble metals like Pt ($ 988/oz), Ir ($ 4,000/oz) and Ru ($ 20/oz), making it a cost-effective choice for water-splitting electrocatalysts.^[11]^ The Co can effectively optimize HER/OER due to its suitable H⁺/OH⁻ adsorption capability, flexible valence states and high surface reactivity.^[12]^ In addition, Co does interact with hydrogen H-containing species due to surface adsorption, coordination bonding and hydride formation (Co-H) in catalytic reaction.^[13]^

The Co-doping process was systematically optimized by adjusting key reaction parameters to assess its influence on the structural characteristics and electrocatalytic performance of the FeMoB electrode. Specifically, the Co concentration played a critical role in enhancing catalytic activity. The Co-doping concentration was optimized within the range of 0.1 ~ 0.8 mmol in Figs. S12–13, while the 0.4 mmol sample electrode exhibited the lowest HER/OER overpotentials. Subsequently, the hydrothermal reaction duration (from 4 to 16 hours) and temperature (from 150 to 250 °C) were optimized, as shown in Figs. S14–17. Among the tested conditions, the sample synthesized at 8 hours and 180 °C exhibited the best electrocatalytic performances. After the hydrothermal synthesis, the Co/FeMoB catalysts were naturally dried at room temperature. The most promising samples were subsequently subjected to post-annealing treatment in a rapid thermal processing (RTP) chamber under a high-vacuum environment of 1 × 10^-4^ torr. The annealing duration was varied between 15 and 120 minutes at 100 °C, with the 30-minute sample demonstrating the best HER/OER performance in the 3-E system as shown in Figs. S18–19. Additionally, the annealing temperature was adjusted from 100 to 400 °C, and the Co-doped FeMoB electrode treated at 300 °C was identified as the optimal condition based on its superior HER/OER performance in Figs S20 and 3(a–b). In summary, Co-doped FeMoB MPs were successfully synthesized while the Co incorporation significantly enhanced the intrinsic catalytic activity, electrochemically active surface area, electrical conductivity and long-term stability of the catalysts. Moreover, the combined hydrothermal and annealing processes proved to be not only effective but also scalable and cost-efficient, offering practical advantages such as the simultaneous fabrication of multiple samples and large-area electrodes.

**S-1.4. Physical Characterization**

The morphology of Co/FeMoB micro-petal electrodes was characterized by scanning electron microscopy (SEM, COXEM CX-200, South Korea). The elemental and compositional makeup was examined by employing an energy-dispersive X-ray spectrometer (EDS) system, Thermo Fisher Scientific, USA. Raman spectra were obtained using the NOST Raman system (Nostoptiks, South Korea), equipped with a 532 nm laser, a spectrograph (ANDOR SR-500, UK) and a charge-coupled device (CCD) detector. The transmission electron microscopy (TEM) system, (JEM-2100F, Japan) was utilized to investigate the atomic planes and crystal phases of Co/FeMoB MP electrodes. To verify the reproducibility and consistency of the interplanar spacing data, the high-resolution TEM (HR-TEM) measurements were conducted on multiple regions of the sample electrode. The phase composition and crystalline planes of sample MP electrodes were identified by X-ray diffraction (XRD, Bruker, USA) using Cu Kα radiation with λ = 1.5406 Å at a scanning parameter of 2º/min. The chemical state and electronic structure of Co/FeMoB MP electrodes were characterized using X-ray photoelectron spectroscopy (XPS) technique, Thermo Fisher Scientific, USA, with an X-ray spot size of approximately 10 µm at 1.5 kV, under a vacuum of <10^-8^ Torr.

**S-1.5.** **Electrochemical Characterization**

Electrochemical workstation (Wizmac, South Korea) was employed to perform electrochemical measurements using a three-electrode (3-E) configuration at room temperature. In this setup, the Co/FeMoB MP served as the working electrode, graphite acted as the counter electrode and Ag/AgCl functioned as the reference electrode. All linear sweep voltammetry (LSV) potentials are transformed to the reversible hydrogen electrode (RHE) scale. For LSV analysis, the hydrogen evolution reaction (HER) window ranged from – 0.6 to 0.2 V vs. RHE, while the potential window for the oxygen evolution reaction (OER) was set between 1.2 and 2.2 V vs. RHE. The HER/OER LSV measurements of Co/FeMoB were compared with benchmark electrodes (Pt/C for HER and RuO_2_ for OER) in different pH solutions. The pH conditions included alkaline 1 M KOH (pH: ~14), acidic 0.5 M H_2_SO_4_ (pH: ~0) and neutral 1 M PBS (pH: ~7.4). The HER/OER LSV scan rates were varied between the general range of 2 ~ 10 mV/s to observe the different scan-rate effects on the performance and Tafel slopes in 1 M KOH in Fig. S8. The Randles-Sevcik equation describes the functional correlation of peak current and scan rate in a diffusion-controlled electrochemical redox process. Response current, $i_{P}=0.446nFAC^{o}\left( \frac{nFvD_{o}}{RT} \right)^{1/2}$ (1)

In Randles-Sevcik equation (1), the *n* is the electron number transfers in the HER/OER redox process, active surface area of the electrode is defined with *A* (*cm^2^*), the Faradaic constant is *F* (*C mol^−1^*), the diffusion coefficient of analyte is *D_o_* (*cm^2^s^−1^*), the analyte concentration is *C_o_* (*mol cm^−3^*), the gas constant is *R* (*JK^−1^mol^−1^*) and *T* (*K*) is the temperature. In Eq. (1), the peak current (*i_P_)* is linearly increased with the square root (***√***) of the applied scan rate *v* (*mV/s*) as the diffusion layer can get smaller with increased scan rate. Thus, along with higher scan rates, increased overpotentials were observed as seen in Figs. 8(a–b). The Tafel slope was determined to evaluate the reaction kinetics from the linear region of the LSV polarization curves according to the Tafel equation ^[14]^: *η = a + b log|j|* (*η:* overpotential, *a:* intercept related to the [exchange current density](https://www.google.com/search?cs=0&sca_esv=7fc613d9cd9ef286&sxsrf=AE3TifP4G6BcuPO0HJcGYzFkzVK9v8yUxA%3A1756824508118&q=exchange+current+density&sa=X&ved=2ahUKEwj2jM-lqbqPAxVcdfUHHaLgJ5sQxccNegQIHhAB&mstk=AUtExfDNvTZPEwO4stWa8Ne7gsW4mvAFWKpsxgewEB_B3duvMVD0H9IyZfKL46J6N7YceNftyWGu1eqBHLbgdIwFoENOvnypIkYIAL02gP-G6OxqmOeEFsYpX1vUhp9OZlE4lYMImPfeCzGH8Wyoj6aj4YESF5ZiYt7-Zcz62FluYViylHarhBtEWoAAREkeF4tPaCVLDLuzRIwkWeUE1MOZig9MbD9qoInMXbIxYvxEA5Ha4cH5RJVEbr1o3_3mIG7EeXB-rf3QCsLHjOAq66qItf_M&csui=3) *(j₀)* and equilibrium potential*, b:* Tafel slope and *j*: current density). The Tafel slope was extracted with careful consideration of the linear region of LSV curves. The Tafel slope and slope values are shown in Figs. 8(a-1–b-1). The 5 mV/s demonstrated the highest LSV and lowest slope values in both OER/HER cases. On the other hand, the increased scan rate over 5 mV/s showed decreased performance, likely due to the increased mass diffusion resulting in the increased diffusion layer thickness and uncontrollable catalytic reactions. Generally, LSV characterization is commonly adapted at ~5 mV/s a scan rate in different electrolytes. All the electrochemical LSV measurements were plotted directly as recorded from the experimental data without iR drop correction. In general, the iR compensation can enhance the performance of LSV curves by eliminating the effect of solution resistance.^[15]^ However, the iR compensation was not adopted to ensure that the obtained electrochemical performance reflects the intrinsic catalytic behavior of the catalysts under practical experimental conditions.^[16]^ During gas-evolving reactions (e.g., H_2_/O_2_ generation), the uncompensated resistance can fluctuate due to continuous bubble formation which can make any fixed iR compensation value inaccurate, and iR compensation may also introduce artifacts/overestimation of catalytic activity if not carefully controlled. However, the samples were analyzed using a fixed cell setup. All the electrochemical measurements were carried out under stable and identical operational conditions for consistent electrochemical performance as much as possible to minimize experimental errors.

Electrochemical impedance spectroscopy (EIS) was conducted at a fixed current density of 15 mA/cm^2^ to evaluate the charge transfer resistance, spanning a frequency range (100 kHz ~ 0.1 Hz) with a 5-mV alternating current (AC) signal. In this work, the Randles equivalent circuit model was employed to analyze the EIS data, as it provides a simple and widely accepted representation for the HER/OER system.^[17]^ In the Randles equivalent circuit, R_s_, R_ct_ and CPE correspond to the solution resistance, charge-transfer resistance and constant phase element, respectively. The CPE is introduced to the model to represent the double-layer capacitance (C_dl_) of solid electrode under real operating conditions ^[18]^. The EIS voltage was varied to analyze its result depending on the charge transfer resistance (R_ct_) as seen in Fig. S9. The EIS responses can vary depending on the applied voltage; for instance, a lower R_ct_ value is typically observed at higher voltages according to the Butler–Volmer equation. Thus, at a fixed applied voltage around the catalytic turnover region, a well-performing electrode generally exhibits a lower R_ct_ value. Therefore, the EIS was conducted at the voltages associated with a constant current density of 15 mA/cm², considering the dependence of R_ct_ values on the applied potential to assess the general activity trend and corresponding charge transfer resistance (R_ct_).

Cyclic voltammetry (CV) was performed to determine the double-layer capacitance (C_dl_) along with estimate electrochemical surface area (ECSA) at scan rates ranging from 40 ~ 180 mV/s. The measurements were taken in the non-faradaic region: 0.1 – 0.3 V vs. RHE for HER and 1.04 – 1.14 V vs. RHE for OER. The C_dl_ values were adapted from the slope values of the linear relationship of the average current density (J = (Ja – Jc)/2) versus scan rate. The ECSA value can be calculated following the equation, ECSA = $\frac{C_{dl}}{C_{s}} \times Sg$, where the specific capacitance C_s_ = 0.04 mF/cm^2^, was generally taken for the flat electrode from the previous work. The exposed geometric surface area of the working electrode (Co/FeMoB) was $Sg$ = 0.5 cm (length) × 0.5 cm (width) = 0.25 cm^2^. The electrochemical surface area (ECSA) of each electrode was estimated based on the C_dl_ value of the catalytic surface. The ECSA can be a key parameter to evaluate intrinsic activity; however, it may not accurately reflect the true number of active sites, particularly in electrodeposited systems. Nevertheless, ECSA serves as a useful comparative metric for evaluating relative activity between samples, though it can vary depending on measurement conditions. To evaluate the intrinsic catalytic activity before/after Co incorporation, the LSV curves were normalized to the ECSA values.^[19]^ More details related to [turnover frequency](https://www.sciencedirect.com/topics/chemistry/turnover-frequency) (TOF) and faradaic efficiency (FE) measurements can be found in SI sections S-1.7 & 1.9.

Chronoamperometry (CA), chronopotentiometry (CP) and post-cyclic voltammetry (CV) measurements were conducted to assess the stability and repeatability of the Co/FeMoB MP electrocatalysts. The CA tests were conducted at various applied potentials, and linear sweep voltammetry (LSV) was recorded after 2,000 CV cycles over a 12-hour period to assess the robustness and reproducibility of the HER/OER process. Long-term CP-stability measurements were performed at high current densities of 300, 600 and 1,000 mA/cm² under different operational conditions. For two-electrode (2-E) overall water electrolysis (OWE) systems, three configurations were designed based on the choice of cathode_(−)_ and anode_(+)_: (i) bifunctional: Co/FeMoB_(−)_ // Co/FeMoB_(+)_, (ii) hybrid: Pt/C_(−)_ // Co/FeMoB_(+)_ and (iii) benchmark: Pt/C_(−)_ // RuO_2(+)_. The 2-E OWE was performed in electrolytes of varying pH including alkaline (KOH, 1 M), acidic (H_2_SO_4_, 0.5 M) and neutral (PBS, 1 M) solutions. The OWE performance was assessed in 1 M at 25 °C and 6 M KOH electrolytes at an elevated temperature of 60 °C, reaching current densities up to 2,000 mA/cm^2^ for industrial-scale operation. All the 2-E electrochemical activity for the bifunctional and hybrid systems are summarized in Tables S4–S7.

**S-1.6. Benchmark HER/OER Electrodes**

As benchmark electrodes, Pt/C and RuO_2_ were utilized for the HER and OER, respectively. The benchmark electrodes were fabricated on bare nickel foam (NF) substrate using a soaking approach as described in previous literatures^[10,20]^. The HER benchmark Pt/C was fabricated using a soaking approach. To prepare the Pt/C catalyst, 20 mg of Pt/C powder and 60 µL of 5% Nafion (117 solutions, Aldrich) solution were dispersed in a 10 mL mixture of ethanol and deionized (DI) water (1:1 ratio). Then, the precursor solution was ultrasonicated for 30 minutes to achieve a clear mixture, after which a clean NF substrate (1 × 1 cm^2^) was soaked in the solution for 30 minutes. The fabricated Pt/C electrode was dried at ambient conditions before use. The morphological, elemental and electrochemical characterizations of the Pt/C electrode are provided in Fig. S10. The benchmark Pt/C electrode exhibited overpotentials of 133 and 220 mV at 50 and 600 mA/cm^2^ in Figs. S10(c–d). The fabrication of the OER benchmark RuO_2_ was carried out with the same soaking approach. To synthesize the RuO_2_ catalyst, 40 mg of RuO_2_ powder and 60 µL of 5 wt.% Nafion were mixed into 10 mL of a 1:1 ethanol and DI water solution, followed by an ultrasonication for 30 minutes. Then, the NF substrate was soaked in the precursor solution for 30 minutes and air-dried prior to use. The morphological, elemental and electrochemical characterizations of the RuO_2_ electrode are provided in Fig. S11. The RuO_2_ benchmark demonstrated overpotentials of 386 and 652 mV at 200 and 600 mA/cm^2^ in Figs. S11(c–d).

**S-1.7. Turnover Frequency (TOF) Calculation**

TOF (turnover frequency) serves as a reliable descriptor of a catalyst’s intrinsic activity which can define the number of reaction events (reactant molecules converted to products) occurring at per catalytic active site per unit time^[21]^. The catalytic ability to produce hydrogen (H_2_) and oxygen (O_2_) molecules along with metallic active sites of the catalyst can be determined by the TOF calculation.^[22–24]^ The TOF values of HER/OER can be calculated from the following equation below^[16]^:

TOF = $\frac{\frac{Total number of H_{2}\mathrm{or}O_{2} \mathrm{turnover}}{geometric area {(cm}^{2})} \times Current density}{\frac{Number of active sites}{geometric area {(cm}^{2})}}$ (2)

Total theoretical H_2_ turnover:

*=* $\left( j\frac{\mathrm{mA}}{\mathrm{cm}^{2}} \right)\left( \frac{1\frac{C}{s}}{1000 mA} \right)\left( \frac{1 \frac{\mathrm{mol}}{e}}{96485 C} \right)\left( \frac{1 mol H_{2}}{2 \frac{\mathrm{mol}}{e}} \right)\left( \frac{6.022 \times{10}^{23}\mathrm{mol}H_{2}}{1 mol H_{2}} \right)$ *=* $3.12 \times{10}^{15} \left( \frac{\frac{H_{2}}{s}}{{cm}^{2}} \right) per (\frac{mA}{{cm}^{2}})$

Total theoretical O_2_ turnover:

= $\left( j\frac{\mathrm{mA}}{\mathrm{cm}^{2}} \right)\left( \frac{1\frac{C}{s}}{1000 mA} \right)\left( \frac{1 \frac{\mathrm{mol}}{e}}{96485 C} \right)\left( \frac{1 mol O_{2}}{4 \frac{\mathrm{mol}}{e}} \right)\left( \frac{6.022 \times{10}^{23}\mathrm{mol}O_{2}}{1 mol O_{2}} \right)$ *=* $1.56 \times{10}^{15} \left( \frac{\frac{O_{2}}{s}}{{cm}^{2}} \right) per (\frac{mA}{{cm}^{2}})$

The number of catalytic active sites was determined based on the presence of metallic species, all of which are considered active sites directly responsible for catalyzing HER and OER reactions. For the Co/FeMoB electrode, the Co, Fe and Mo metals are the active species.

**Active sites of Co/FeMoB MP electrocatalyst:**

$=\frac{EDS \%}{100} \times amonut of loading per area\times Molecular mass\times\left( \frac{Avogadro Number}{Per concentration (mmol)} \right)$ (3)

$=\frac{5.47}{100} \times0.30\frac{\mathrm{mg}}{\mathrm{cm}^{2}}\times\frac{1 mmol}{58.93 mg} \times\frac{49.70}{100} \times0.30\frac{\mathrm{mg}}{\mathrm{cm}^{2}}\times\frac{1 mmol}{55.84 mg}\times\frac{41.43}{100} \times0.30\frac{\mathrm{mg}}{\mathrm{cm}^{2}}\times\frac{1 mmol}{95.95 mg}\times\left( \frac{6.022 \times{10}^{23}\mathrm{mmol}}{3 mmol} \right)$

${=1.94\times10}^{17} Active sites.{cm}^{-2}$ *per mmole*

The average weighting percentages of Co, Fe and Mo were 4.99, 49.79 and 41.50 (wt.%) from the EDS analysis of the post-annealing control set in Fig. S21. The amount of loading materials was 0.28 mg/cm^2^ in the electrode (grown sample: 0.0464 g and bare Ni: 0.0434 g). The molecular mass of Co, Fe and Mo are 58.93, 55.84, and 95.95 gm used for the calculation of metallic active sites.

**HER TOF** = $\frac{3.12 \times{10}^{15}\times300}{{1.94 \times10}^{17}}$ ***=*** *4.83* *site^-1^s^-1^*

**OER TOF** = $\frac{1.56 \times{10}^{15}\times300}{{1.94 \times10}^{17}}$ *= 2.41 site^-1^s^-1^*

The HER/OER TOF of different Co/FeMoB samples of post-annealing temperature variation set can be calculated using Eq. (3). For the HER, Co/FeMoB electrodes annealed at 100, 200, 300, and 400 ºC delivered current densities of 283.6, 293.9 300 and 273.27 mA/cm^2^ at a constant overpotential of 160 mV in 1 M KOH; while for the OER, the same electrodes exhibited current densities of 253.9, 279.3, 300 and 232.5 mA/cm^2^ at a constant overpotential of 352 mV. The HER and OER TOF values for the best-performing Co/FeMoB electrodes annealed at 300 ºC were calculated as shown above, and the TOF values for the other electrodes were determined using the same method. The corresponding HER/OER TOF values are presented in Fig. 3(c).

**S-1.8. HER/OER Stability and Different pH Activity**

The steady state chronoamperometry (CA) measurements of the Co/FeMoB catalyst demonstrated stable HER/OER current responses at various applied potentials in 1 M KOH as shown in Fig. S30. The consistent CA profiles indicate the continuous formation and release of gas bubbles without obstructing the catalytic surface reactions. In addition, the minor current variation (%) between CA and LSV responses across different potentials, confirms efficient bubble detachment and suggests no significant performance degradation.^[25]^ The HER/OER LSV curves of the Co/FeMoB catalyst recorded after 2,000 CV cycles in 1 M KOH in Fig. S31 exhibited negligible change, demonstrating excellent electrochemical durability. In addition, the Co/FeMoB MP catalyst demonstrated excellent double-step long-term chronoamperometric (CP) stability for both HER/OER, maintaining continuous H_2_/O_2_ production at 300/500 mA/cm^2^ current densities in 1 M KOH for 50 hours as shown in Fig. S32. Finally, the LSV performance of Co/FeMoB was compared with the bare NF substrate as seen in Fig. S33, which demonstrated that the substrate contributed only marginally to the overall catalytic improvement despite having some intrinsic catalytic activity. The Co/FeMoB MP exhibited slight linearity in the HER polarization curve as generally observed in previous literatures ^[26–28]^, which can be attributed to a minor improvement in interfacial resistance between the electrode and the electrolyte. This small increase in resistance may be resulted from the formation and accumulation of hydrogen gas bubbles on the electrode surface during the H_2_ evolution reaction. Figure S34 demonstrates the HER/OER performance of the Co-doped FeMoB multifunctional catalyst with benchmark electrodes (Pt/C for HER and RuO_2_ for OER) under various pH conditions. The tests were conducted in 1 M KOH (alkaline, pH ~14), 0.5 M H_2_SO_4_ (acidic, pH ~0) and 1 M PBS (neutral, pH ~7.4) to evaluate performance under a wide range of pH environments. A detailed discussion related to preparation and characterization of the benchmark electrodes is provided in SI text S-1.6. However, the Co-doped catalysts exhibited significantly superior OER activity and comparable HER performance to the benchmarks under all pH conditions. The Co-doped multifunctional electrode exhibited higher HER overpotentials of 295, 328 and 363 mV at a high current density of 600 mA/cm^2^ in KOH (1 M), H_2_SO_4_ (0.5 M) and PBS (1 M) over the benchmark Pt/C performance, respectively as shown in Figs. S34(a–c). For the OER, the Co/FeMoB demonstrated significantly lower overpotentials of 453, 496 and 566 mV at 600 mA/cm^2^ in the same respective electrolytes compared to the benchmark RuO_2_, which exhibited higher overpotentials of 652, 868 and 1176 mV as seen in Figs. 34(d–f). The significantly lower overpotentials of 199, 372 and 610 mV as opposed to the respective benchmark values, are crucial for achieving high-performance overall water electrolysis. However, both the Co-doped and benchmark catalysts exhibited the highest performance in alkaline media (KOH + H_2_O), attributed to less corrosive nature of the solution and higher ionic conductivity. That is why alkaline electrolytes are predominantly used in commercial systems, owing to their advantages such as cost-effectiveness, lower corrosivity and high hydrogen production rates. The abundant presence of K⁺ and OH⁻ ions in alkaline solutions enhances surface adsorption and promotes rapid ion mobility, without adversely affecting the stability of the active catalytic species.^[29]^ Meanwhile, the HER/OER overpotential values increased under harsh acidic environments (H_2_SO_4_ + KOH) primarily due to the high concentration of protons (H⁺), which can induce minor corrosion of the active species and pose challenges for TM-based catalysts.^[30]^ Despite the harsh acidic conditions, the Co-doped electrocatalyst outperformed the benchmark catalyst attributed to its robust surface structure that effectively resists corrosion, slows down active site agglomeration and maintains long-term catalytic stability.^[31,32]^ Further, the HER/OER electrodes exhibited relatively higher overpotentials in neutral media (PBS + KOH) compared to acidic and alkaline media. This behavior is likely caused by poor ionic conductivity, significant internal resistance and enhanced heat buildup characteristics of neutral electrolytes.^[33]^ The pH of 1 M PBS (~7.4) closely resembles that of natural seawater, making it a relevant medium for practical electrolysis applications. The Co-doped multifunctional FeMoB electrode demonstrated efficient HER/OER performance under neutral conditions, indicating its potential for direct natural H_2_O electrolysis. This enhanced activity can be attributed to a combination of improved mass transport, enlarged electrochemically active surface area and high intrinsic catalytic activity. However, the different pH TOF calculations of Co/FeMoB MP (300 ºC-annealed) in different pH conditions were performed at high current densities using the same methodology at fixed HER/OER overpotentials as previously described in SI text S-1.7. The corresponding TOF values, derived from the LSV curves shown in Fig. S34 for alkaline, acidic, and neutral media, are presented in Fig. S35. Among the tested environments, the Co/FeMoB catalyst exhibited the highest TOF values of 4.83 and 2.41 site^-1^s^-1^ at fixed overpotentials of 160 mV (HER) and 352 mV (OER), respectively, in 1 M KOH. The overall catalytic activity followed the trend: alkaline > acidic > neutral, highlighting the superior performance of the electrode in KOH media. Finally, to examine the temperature-dependent catalytic behavior, the alkaline electrolyte (1 M KOH) was heated from room temperature (RT, 25 °C) to 85 °C as shown in Fig. S36. The catalytic HER/OER performance progressively improved with increasing temperature up to 65 °C, primarily due to accelerated reaction kinetics and enhanced charge-transfer efficiency at the electrode–electrolyte interface. The elevated temperatures can facilitate faster ion mobility and lower the activation energy barrier, leading to reduced overpotentials and higher current densities for both HER/OER.^[34,35]^ However, a slight decline in activity was observed at 85 °C, likely resulting from structural instability of the catalyst and increased electrolyte evaporation. These observations indicate that maintaining a moderately high electrolyte temperature can be an effective strategy for achieving efficient, large-scale commercial alkaline H_2_ production.

**S-1.9. Faradic Efficiency (FE) Measurement**

Faradaic efficiency (FE) is determined as a crucial metric that quantifies the ratio of the experimentally measured amount of hydrogen (H_2_) or oxygen (O_2_) produced to the theoretically calculated amount during electrolysis.^[36]^ The FE of the Co/FeMoB electocatalyst can be calculated as shown in Eq. (6).^[37,38]^

$$Faradaic efficiecy=\frac{Practically produce mol of H_{2}/ O_{2} gas}{Theoretically Produce mol of H_{2}/ O_{2} gas}\times100 (4)$$

The theoretical value of H_2_ or O_2_ can be calculated by using Faraday’s law as below:

$$n=\frac{I\times t}{z\times F} (5)$$

where ‘*n*’ defines the amount of theoretically produced product (H_2_ or O_2_) in mole, *‘I’* is the current density in amperes (A), *‘t*’ is the time/duration in seconds (s) and *‘z’* is the number of charge/electron transferred ‘*z*’ = 2 for H_2_ or ‘*z*’ = 4 for O_2_ and Faraday constant ‘*F*’ = 96,485 C mol^-1^. The theoretical values of H_2_ and O_2_ generation can be calculated at 300 mA/cm^2^ current density for 15 ~ 60 minutes duration.

**The theoretical values:**

H_2_ generation: n = 1.39, 2.79, 4.19 and 5.59 mmol respectively during 15, 30, 45 and 60 minutes.

O_2_ generation: n = 0.699, 1.39, 2.09 and 2.79 mmol respectively during 15, 30, 45 and 60 minutes.

The generated gas was collected using the water-gas displacement method. The numbers of H_2_ or O_2_ molecules can be calculated using the following ideal gas eq.:

$$PV=nRT (6)$$

where, the volume of collected gas *‘V’* is liters (L), the temperature *‘T’* in kelvin (K), the ideal gas constant value *‘R’* is 0.0821 L.atm.mol^-1^K^-1^ and the atmospheric pressure *‘P’* is ~1 atm. This unit for ‘*R*’ offers a convenient relationship between experimentally measured gas volume (in liters, L), simplifies calculations and aligns well with common volume units.

**The number of H_2_ and O_2_ in water-gas displacement:**

The volume of H_2_ gas collected was 0.0311, 0.02385, 0.09688 and 0.131 L respectively for 15-, 30-, 45- and 60-minutes duration,

For 60 minutes, $\left( 1 atm \right) \left( 0.131 L \right)=n\left( 0.0821\frac{\mathrm{atm}}{\mathrm{mol}K} \right)\left( 298K \right)$

$n=$ 5.35 mmol

The volume of O_2_ gas collected was 0.0154, 0.313, 0.0474 and 0.0653 L respectively for 15, 30, 45 and 60 minutes.

For 60 minutes, $\left( 1 atm \right) \left( 0.0653 L \right)=n\left( 0.0821\frac{\mathrm{atm}}{\mathrm{mol}K} \right)\left( 298K \right)$

$n=$ 2.67 mmol

A similar process was followed to calculate other observation durations. The HER/OER Faradaic efficiencies (FE) of the best-performing Co/FeMoB electrodes are shown in Fig. 3(i), while the corresponding gas yields and a digital image of the water-gas displacement setup are provided in Fig. S38. The theoretical and practical values comparisons were presented in Figs. S39. The Co/FeMoB demonstrated HER/OER faradaic efficiency of 96.06 and 95.34 % in 1 M KOH for 60 min duration. The high Faradaic efficiency (FE) values of Co/FeMoB MP for H_2_ and O_2_ evolution indicate extremely low energy consumption, highlighting its suitability for real-world applications.

**S-1.10. Post-Stability Characterization of Co/FeMoB MP**

The post-stability physical, structural and electrochemical characterizations of Co-doped FeMoB were conducted after a 20-hour stability test at 1,000 mA/cm^2^ in 1 M KOH solution. The analyses included SEM, Raman spectroscopy, XRD, XPS and LSV for HER/OER and overall water-electrolysis (OWE) as shown in Figs. S58–64. In this work, the Co-doped FeMoB exhibited superior anodic activity, and all physical characterizations were performed on the anodic Co/FeMoB electrode. After the stability test, the Co/FeMoB electrode exhibited nearly similar micro-petal-like morphology with only minor surface corrosion observed in the SEM micrographs as shown in Fig. S58. This demonstrates the strong morphological stability of the Co-doped FeMoB MP. The observed surface roughness can be attributed to extensive bubble formation, surface oxidation, and the adsorption of oxygen-containing intermediates during high-current redox reactions.^[39]^ Further, EDS spectra analysis were employed to determine the compositional changes before/after the stability test as shown in Fig. S59. The pre- and post-stability electrodes exhibited distinct peaks corresponding to Co, Fe, Mo, B and O, confirming the presence of each element. This rational quantitative characterization of the contents revealed that the weight percentages of the predominant components (Co, Fe, Mo, and B) decreased slightly. However, the oxygen content increased from 2.23% to 3.77% after the stability test. These subtle variations suggest surface oxidation and partial structural reconstruction during electrochemical operation. Moreover, the comparative Raman spectroscopy analysis of Co/FeMoB electrodes is presented in Fig. S60. After the stability assessment, the Raman characteristic peaks at 282, 667, 819, 946 and 993 cm^-1^ retained their original positions but showed slight reductions in intensity, indicating the stable crystallographic structure of the electrode. Further, the post-stability and pre-stability of XRD diffraction patterens of Co/FeMoB MP is compared in Fig. S61. The post-stability XRD analysis showed that all characteristic diffraction peak positions were generally maintained; however, slight reductions in intensity and peak disappearance were also observed. Specifically, the peaks located at 33.03°, 38.50° and 58.99° gentally maintained their pre-stability positions with a slight intensity reduction, whereas the peaks at 62.65° and 72.96° completely disappeared. The collective changes in XRD and Raman spectra after the post-stability test of Co/FeMoB can be likely due to metallic phase transformation and; for example, as represented by the reaction: M–OH^*^ + OH⁻ → M–OOH + H_2_O + e⁻.^[40]^ The post-stability full-range XPS spectrum of Co/FeMoB MP is shown in Fig. S62. The survey scan confirms the presence of all active elements Co, Fe, Mo and B after the stability test. The core-level HR-XPS spectra of each element before and after the stability operation were carefully investigated, and the corresponding pre- and post-stability HR-XPS spectra are provided in Figs. 2(d–d-4) and S63. The overall analysis indicated that all the characteristic HR-XPS peaks decently maintained their pristine peak structures (shape/ratio) and positions, while the elemental peaks exhibited a reduction in intensity and the ionic/oxide states showed an increment in intensity. More specifically, the Fe 2p_1/2_ and Fe 2p_3/2_ elemental peaks exhibited slight reductions, while the Fe^2+^/Fe^3+^ peak intensity increased considerably. These changes can be attributed to the surface oxidation by Fe species and formation of iron oxides or oxyhydroxides such as Fe_2_O_3_, Fe_3_O_4_ or Fe–O–OH. Similarly, the peak intensity of Mo 3d_3/2_, Mo 3d_5/2_ and B 1s states were decreased and the Mo^4+^, Mo^6+^ and B_2_O_3_ states largely increased after the stability reaction. This observed transformation indicated the formation of mixed MoO_2_ and MoO_3_ phases likely due to the partial oxidation of Mo atoms, along with the development of B–O surface layers ^[41,42]^. These can act as a protective surface-oxide layer or synergistic surface phases, which can contribute to structural stability of the catalyst under oxidative conditions. Moreover, the Co 2p_1/2_ and Co 2p_3/2_ peaks also showed a slight reduction in intensity with maintaining their original features, whereas the ionic states of Co^2+^/Co^3+^ revealed an increase in intensity, likely attributed to surface reconstruction/structural reconfiguration by the active Co species. Comprehensively, the decrease in elemental states was likely due to surface passivation and increased rigidity caused by the adsorption of reaction intermediates ^[43]^. Besides that, the observed variation in oxidation states likely originates from partial surface oxidation and the generation of catalytically active metallic oxide/oxyhydroxide species (M–O, M–OH and M–OOH), together with oxidation-induced phase transitions of Fe, Mo and Co (Fe^2+^ → Fe^3+^, Mo^4+^ → Mo^6+^ and Co^2+^ → Co^3+^) ^[44,45]^. These decreases in elemental states and increases in oxidation states also support the EDS observations. Overall, the conversion of peak intensity from elemental to oxidized states provides insight into the oxidation process, surface reconstruction and formation of oxide layers during prolonged operation under high-current mutual redox reaction in alkaline environment ^[46,47]^. These “before-and-after” HR-XPS results clearly demonstrate that Fe and Co serve as the primary redox-active centers (Fe^2+^/Fe^3+^ and Co^2+^/Co^3+^) responsible for the OER/HER interfacial reactions, whereas Mo and B contribute to maintaining electronic balance and structural integrity. The coexistence of multiple elements can promote efficient charge redistribution and accelerate the redox cycling among the transitional metals and boron. Thus, the synergistic electron exchange within the Co–Fe–Mo–B network can stabilize the surface and facilitate continuous redox reactions, thereby enhancing the overall efficiency of water electrocatalysis. Finally, the post-stability electrochemical activity in KOH solution of Co-doped FeMoB is presented in Figs. S62. The HER, OER and OWE operation (CoFeMoB electrode used as cathode: HER and anode: OER) exhibited nearly the same LSV performance as before, indicating strong catalytic durability in water-splitting system. Therefore, the excellent stability of the Co/FeMoB MP electrocatalyst can be attributed to strong interactions among active components, protective layer formation and improved corrosion resistance.

**S-2. Optimization of Co/FeMoB catalyst**

**S-2.1. Substate and other necessities**

**S-2.1.1. Schematic of FeMoB MP fabrication route**

**S-2.1.2. Bare nickel foam (NF) characterization**

**S-2.1.3. Base template FeMoB: SEM & EDS spectrum**

**S-2.1.4. Base template FeMoB: EDS mapping & line profile**

**S-2.1.5. Base template FeMoB: Raman & XRD**

**S-2.1.6. Base template FeMoB: Electrochemical LSV**

**S-2.1.7. Scan rate control on Co/FeMoB MP**

**S-2.1.8. EIS voltage control on Co/FeMoB MP**

**S-2.1.9. HER benchmark Pt/C electrode**

**S-2.1.10. OER benchmark RuO_2_ electrode**

**S-2.2. Co-doping optimization parameter and system**

**S-2.2.1 Hydrothermal: Co concentration**

**S-2.2.2. Hydrothermal: Reaction duration**

**S-2.2.3. Hydrothermal: Reaction temperature**

**S-2.2.4. Hydrothermal: Annealing duration**

**S-2.2.5. Hydrothermal: Annealing temperature**

**S-2.2.6. Annealing temperature: EDS**

**S-2.2.7. Annealing temperature: CV**

**S-2.2.8. Annealing temperature: C_dl_ valuesS-2.1.1. Schematic of Co/FeMoB MP fabrication route**

**
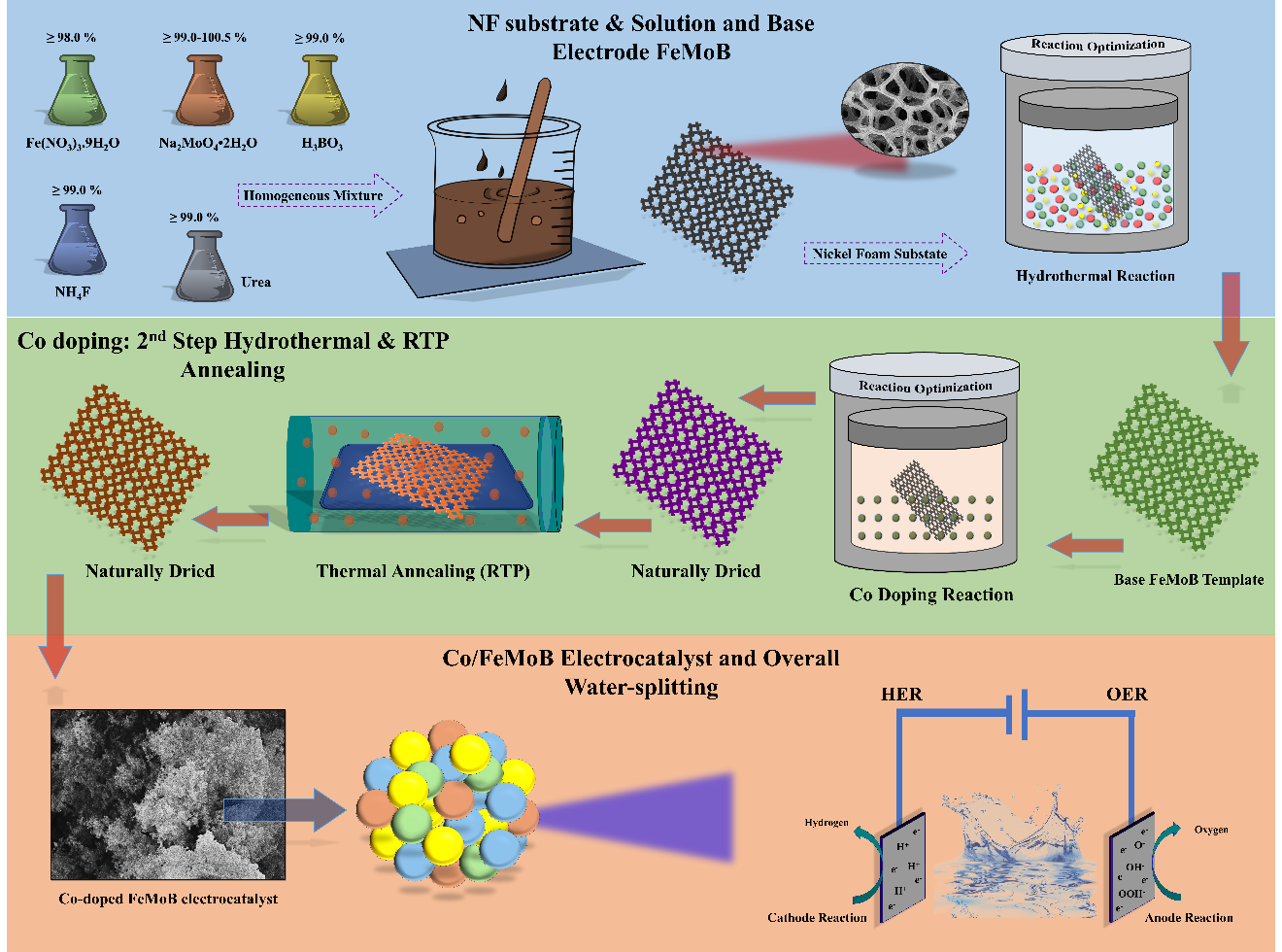
**

**Figure S1.** Schematic illustration of Co-doped FeMoB micro-patel (MP) electrocatalyst fabrication using a double-step hydrothermal reaction and thermal annealing treatment. The Co/FeMoB electrode works as a highly efficient and stable electrocatalyst for overall water electrocatalysis. A more detailed discussion related to fabrication of Co/FeMoB MP electrocatalyst can be found in the supplementary information (SI) text (S-1.2–1.3).

**S-2.1.2. Bare nickel foam (NF) characterization**

**
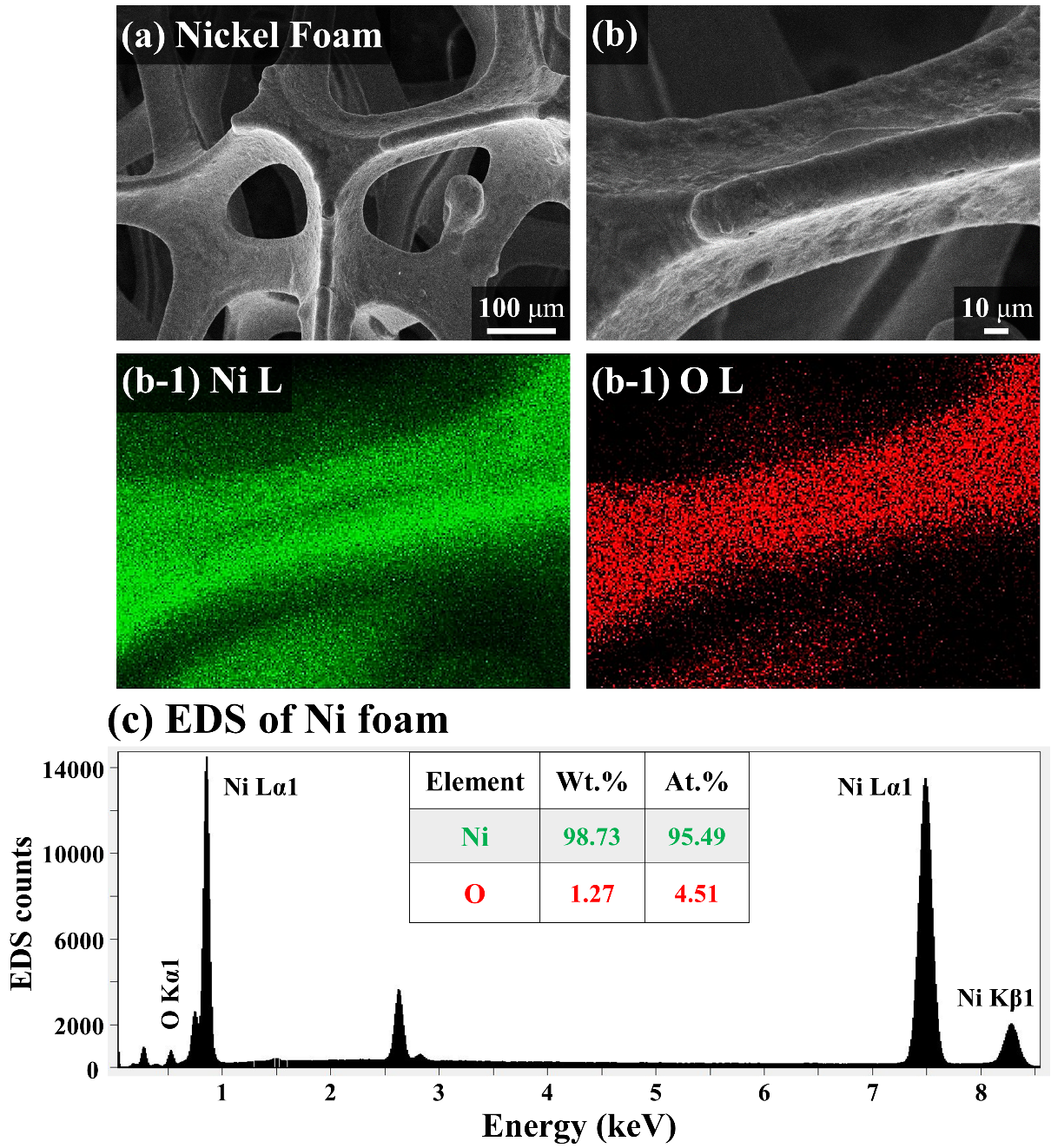
**

**Figure S2.** Physical analysis of 3-D porous bare nickel foam (NF) substrate. (a–b) SEM micrographs of NF and related enlarged view. (b-1–b-2) EDS phase mapping of Ni and O elements. (c) EDS spectrum of NF with weight (Wt.) and atomic (At.) percentages.

**S-2.1.2. Bare nickel foam (NF) characterization**

**
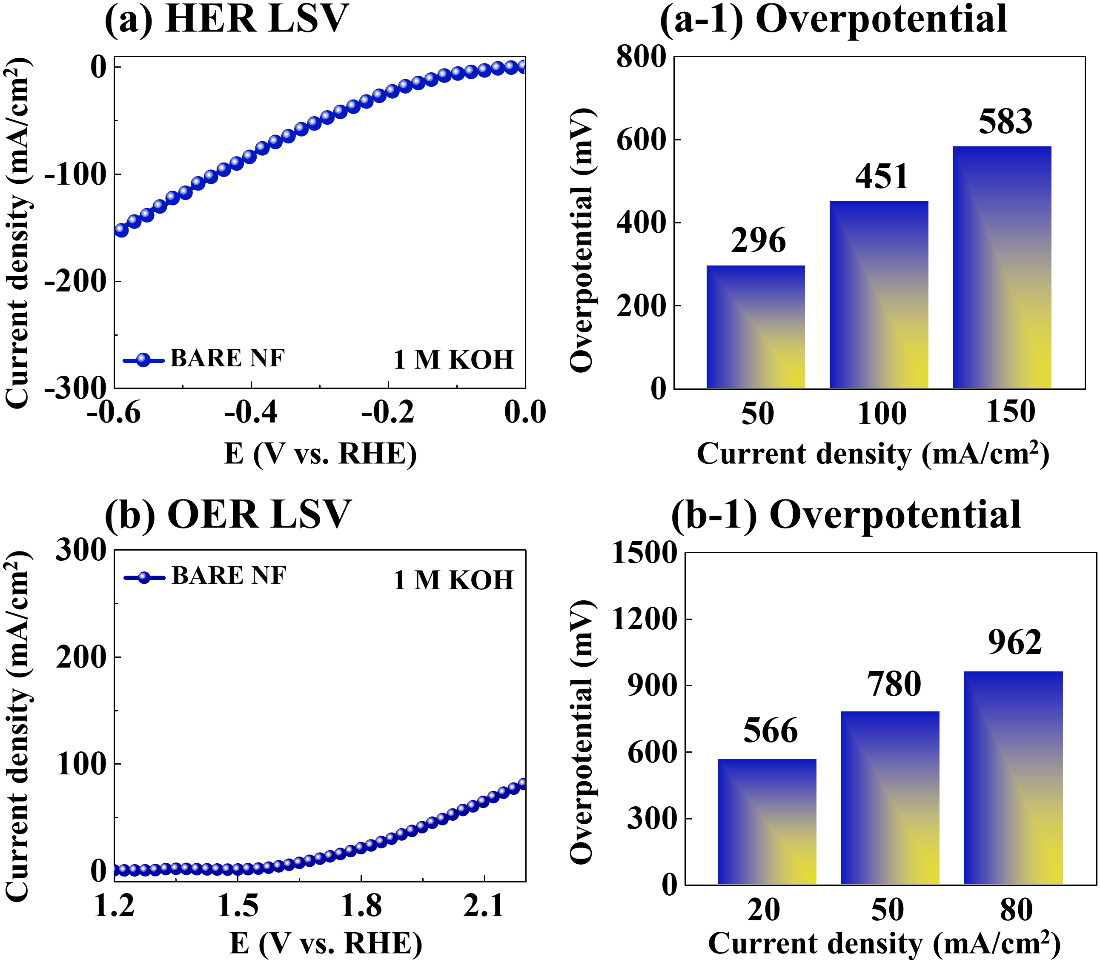
**

**Figure S3.** Electrochemical analysis NF substrate. (a–b) HER/OER performances of NF in 1 m KOH. (a-1–b-1) Corresponding overpotential bar graphs of HER/OER.

**S-2.1.3. Base template FeMoB: SEM & EDS spectrum**

**
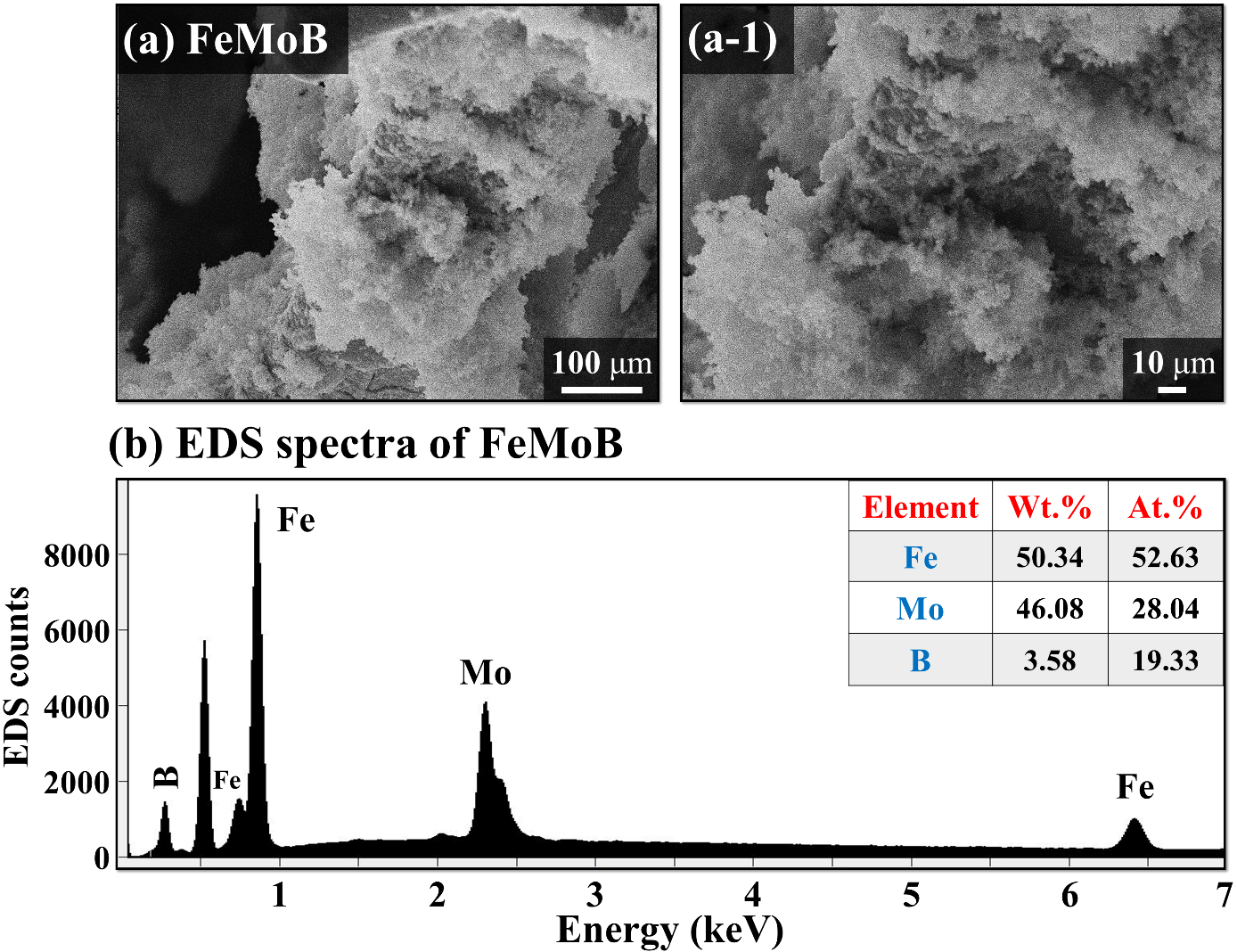
**

**Figure S4.** Base electrode FeMoB micro-cloud cluster (MCC) template. (a–a-1) Morphological SEM micrograph of FeMoB MCC. (b) EDS spectrum of FeMoB with Wt. and At. percentages.

**S-2.1.4. Base template FeMoB: EDS mapping & line profile**

**
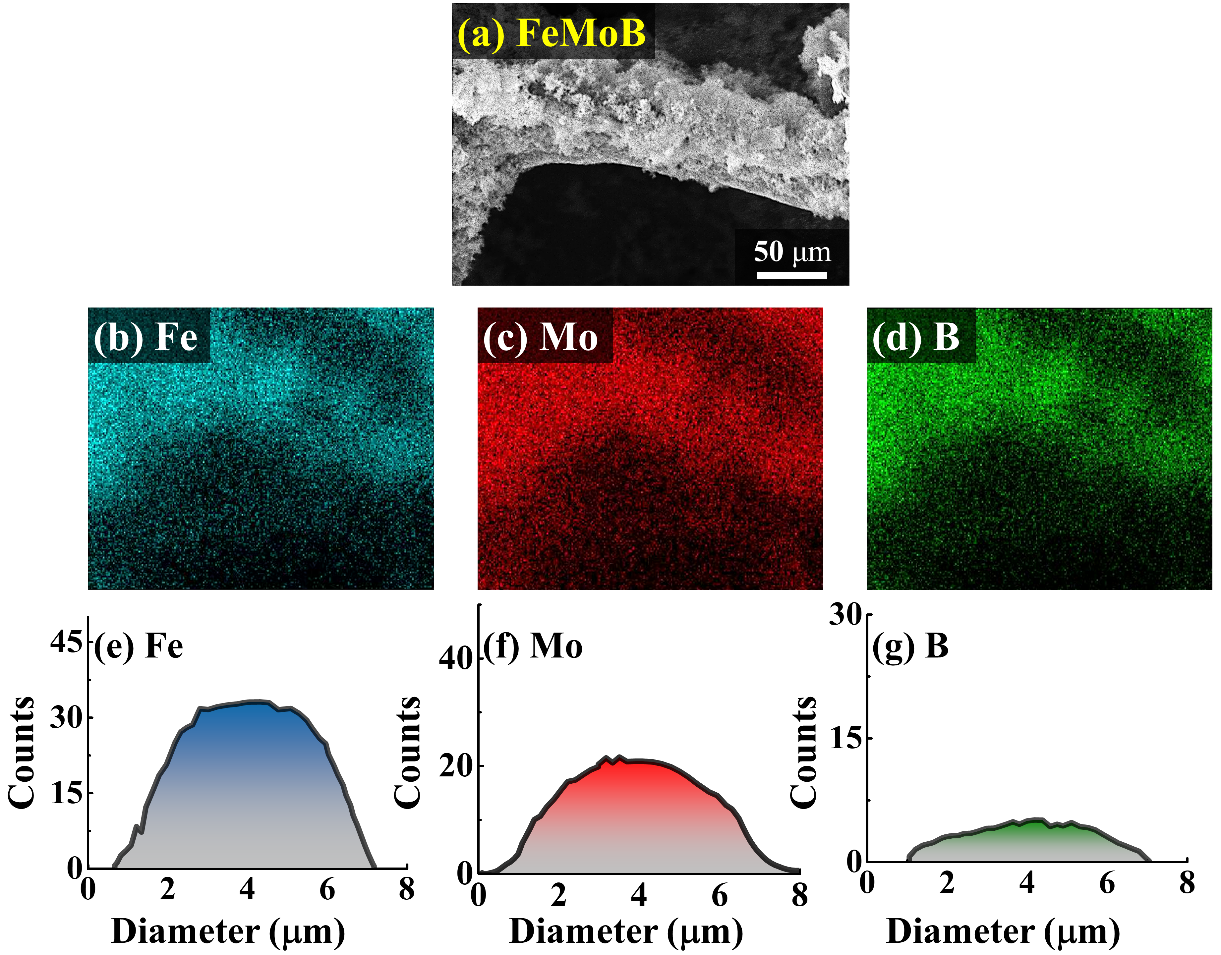
**

**Figure S5.** Morphological and elemental analysis for the FeMoB base template. (a) SEM micrograph of MCC electrode. (b–c) EDS elemental phase mapping of FeMoB. (e–g) EDS line profiles for Fe, Mo and B.**S-2.1.5. Base template FeMoB base template: Raman & XRD**

**
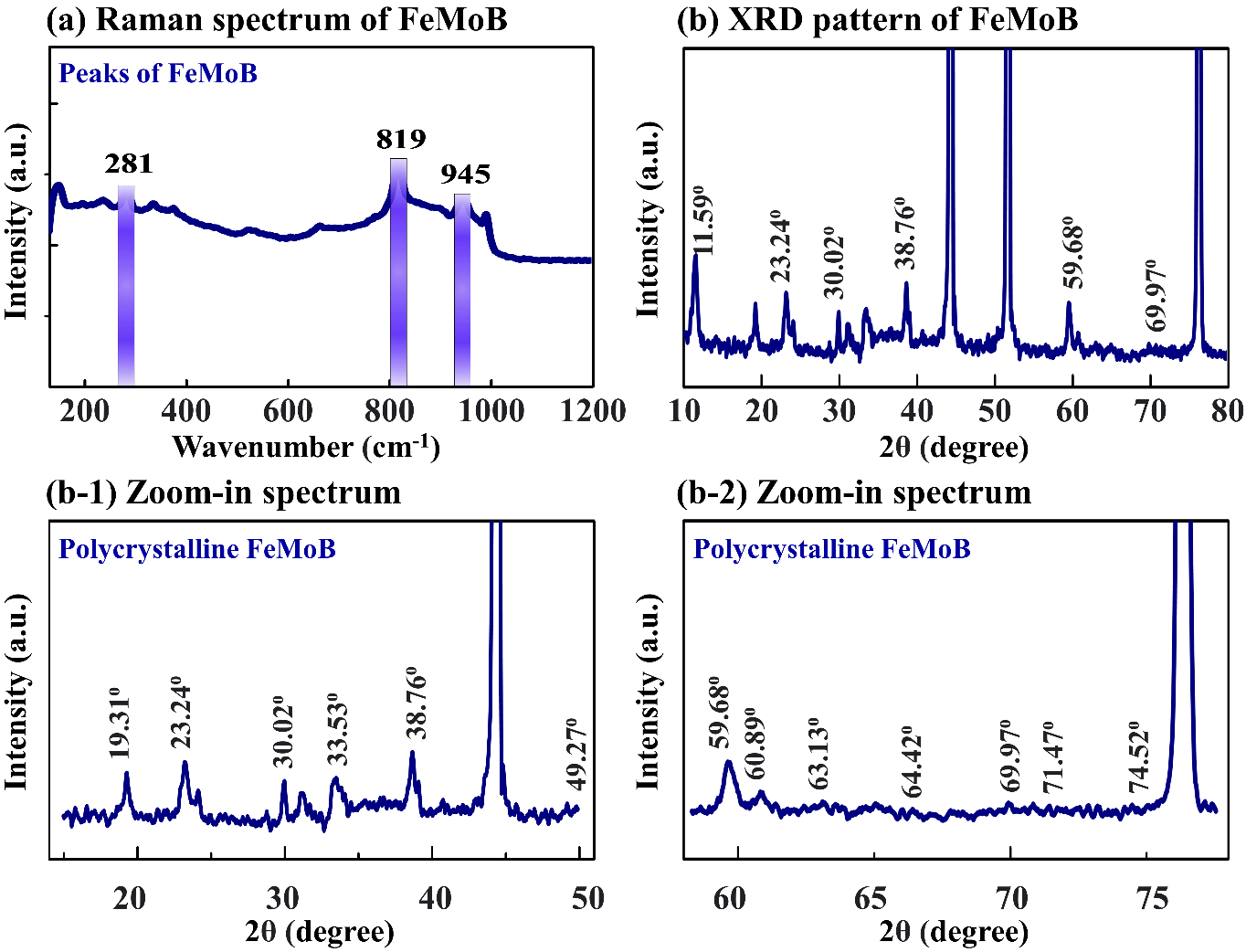
**

**Figure S6.** Raman spectroscopy on FeMoB base electrode. (a) Raman signal of FeMoB. X-ray diffraction (XRD) patterns of base FeMoB. (b) Full range XRD pattern of FeMoB in between 10 ~ 80º region. (b-1–b-2) Zoom-in spectrum view. **S-2.1.6. Base template FeMoB: Electrochemical LSV**

**
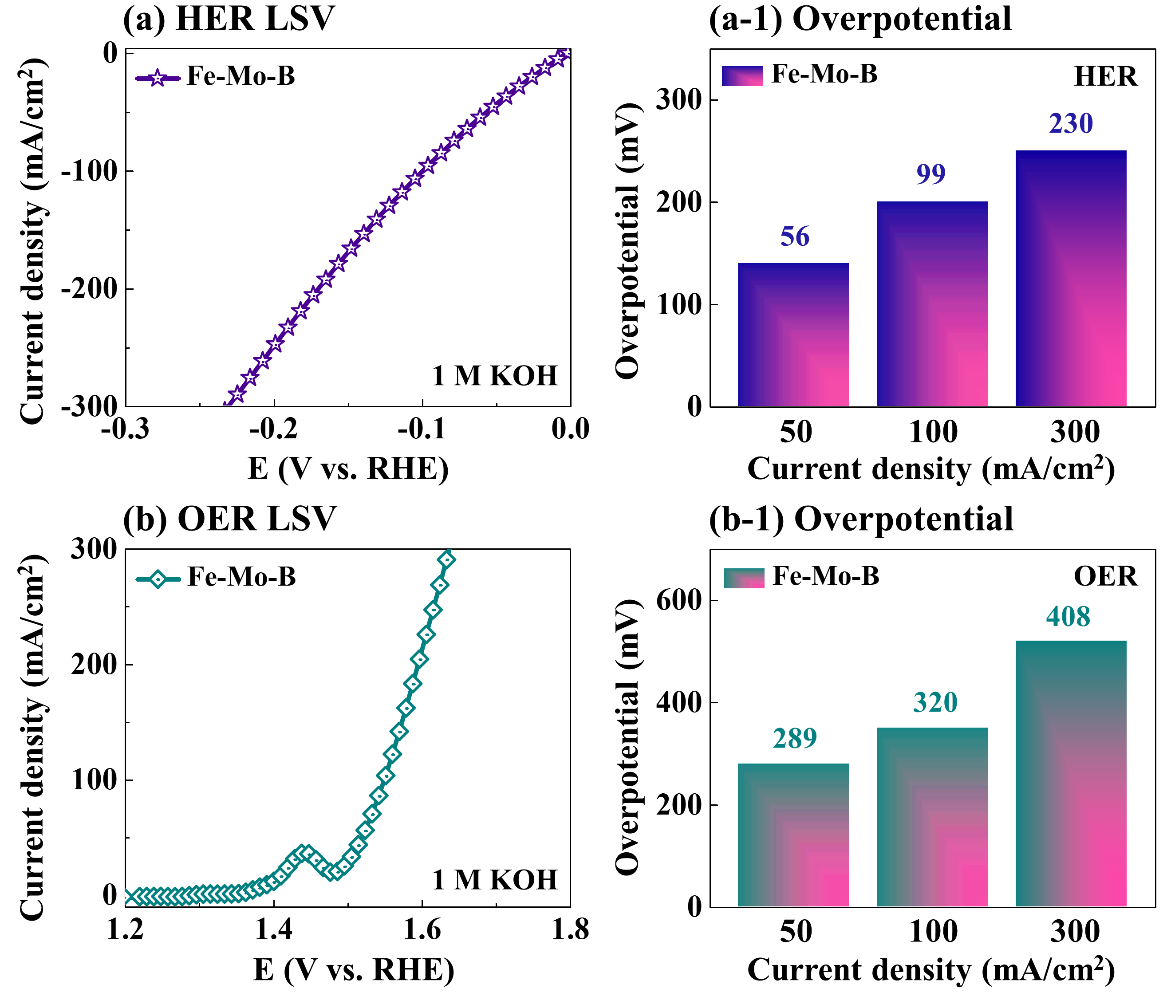
**

**Figure S7.** Electrochemical analysis FeMoB electrode. (a–b) HER/OER performances of FeMoB in 1 m KOH. (a-1–b-1) Corresponding overpotential bar graphs of HER/OER at 50, 100 and 300 different current densities.

**S-2.1.7. Scan rate control on Co/FeMoB MP**

**
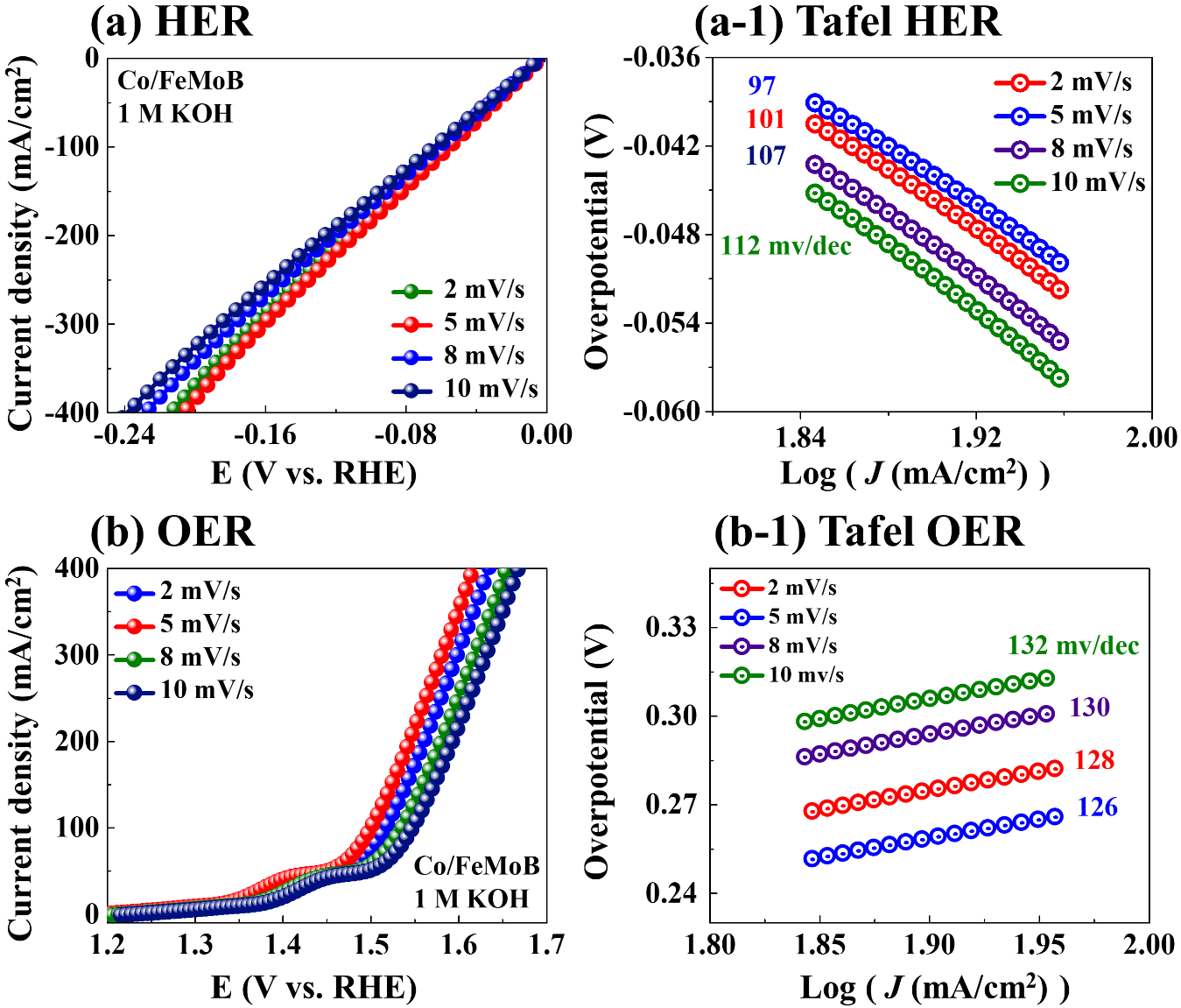
**

**Figure S8.** LSV and Tafel along with the scan rate variation. (a–b) HER/OER performance of the best Co/FeMoB at different scan rates between 2 ~ 10 mV/s in 1 _M_ KOH. (a-1–b-1) Tafel slopes at different scan rates for HER/OER. The related discussion can be found in SI text (S-1.5).

**S-2.1.8. EIS voltage control on Co/FeMoB MP**

**
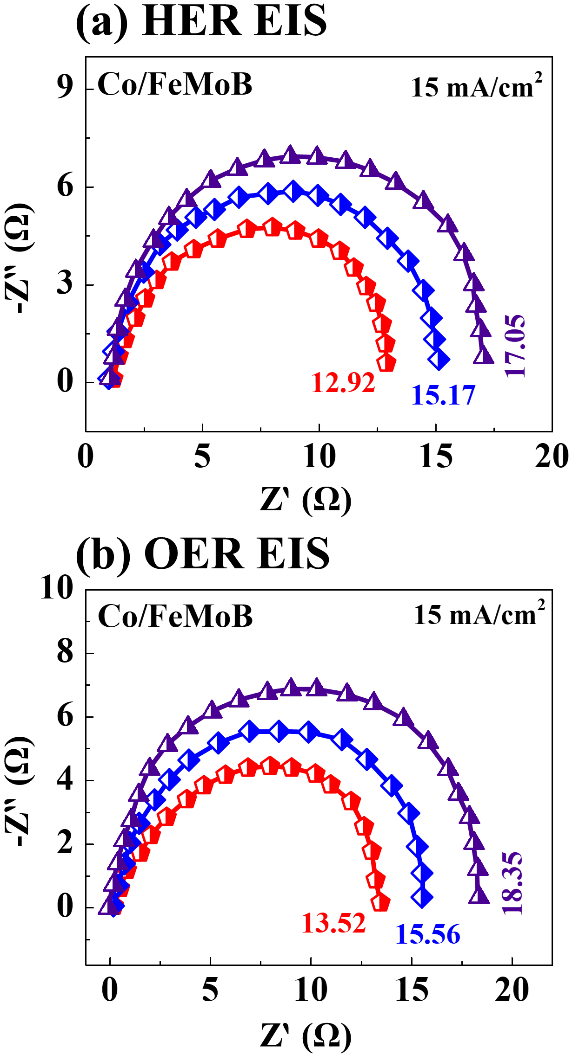
**

**Figure S9.** Voltage variation electrochemical impedance spectroscopy (EIS). (a–b) HER/OER EIS of Co/FeMoB. EIS plots at different applied voltages for the optimized Co/FeMoB electrode show that the charge transfer resistance (R_ct_) varies across the applied voltage range, particularly around the turnover region. More detailed discussion can be found in the SI text (S-1.5).

**S-2.1.9. HER benchmark Pt/C electrode**

**
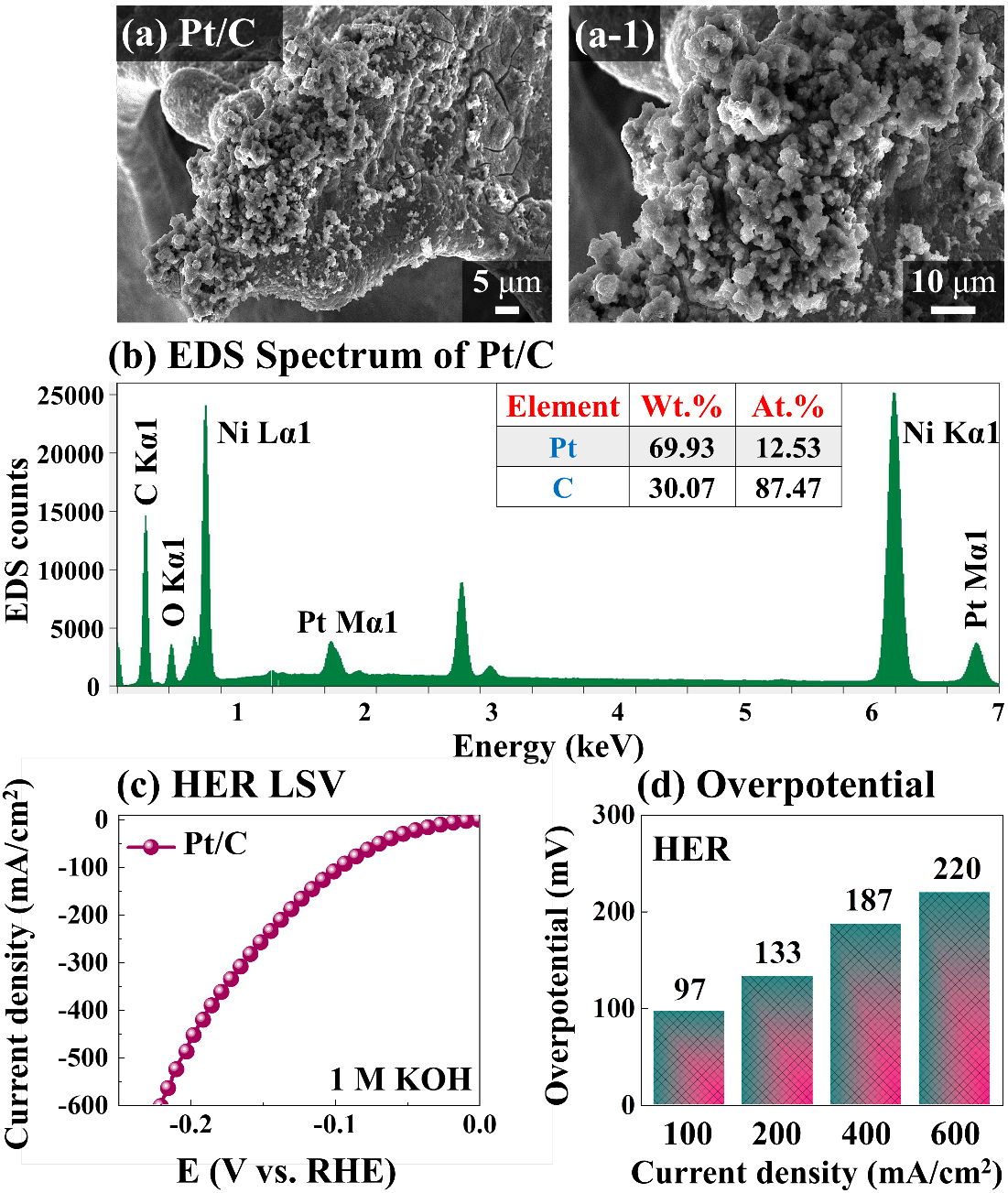
**

**Figure S10.** HER benchmark Pt/C electrode. (a–a-1) SEM micrographs. (b) EDS spectrum with the elemental Wt. & At. percentage summary. (c) HER polarization LSV curve in 1 m KOH. (d) HER overpotentials at different current densities. The Pt/C fabrication-related details can be found in SI text (S-1.6).

**S-2.1.10. OER benchmark RuO_2_ electrode**

**
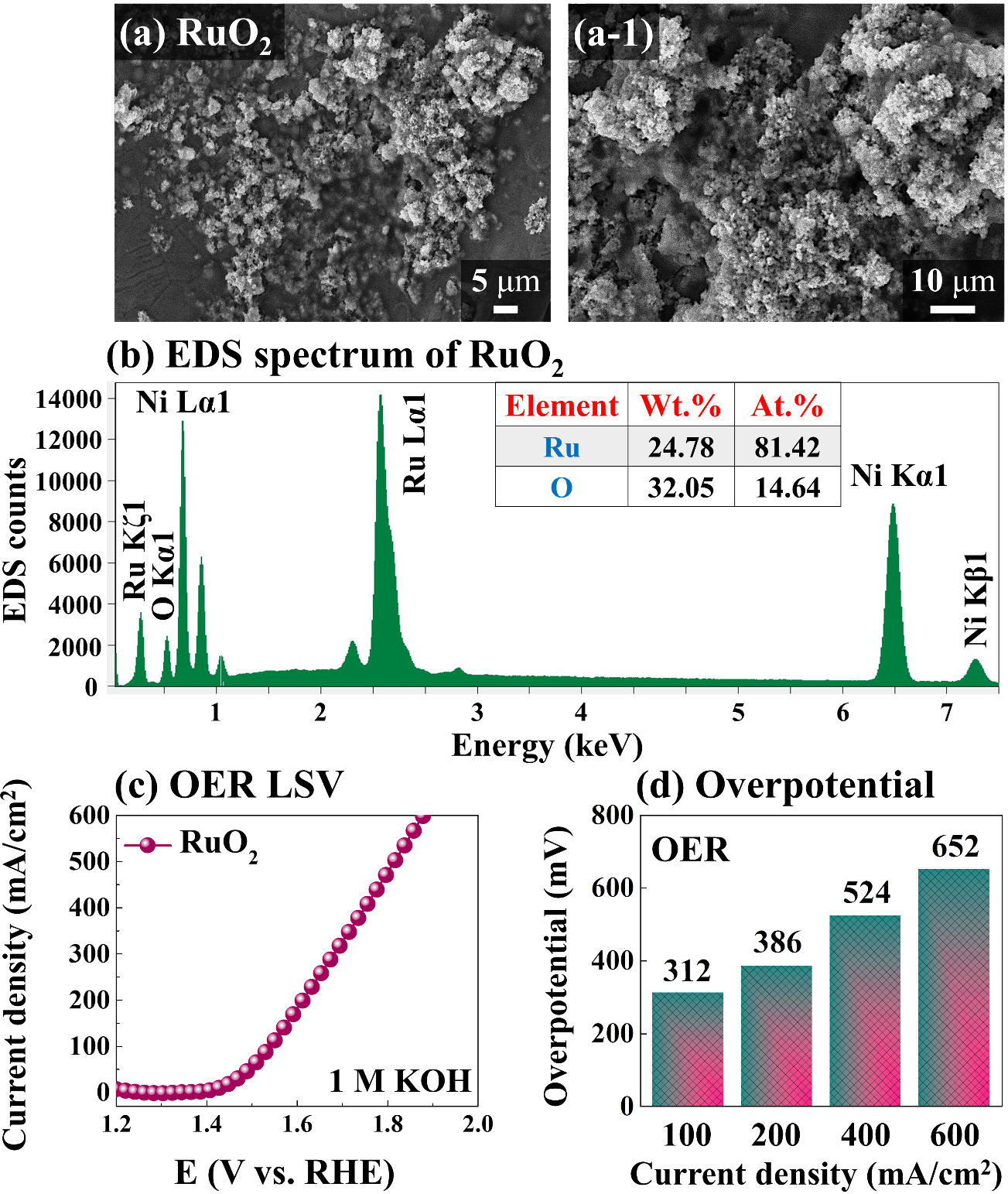
**

**Figure S11.** OER benchmark RuO_2_ electrode. (a–a-1) SEM micrographs. (b) EDS spectrum with the elemental percentage (%) summary. (c) HER polarization LSV curve in 1 m KOH. (d) HER overpotentials at 100, 200, 400 and 600. The RuO_2_ fabrication-related details can be found in SI text (S-1.6).

**S-2. Optimization of Co/FeMoB catalyst**

**S-2.1. Substate and other necessities**

**S-2.1.1. Schematic of FeMoB MP fabrication route**

**S-2.1.2. Bare nickel foam (NF) characterization**

**S-2.1.3. Base template FeMoB: SEM & EDS spectrum**

**S-2.1.4. Base template FeMoB: EDS mapping & line profile**

**S-2.1.5. Base template FeMoB: Raman & XRD**

**S-2.1.6. Base template FeMoB: Electrochemical LSV**

**S-2.1.7. Scan rate control on Co/FeMoB MP**

**S-2.1.8. EIS voltage control on Co/FeMoB MP**

**S-2.1.9. HER benchmark Pt/C electrode**

**S-2.1.10. OER benchmark RuO_2_ electrode**

**S-2.2. Co-doping optimization parameter and system**

**S-2.2.1 Hydrothermal: Co concentration**

**S-2.2.2. Hydrothermal: Reaction duration**

**S-2.2.3. Hydrothermal: Reaction temperature**

**S-2.2.4. Hydrothermal: Annealing duration**

**S-2.2.5. Hydrothermal: Annealing temperature**

**S-2.2.6. Annealing temperature: EDS**

**S-2.2.7. Annealing temperature: CV**

**S-2.2.8. Annealing temperature: C_dl_ valuesS-2.2.1 Hydrothermal: Co concentration**

**
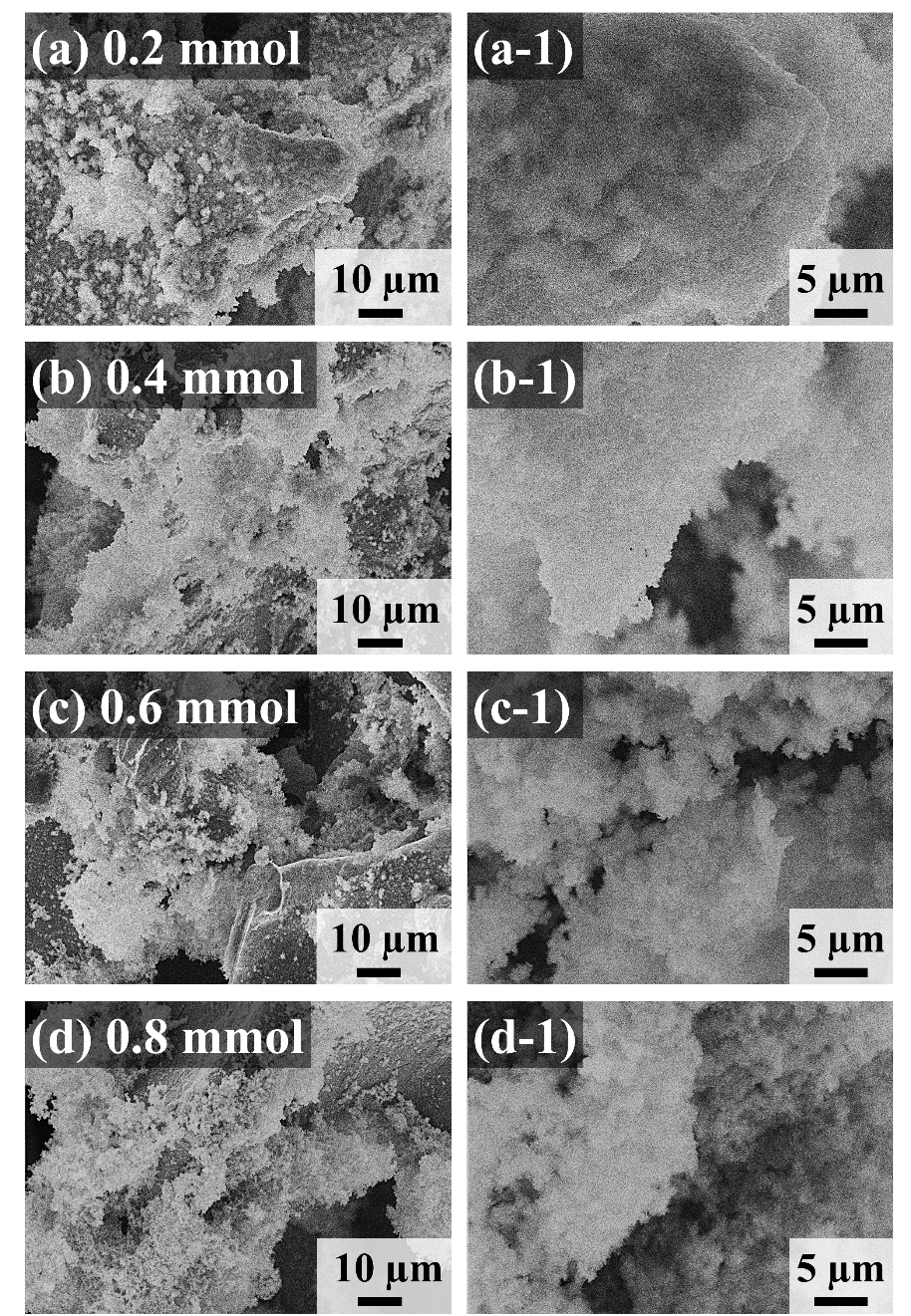
**

**Figure S12.** Fabrication of Co/FeMoB electrodes with the hydrothermal Co concentration control between 0.2 mmol ~ 0.8 mmol. The Co was hydrothermally doped on the base FeMoB electrode at 180 ºC for 12 hours in a microwave reactor chamber. (a–d) SEM micrographs of Co/FeMoB electrodes. (a-1–d-1) Corresponding zoomed-in SEM micrographs.

**S-2.2.1 Hydrothermal: Co concentration**

**
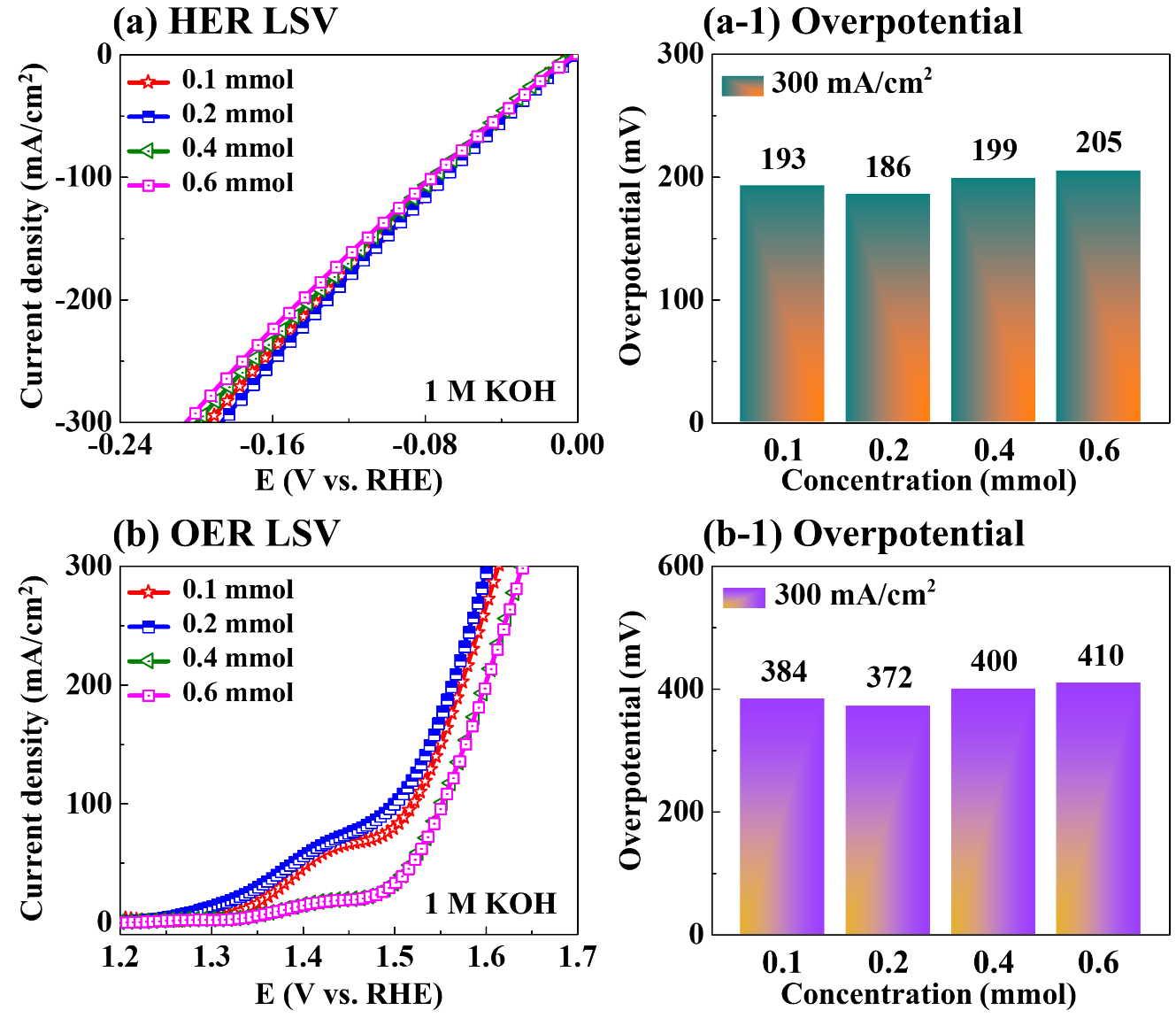
**

**Figure S13.** (a–b) HER/OER polarization LSV curves of hydrothermal Co concentration control set. (a–b) Corresponding overpotential bar graphs at 300 mA/cm^2^ current density. (a-2–b-2) The 0.2 mmol Co/FeMoB electrode showed the best HER/OER performance in this set.

**S-2.2.2. Hydrothermal: Reaction duration**

**
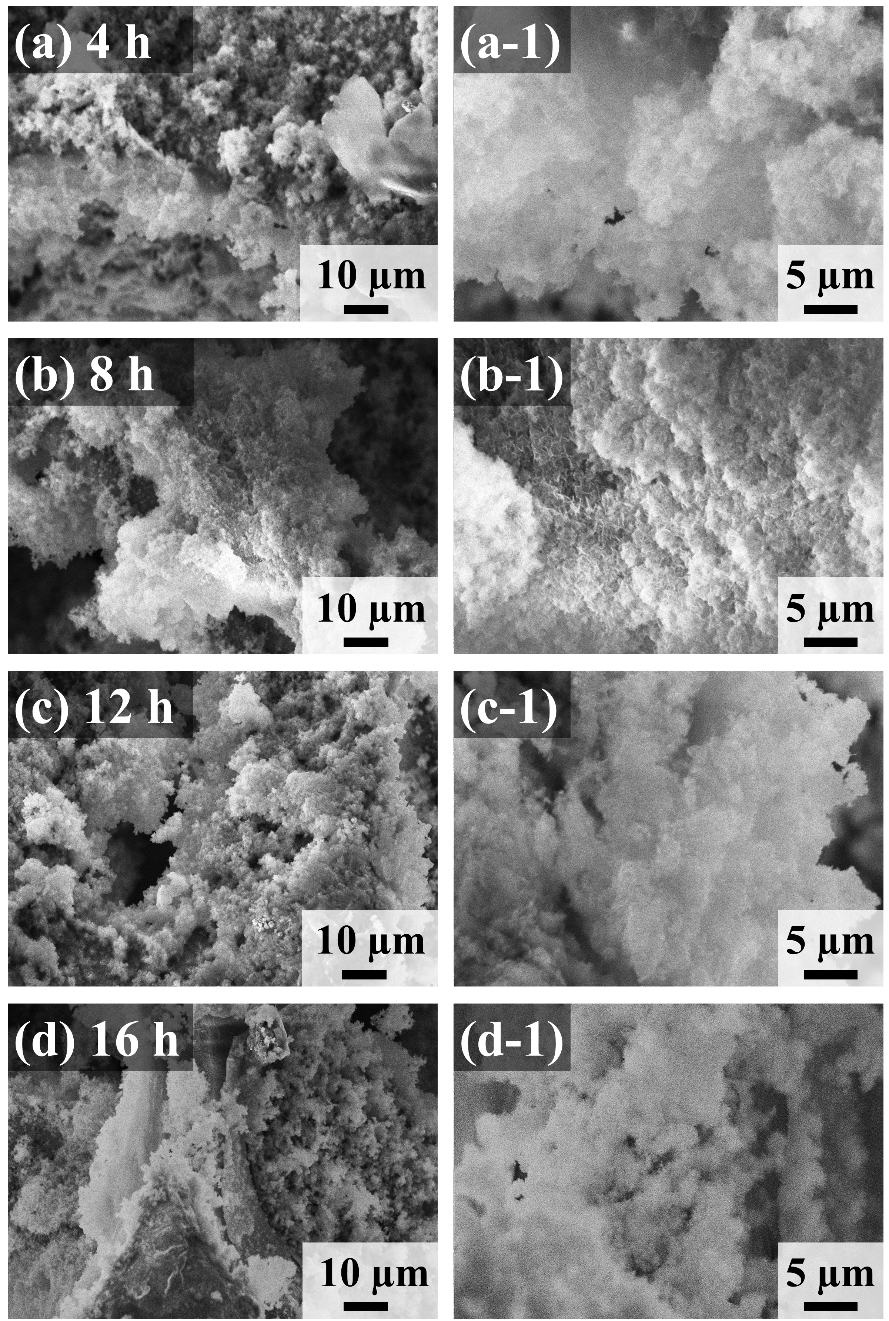
**

**Figure S14.** Hydrothermal reaction duration control between 4 ~ 16 hours at 180 ºC. (a–d) SEM micrographs of Co/FeMoB electrodes. (a-1–d-1) Zoom-in views of SEM images.

**S-2.2.2. Hydrothermal: Reaction duration**

**
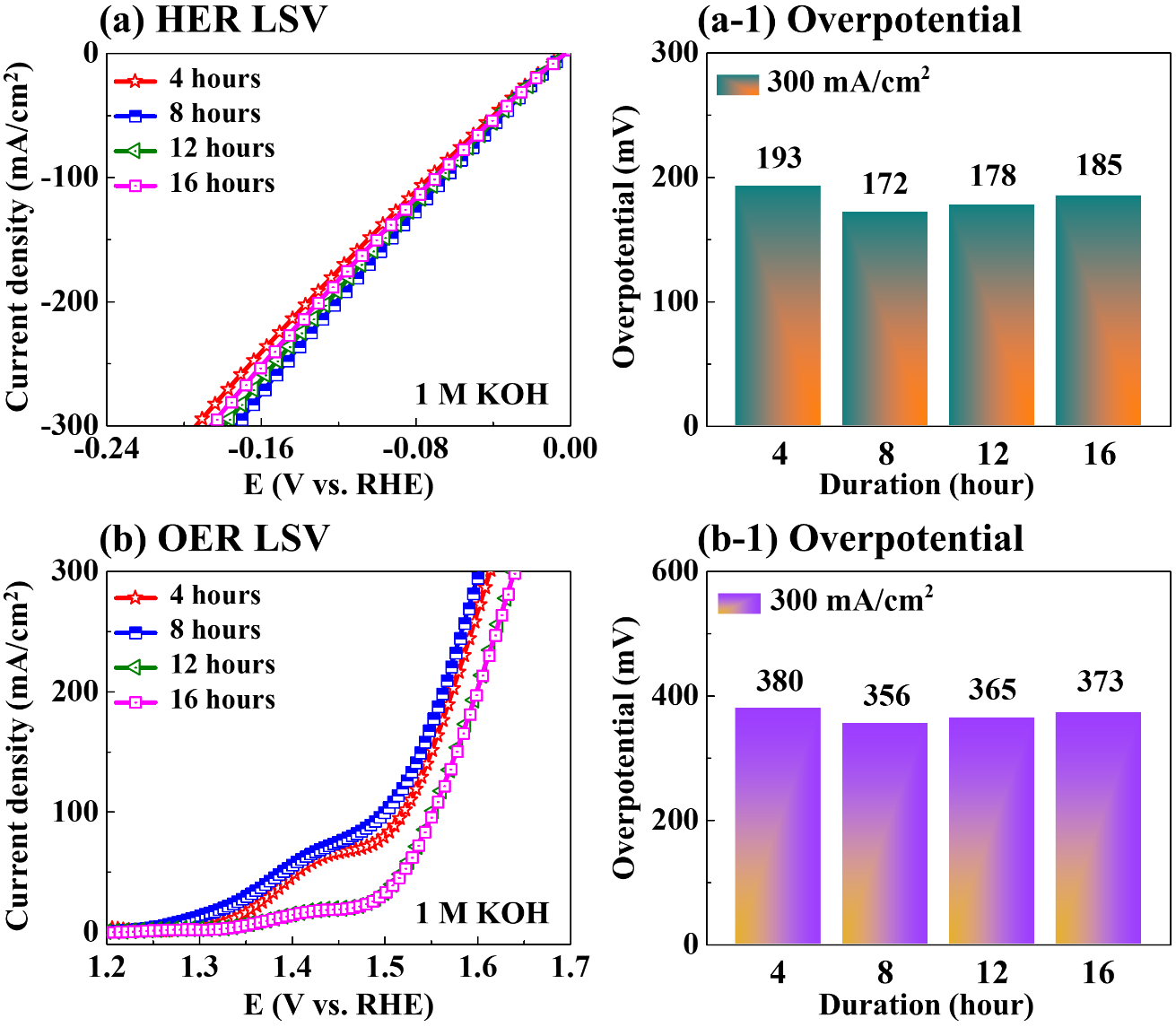
**

**Figure S15.** (a–b) HER/OER polarization LSV curves in 1 m KOH. (a–b) Bar graphs of corresponding overpotential values. (a-2–b-2) The Co/FeMoB electrode fabricated for 8 hours showed the best HER/OER performance in this set.

**S-2.2.3. Hydrothermal: Reaction temperature**

**
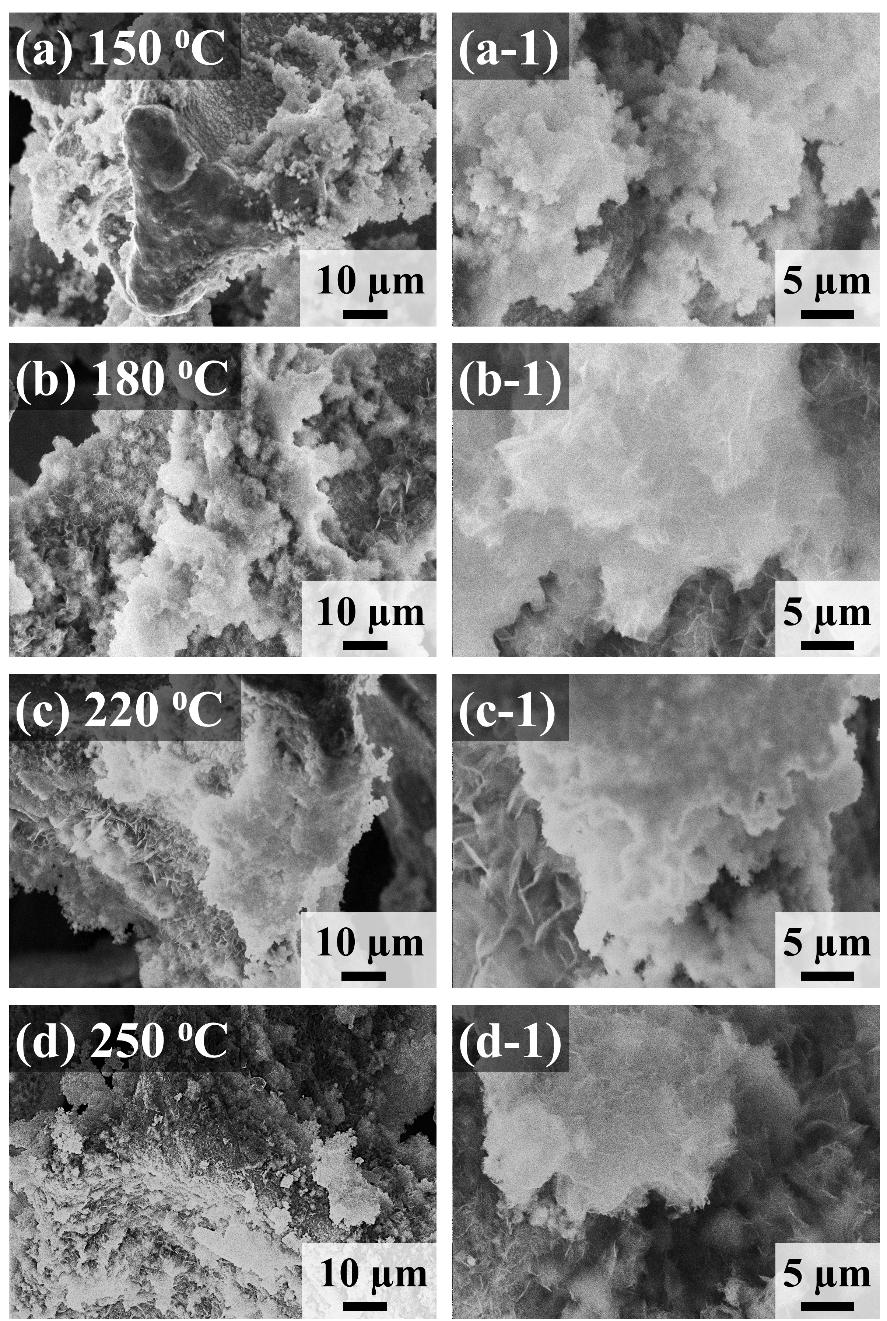
**

**Figure S16.** Hydrothermal reaction temperature control. The reaction temperature was varied from 150 to 250 ºC for 8 hours. (a–d) SEM micrographs. (a-1–d-1) Zoom-in views.**S-2.2.3. Hydrothermal: Reaction temperature**

**
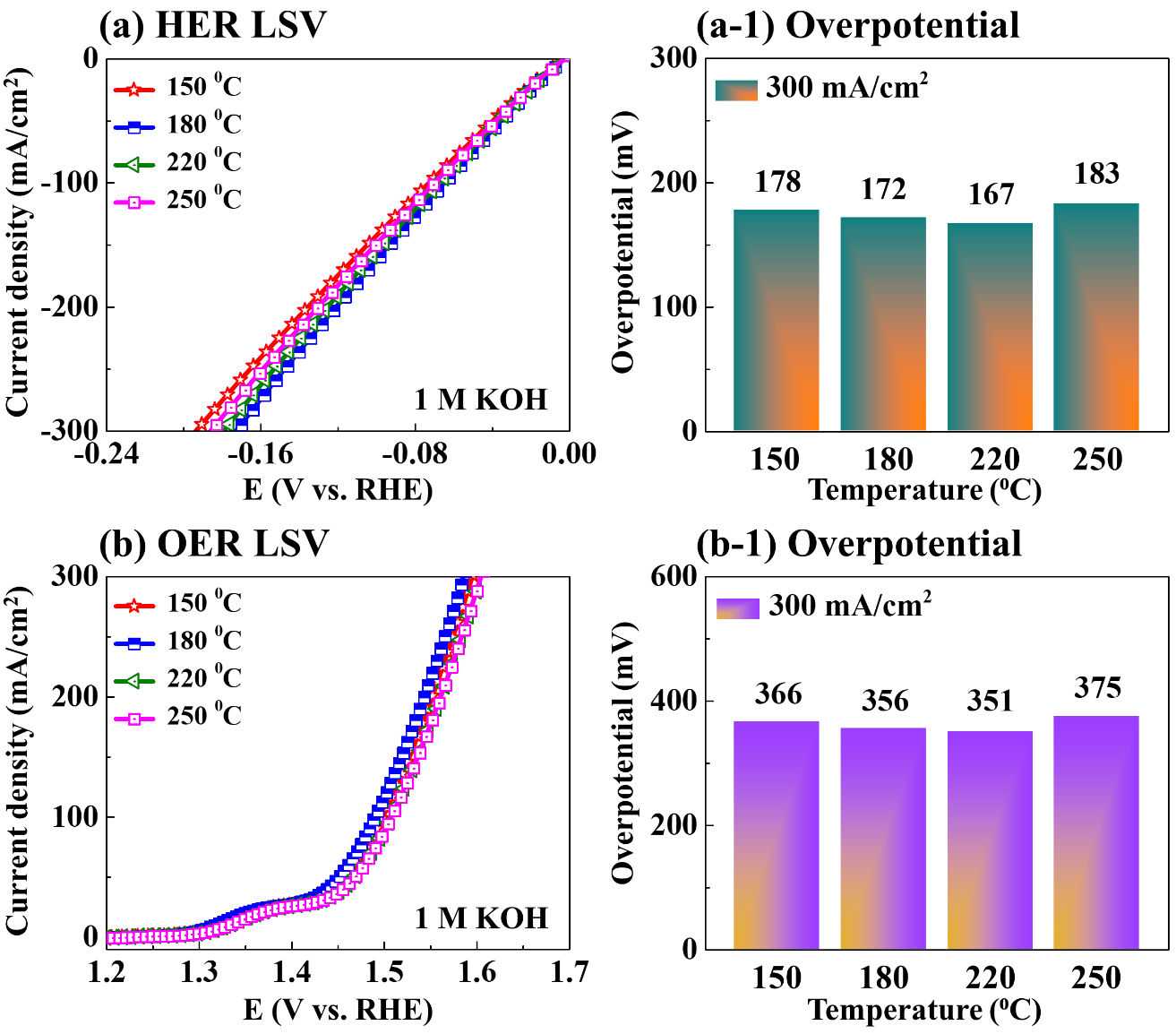
**

**Figure S17.** (a–b) HER/OER LSV polarization curves in 1 m KOH. (a–b) Corresponding overpotential bar graphs. (a-2)–(b-2) The 180 ºC Co/FeMoB electrode showed the best HER/OER performances in this set.**S-2.2.4. Hydrothermal: Annealing duration**

**
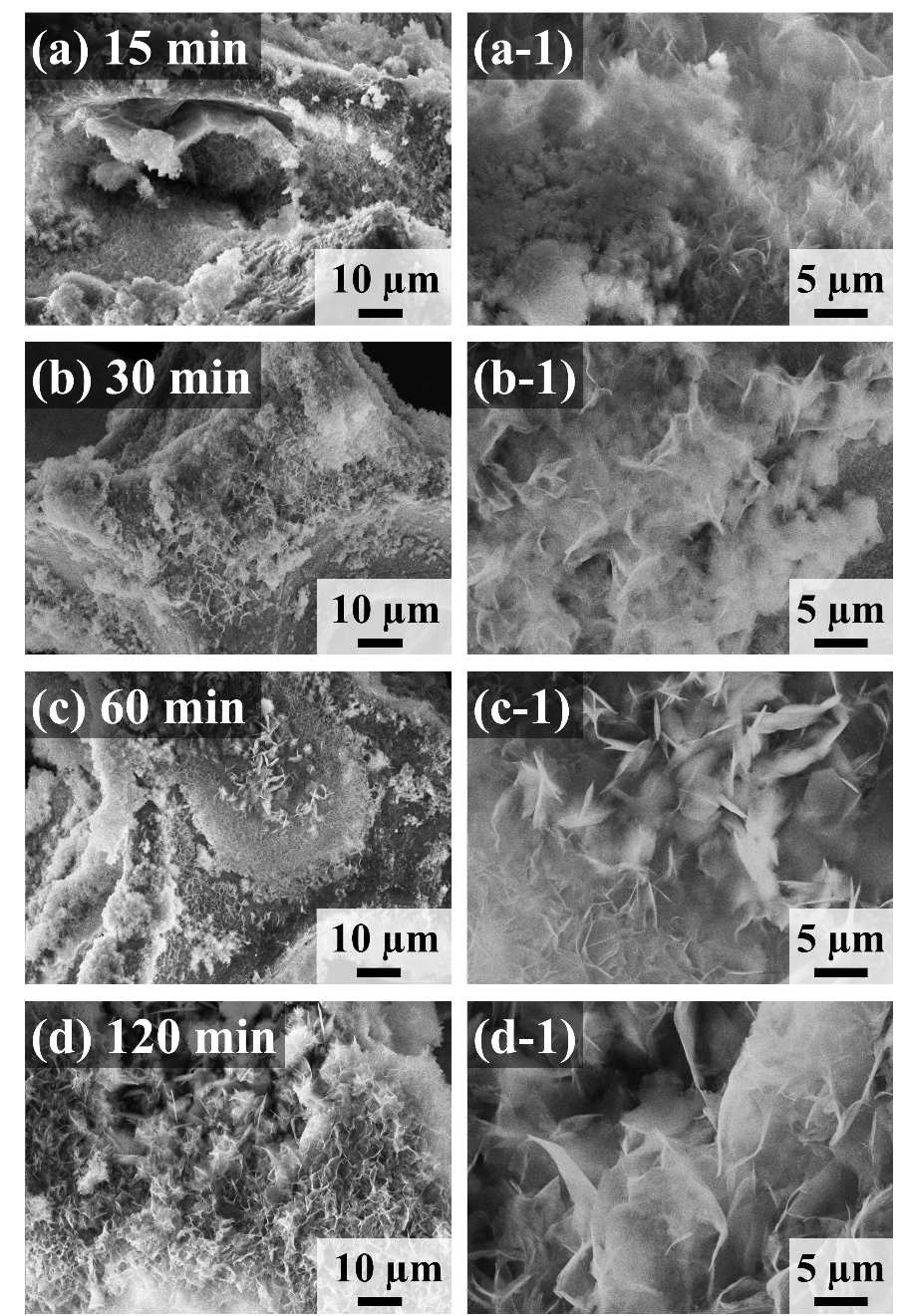
**

**Figure S18.** Post-annealing duration control between 15 ~ 120 min at 100 ℃ in RTP chamber. (a–d) SEM micrographs of Co/FeMoB electrodes. (a-1–d-1) Enlarged views of the micrographs.**S-2.2.4. Hydrothermal: Annealing duration**

**
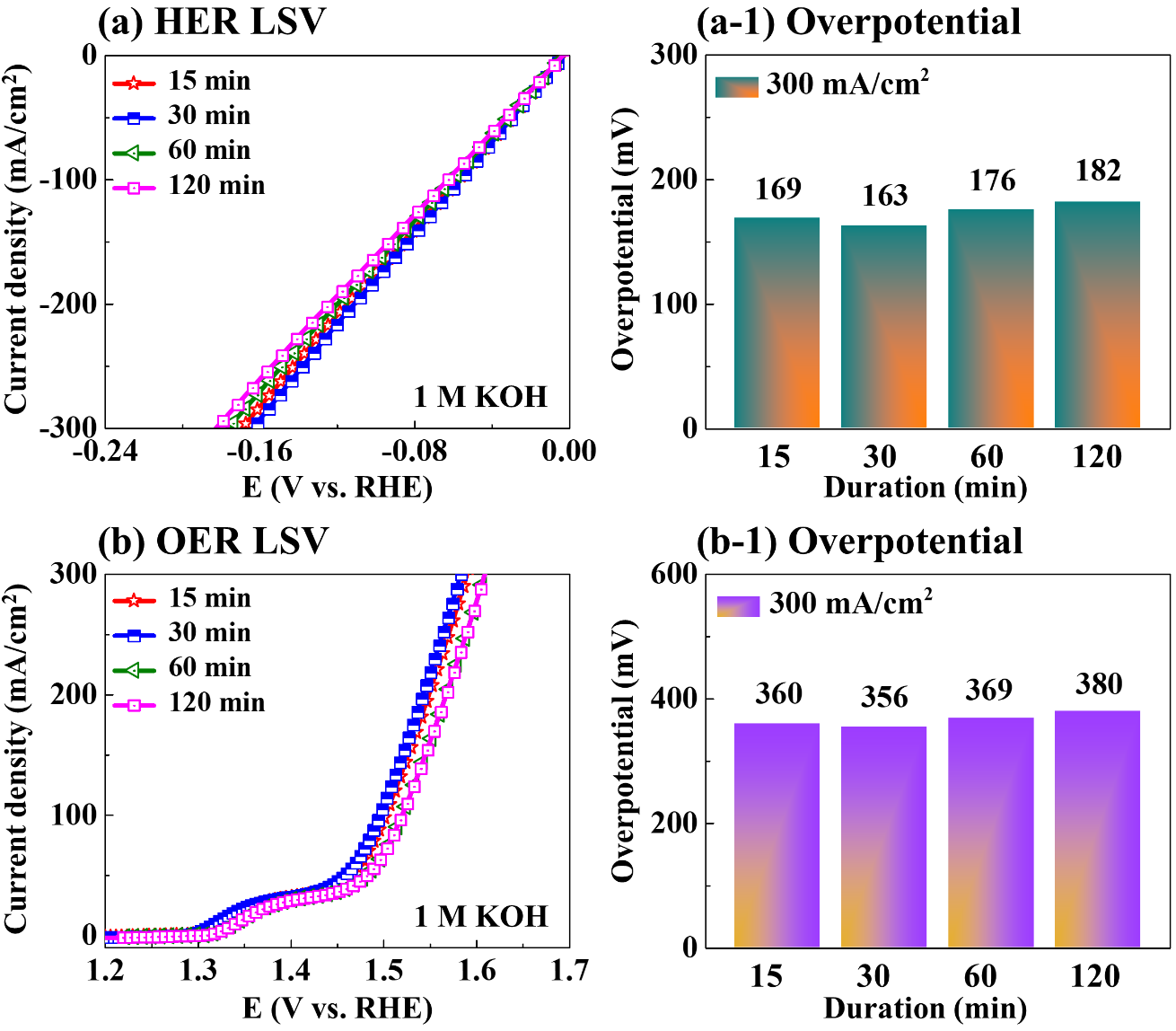
**

**Figure S19.** (a–b) HER/OER LSV curves. (a–b) Overpotential bar graph summary. (a-2–b-2) The 30 min electrode showed the best HER/OER performances in this set.**S-2.2.5. Hydrothermal: Annealing temperature**

**
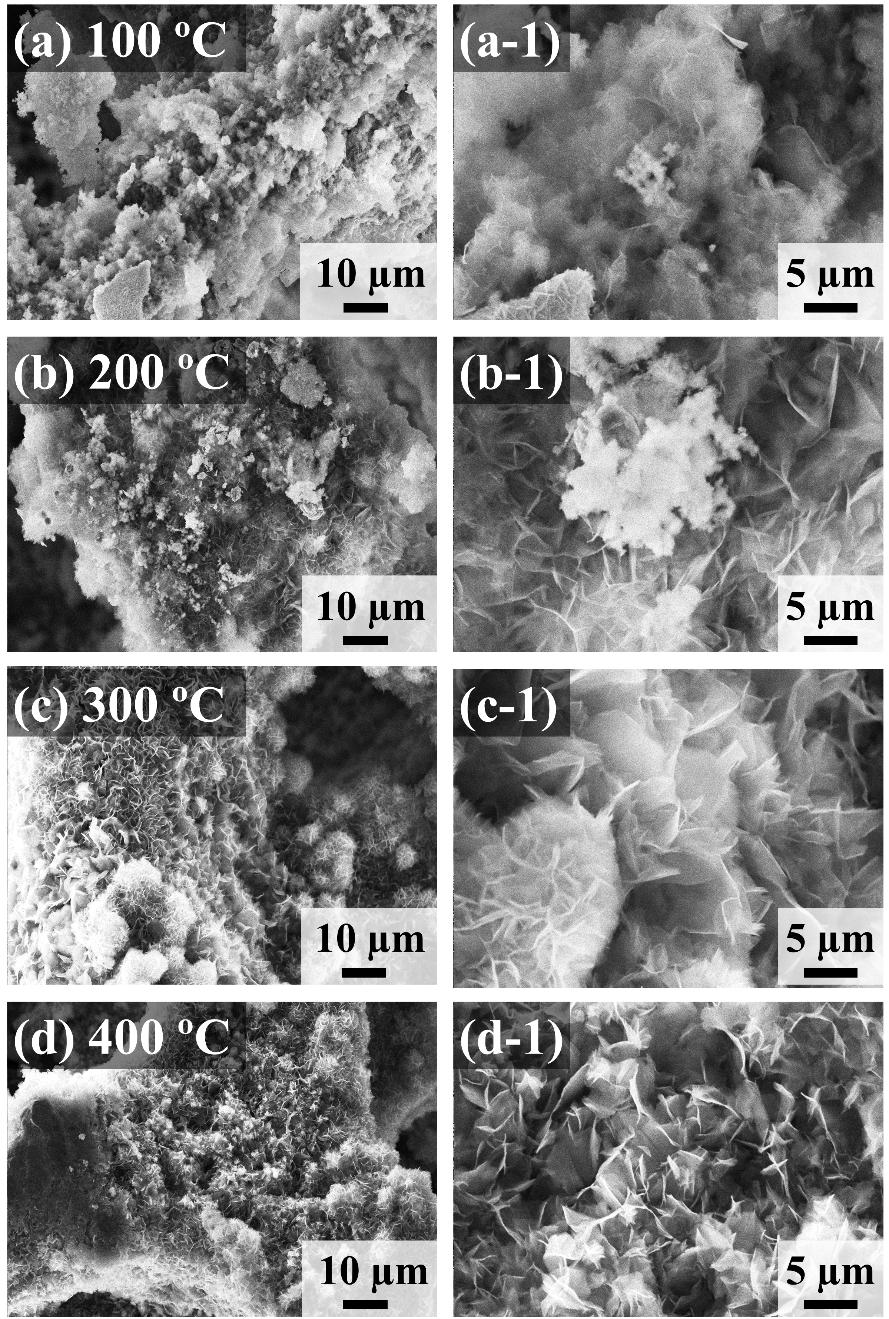
**

**Figure S20.** Post-annealing temperature control set between 100 ~ 400 ⁰C for 30 min. (a–d) SEM micrographs of Co/FeMoB. (a-1–d-1) Zoomed-in views of SEM micrographs.**S-2.2.6. Annealing temperature: EDS spectra**

**
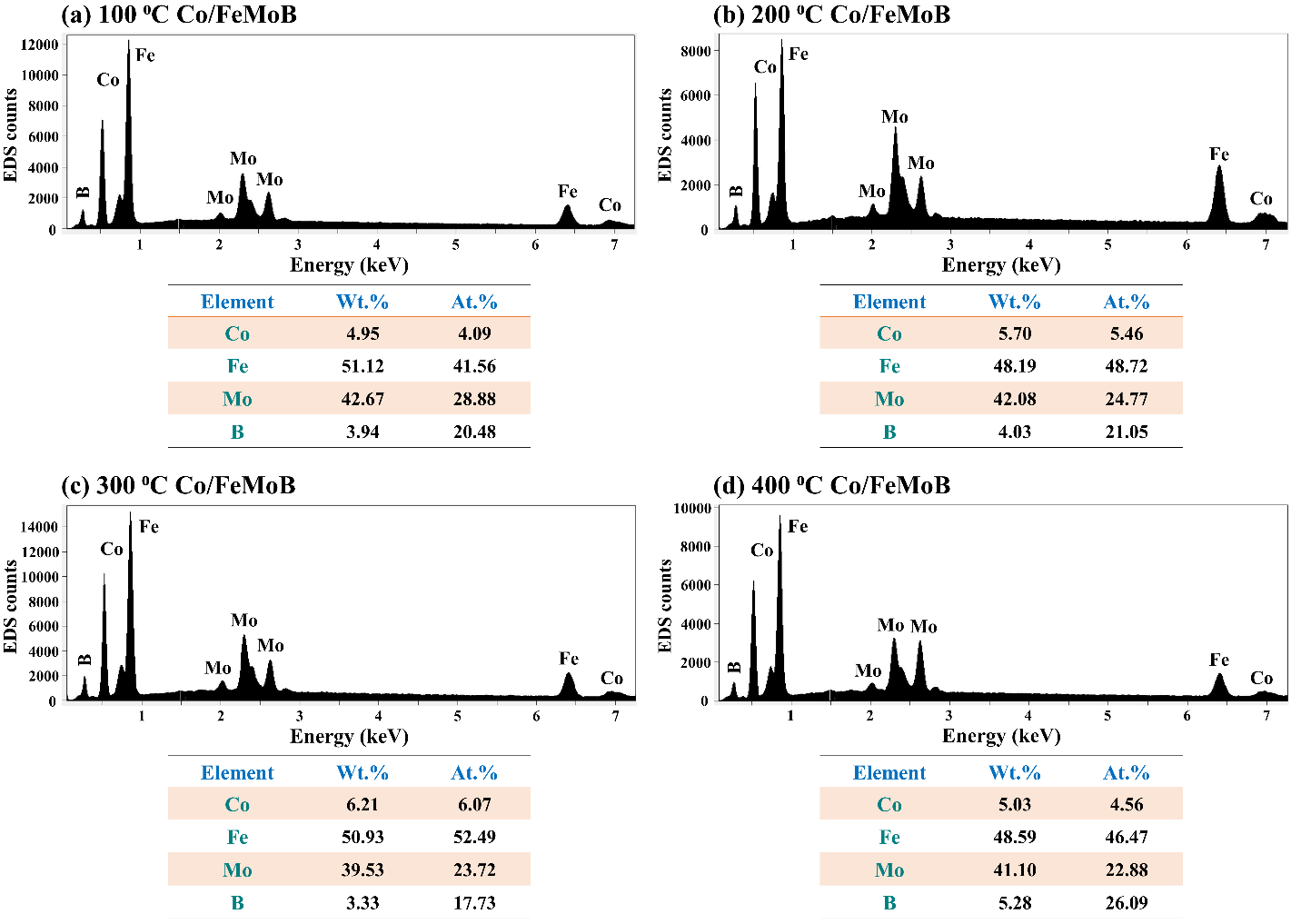
**

**Figure S21.** EDS spectra of post-annealing temperature control set of Co/FeMoB electrodes. (a–d) Corresponding Wt. and At. percentages (%) without nickel foam (NF) substrate.

**S-2.2.7. Annealing temperature: CV curves (HER)**

**
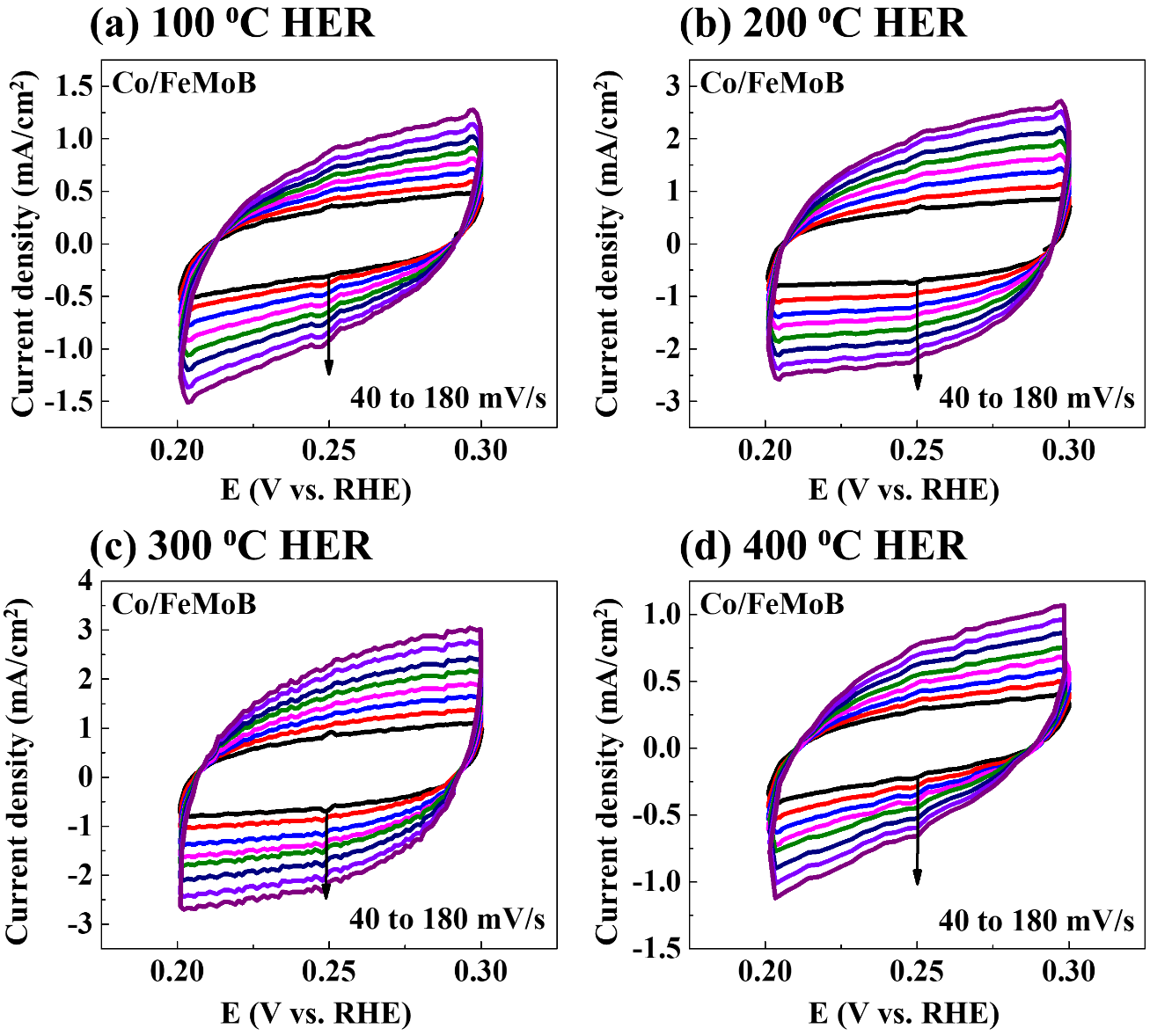
**

**Figure S22.** (a–d) HER cyclic voltammetry (CV) curves for the post-annealing temperature control set of Co/FeMoB electrode.

**S-2.2.7. Annealing temperature: CV curves (OER)**

**
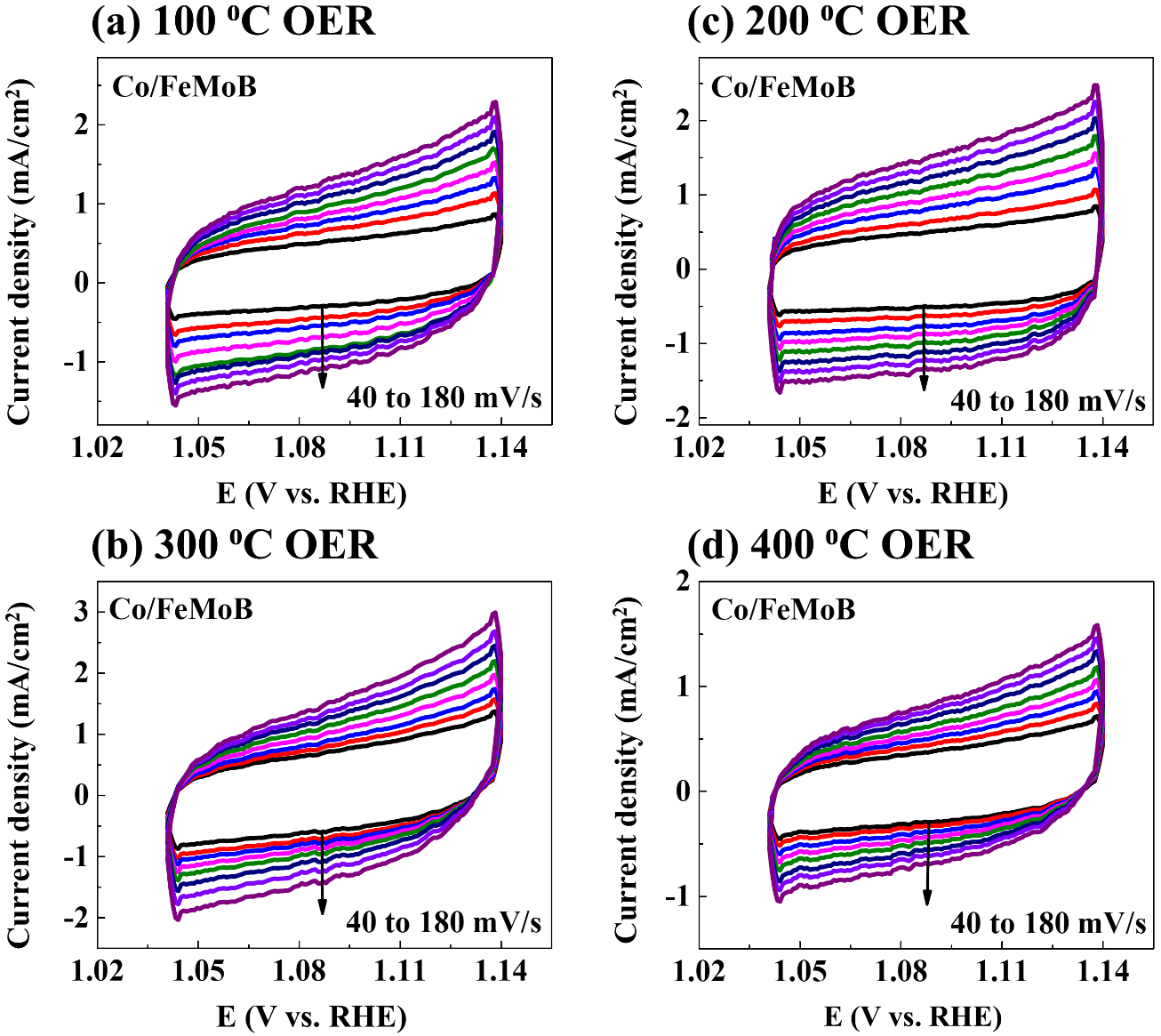
**

**Figure S23.** (a–d) OER cyclic voltammetry (CV) curves for the post-annealing temperature control set of Co/FeMoB electrode.

**S-2.2.8. Annealing temperature: C_dl_ values**

**
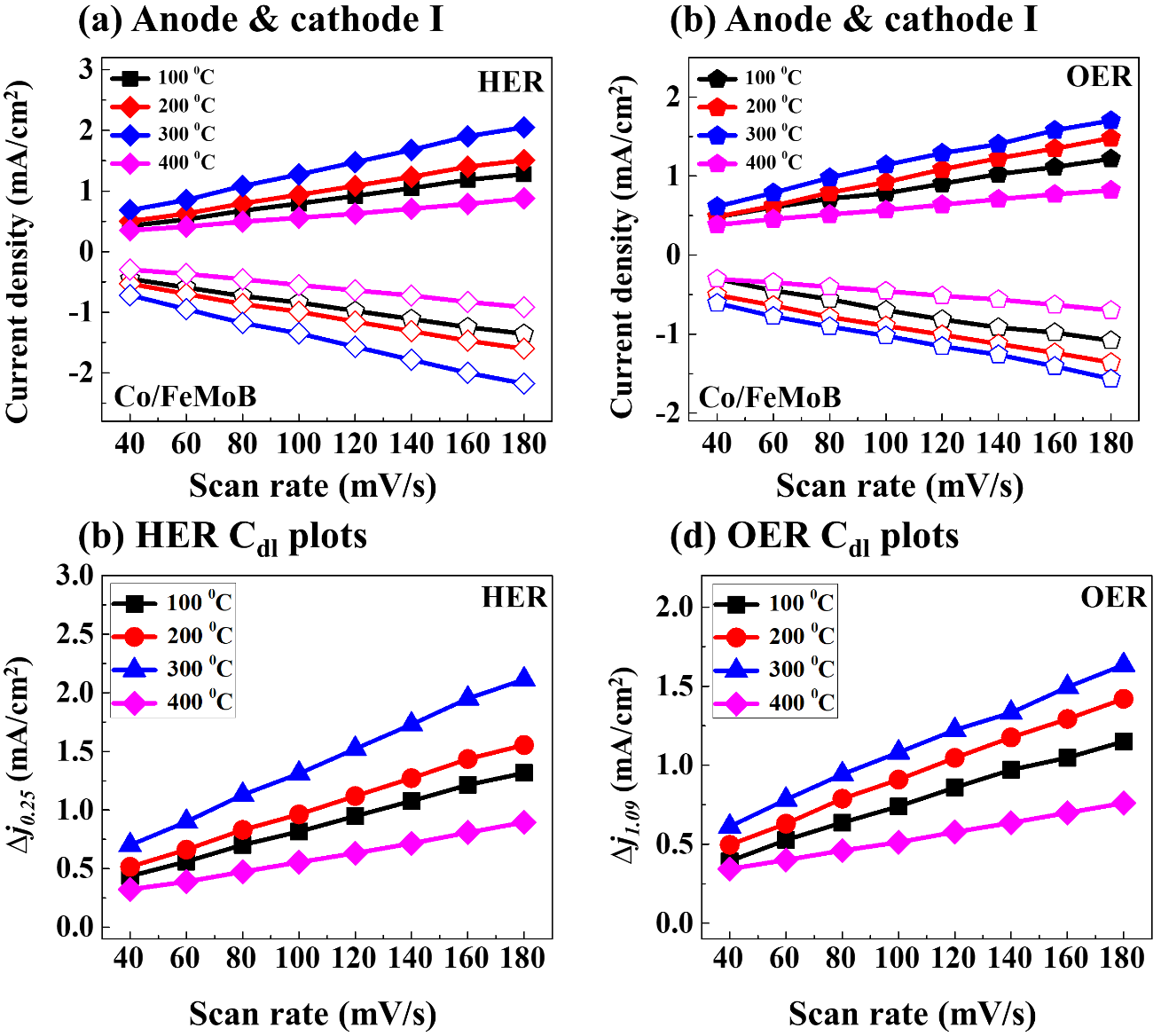
**

**Figure S24.** (a–b) Anodic and cathodic current density versus scan rates for the HER/OER CV performance. (a-1–b-1) Double-layer capacitance (C_dl_) analysis for Co/FeMoB electrodes of post-annealing duration control set.

**S-2.2.8. Annealing temperature: C_dl_ values**

**
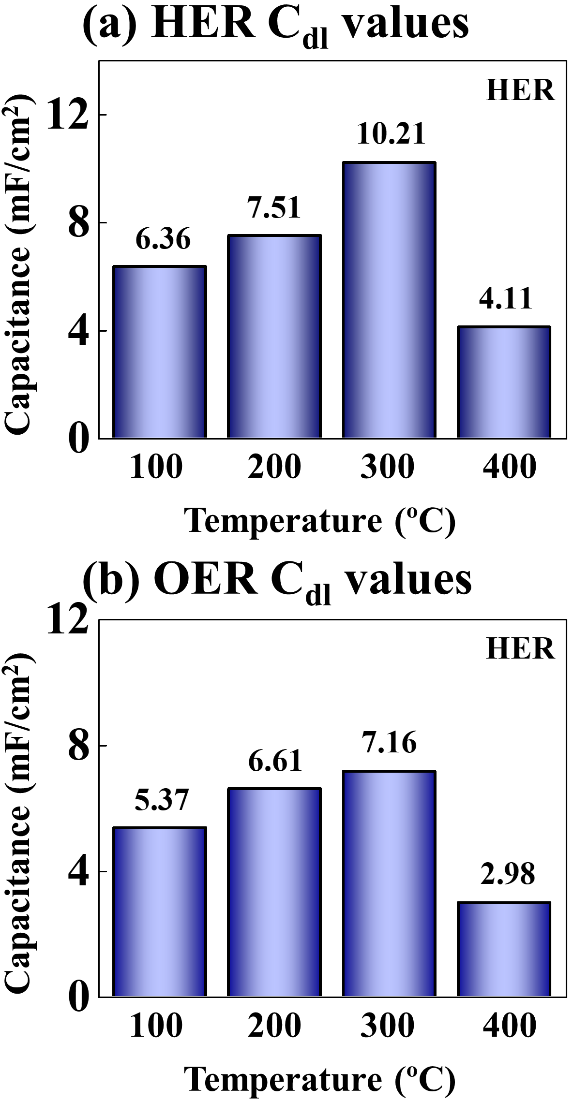
**

**Figure S25.** Double-layer capacitance (C_dl_) of post-annealing temperature control set. (a–b) HER/OER C_dl_ values were calculated from HER/OER CV curves.

**S-3. Analysis of the best Co/FeMoB catalyst**

**S-3.1. Physical and Electrochemical analysis**

**S-3.1.1. Physical: CS EDS**

**S-3.1.2. Physical: HR-TEM**

**S-3.1.3. Physical: Raman**

**S-3.1.4. Physical: XRD pattern**

**S-3.1.5. Electrochemical: CA/LSV**

**S-3.1.6. Electrochemical: Repeatability**

**S-3.1.7. Electrochemical: HER/OER stability**

**S-3.1.8. Electrochemical: Comparison NF LSV**

**S-3.1.9. Electrochemical: HER/OER in different pH**

**S-3.1.10. Electrochemical: different pH TOF activity**

**S-3.1.11. Electrochemical: electrolyte temperature effect**

**S-3.1.12. Electrochemical: ECSA-normalized HER/OER**

**S-3.1.13. Electrochemical: FE measurement for HER/OER**

**S-3.2. Co-doping effect on framework FeMoB catalyst**

**S-3.2.1. EDS spectra comparison**

**S-3.2.2. HER/OER EIS comparison**

**S-3.2.3. HER/OER CV curve comparison**

**S-3.2.4. HER/OER anodic/cathodic comparison**

**S-3.2.5. HER/OER overpotential comparison**

**S-3.2.6. Normalized HER/OER comparison**

**S-3.2.7. CP-stability comparison for HER/OER**

**S-3.2.8. HER/OER performance comparison**

**S-3.3. Bifunctional system of Co/FeMoB**

**S-3.3.1. Different pH operations**

**S-3.3.2. CV/LSV comparison**

**S-3.3.3. Repeatability test**

**S-3.3.4. Natural water LSV**

**S-3.3.5. Stability in NW**

**S-3.3.6. Additional CP-stability**

**S-3.4. Hybrid system of Co/FeMoB**

**S-3.4.1. Different pH operations**

**S-3.4.2. CV/LSV comparison**

**S-3.4.3. Repeatability test**

**S-3.4.4. Natural water LSV**

**S-3.5. Post-stability of Co/FeMoB**

**S-3.5.1. Micrographs of SEM**

**S-3.5.2. EDS spectra**

**S-3.5.3. Raman spectra**

**S-3.5.4. XRD pattern**

**S-3.5.5. Full-scan XPS**

**S-3.5.6. High-resolution XPS**

**S-3.5.7. 3-E HER/OER LSV**

**S-3.5.7. 2-E OWE LSV**

**S-3.1.1. Physical: CS-EDS map**

**
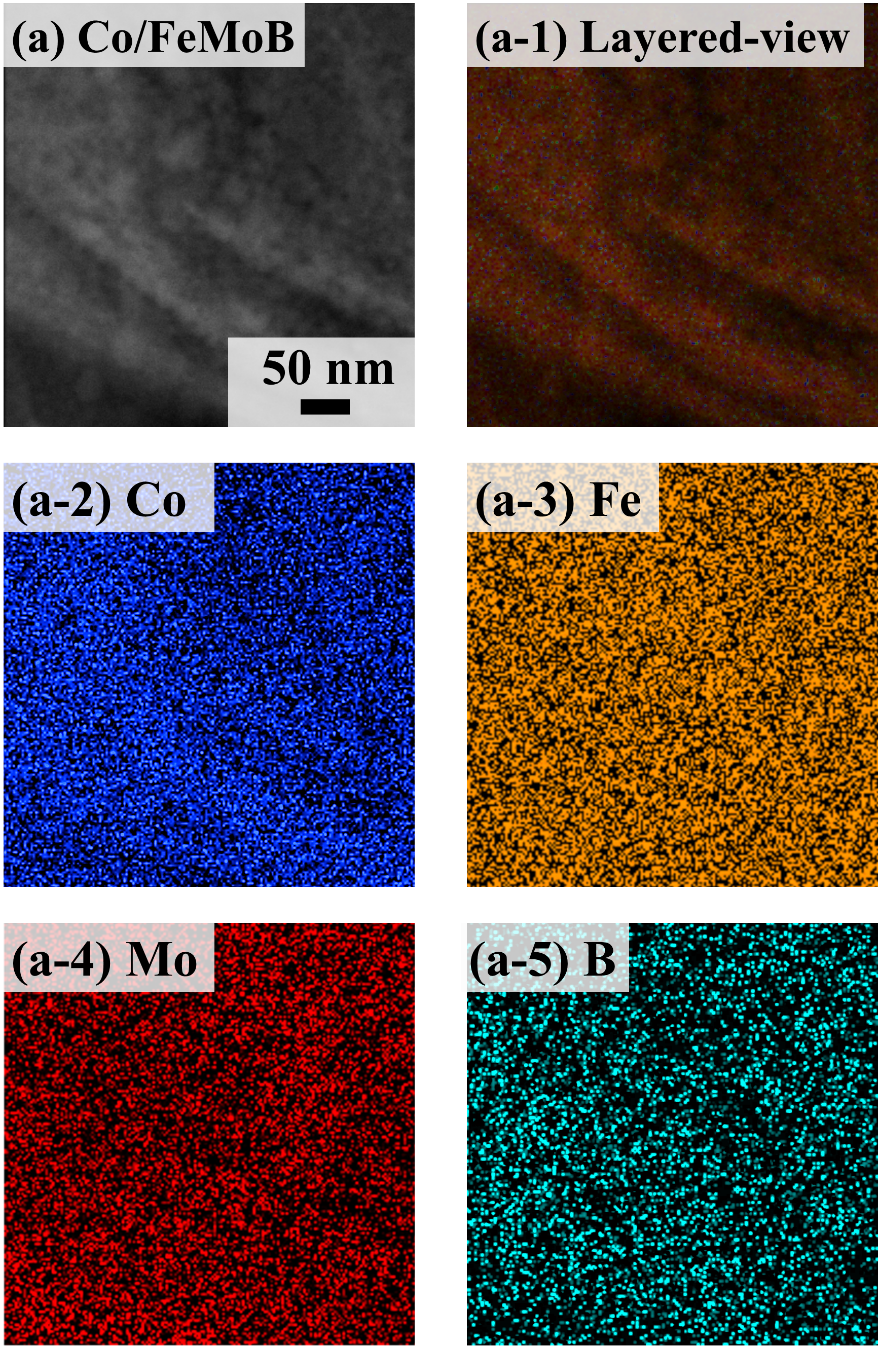
**

**Figure S26.** Cross-sectional (CS) EDS maps showing the homogenous distribution of Co, Fe, Mo and B. (a-1–a-5) CS-EDS phase maps of Co, Fe, Mo and B.

**S-3.1.2. Physical: HR-TEM**

**
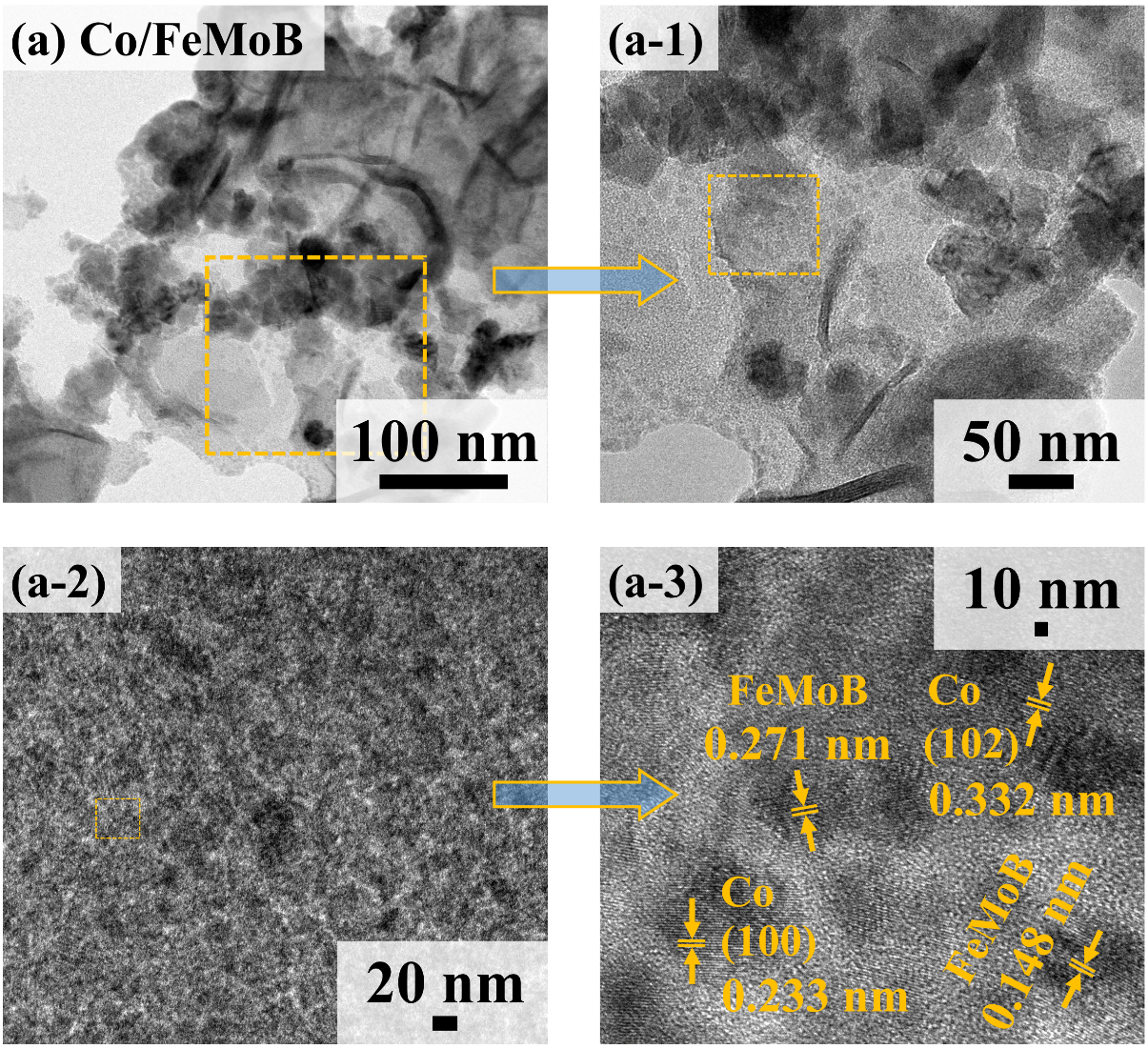
**

**Figure S27.** (a–a-2) High-resolution transmission electron microscopy (HR-TEM) images at different magnifications. (a-3) Zoomed-in view. The Co phase exhibited interplanar spacings of 0.233 and 0.332 nm, whereas the FeMoB phase displayed atomic layer spacings of 0.148 and 0.271 nm.

**S-3.1.3. Physical: Raman**

**
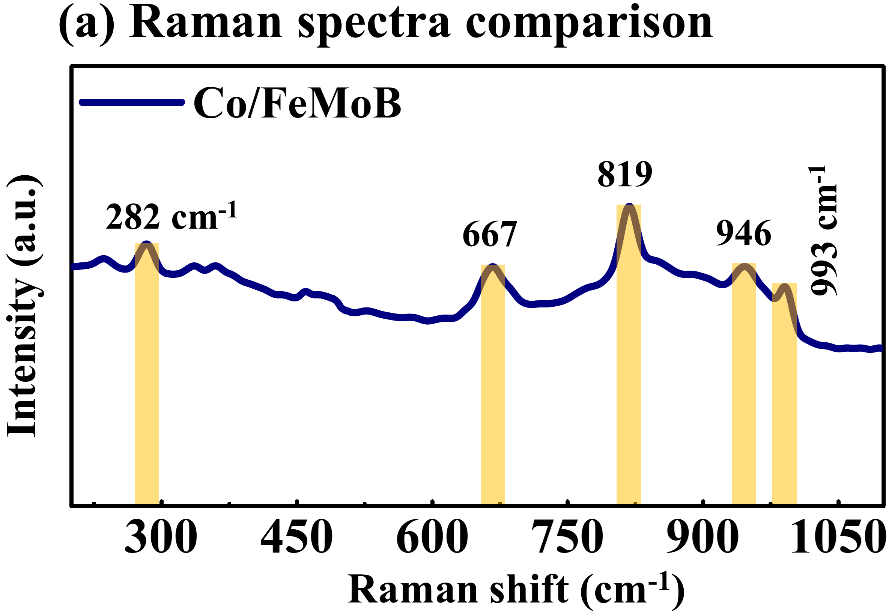
**

**Figure S28.** Raman spectroscopy on Co/FeMoB electrode. (a) Raman signal of Co/FeMoB.

**S-3.1.4. Physical: XRD pattern**

**
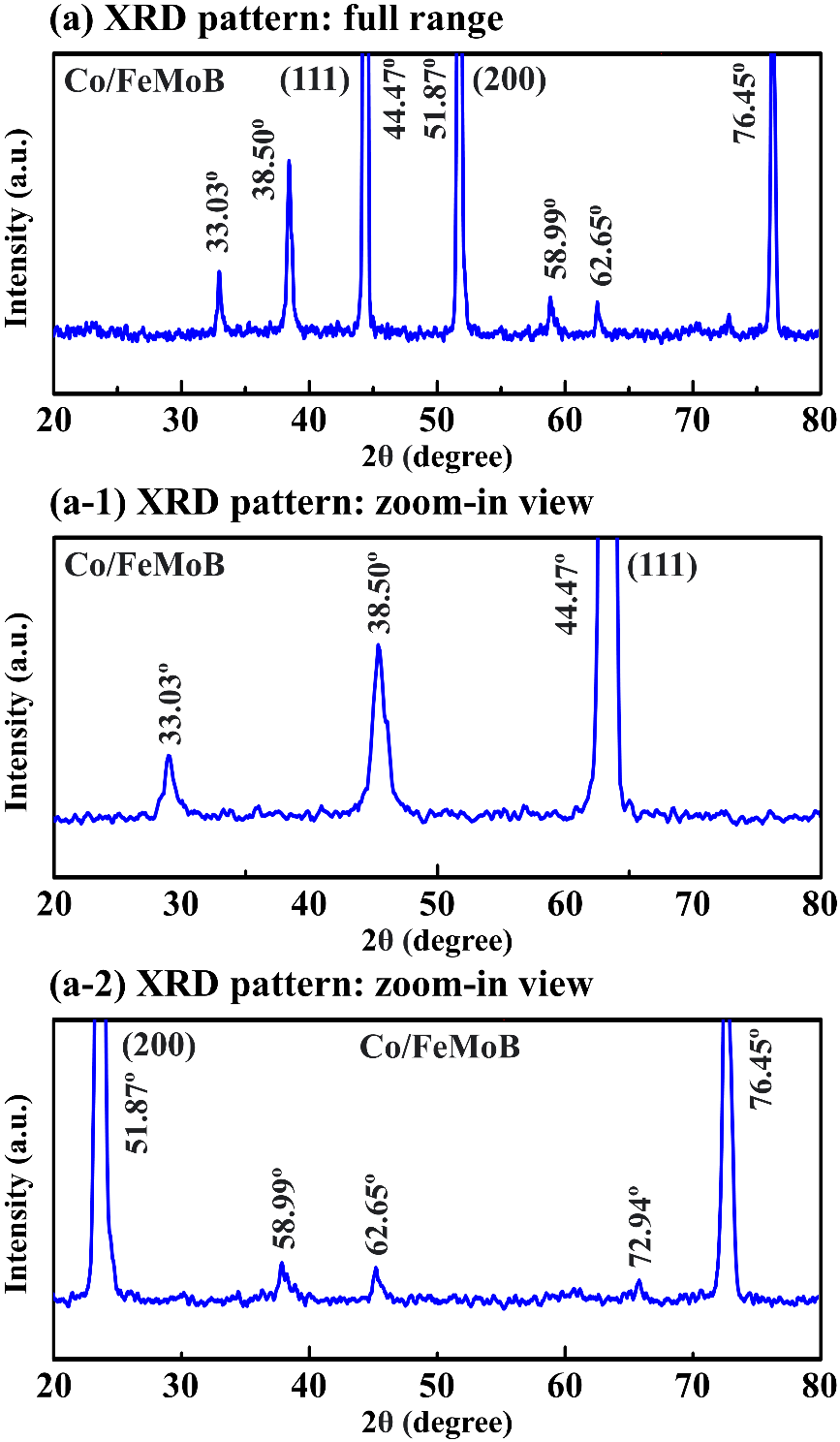
**

**Figure S29.** X-ray diffraction (XRD) patterns and structural analysis of optimized Co/FeMoB catalyst. (a) Full-scan XRD pattern of Co/FeMoB in 20 ~ 80⁰ region. (a-1–a-2) Zoom-in view of XRD.

**S-3.1.5. Electrochemical: CA/LSV**

**
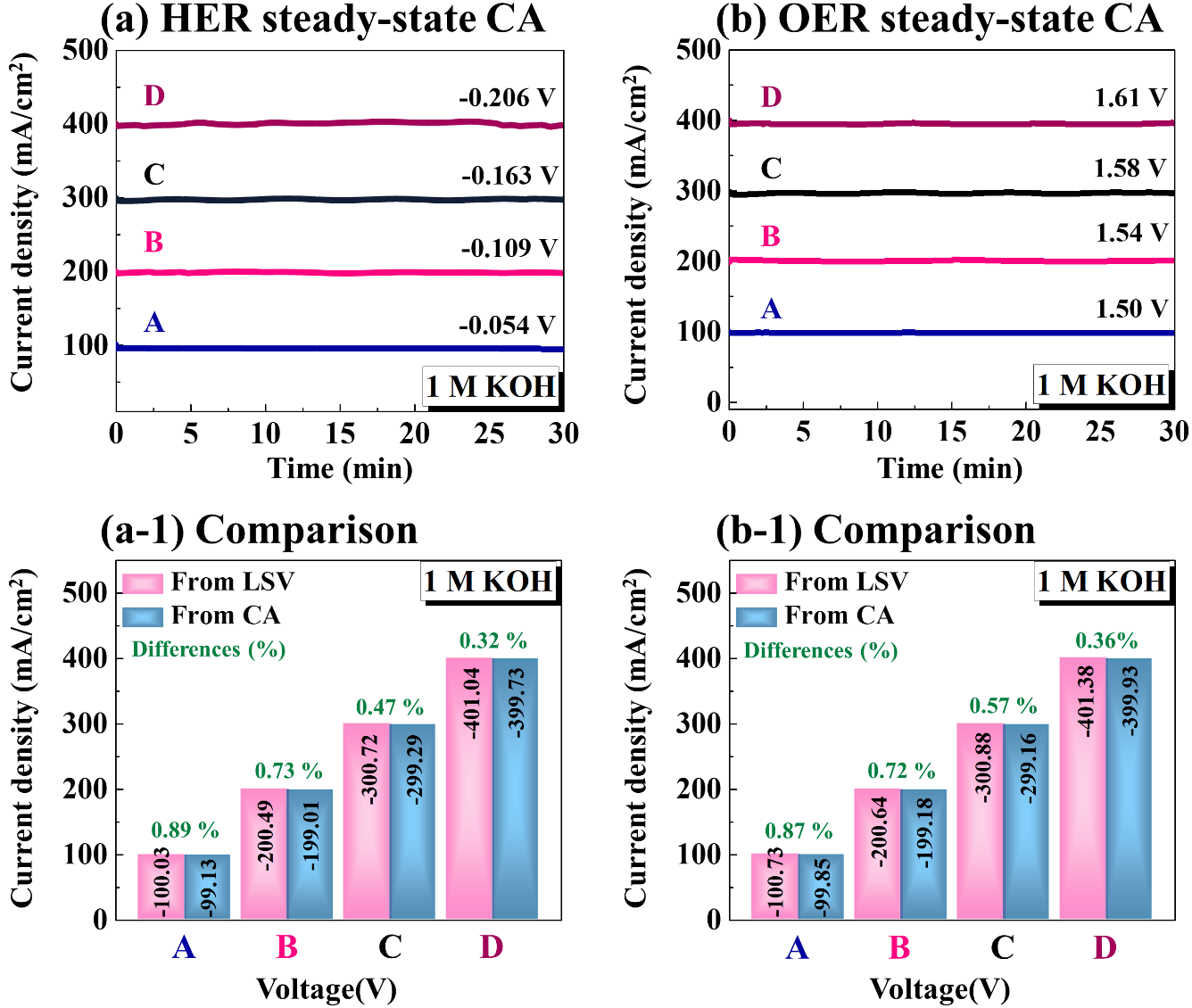
**

**Figure S30.** HER/OER chrono amperometry (CA) performance of Co/FeMoB at various applied voltages in 1 m KOH. (a–b) CA performance for HER/OER in 3-E system. (a-1–b-1) Corresponding current comparisons between LSV and CA, showing percentage differences (%) for HER/OER.

**S-3.1.6. Electrochemical: Repeatability**

**
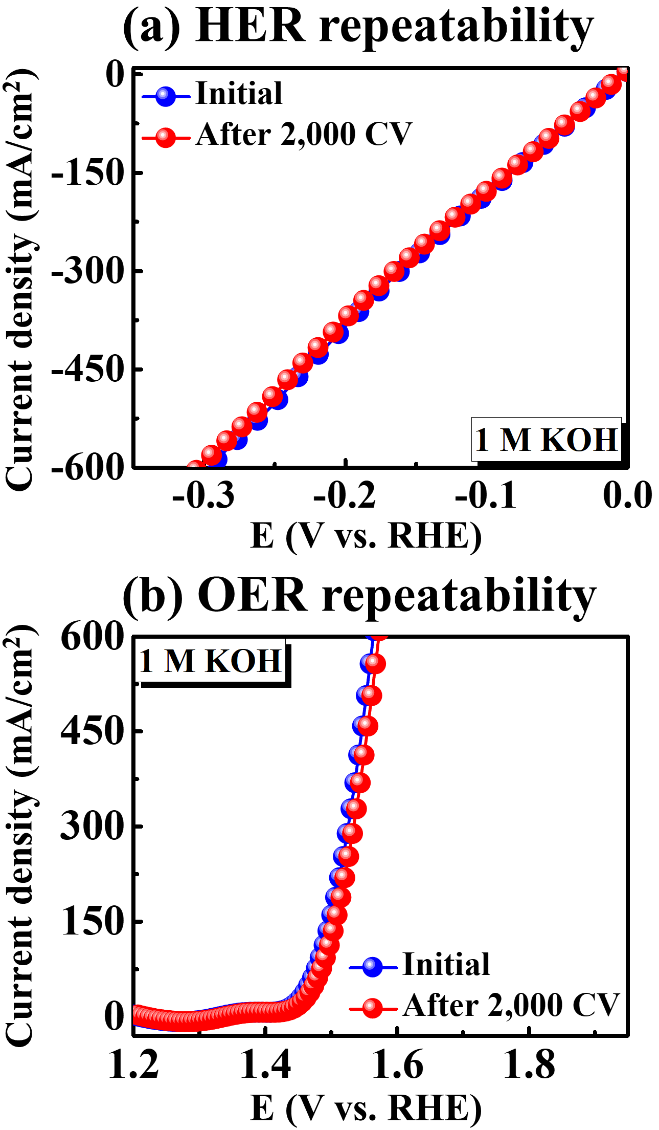
**

**Figure S31.** (a–b) HER/OER repeatability tests in 1 m KOH after continuous 2,000 CV cycles. The HER/OER LSV curves exhibited nearly identical performance before and after 2,000 cycles, indicating excellent electrochemical stability.

**S-3.1.7. Electrochemical: HER/OER stability**

**
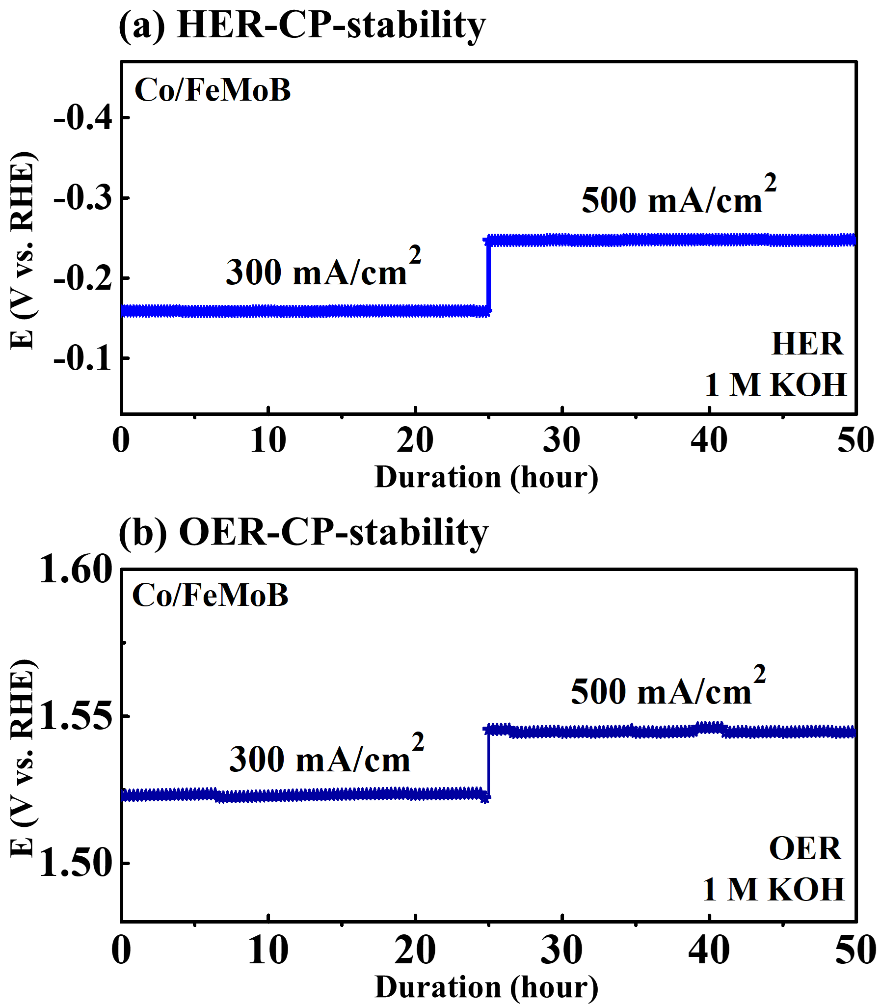
**

**Figure S32.** HER/OER chronopotentiometry (CP) stability test at different current densities in 1 m KOH. (a–b) The HER/OER dual step CP-stability tests were conducted at 300 and 500 mA/cm^2^ for total 50 hours.

**S-3.1.8. Electrochemical: Comparison NF LSV**

**
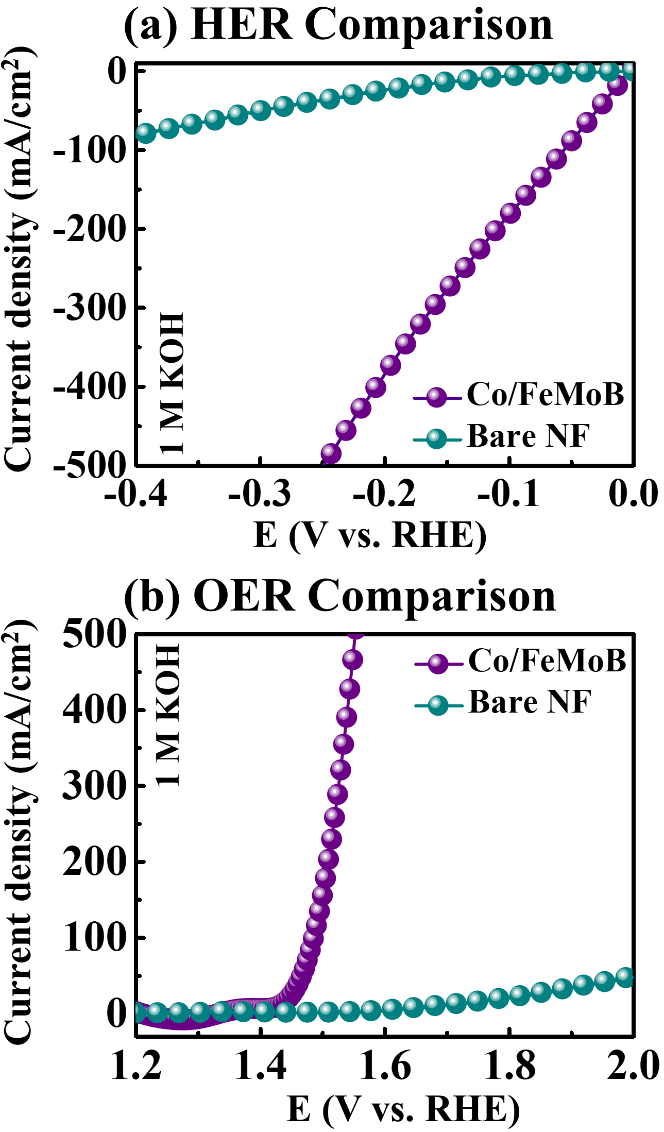
**

**Figure S33.** HER/OER performance of bare NF and Co/FeMoB electrodes in 1 m KOH. (a–b) The catalytic activity for both HER/OER is significantly enhanced following the deposition of FeMoB and subsequent Co doping on the NF substrate.

**S-3.1.9. Electrochemical: HER/OER in different pH**

**
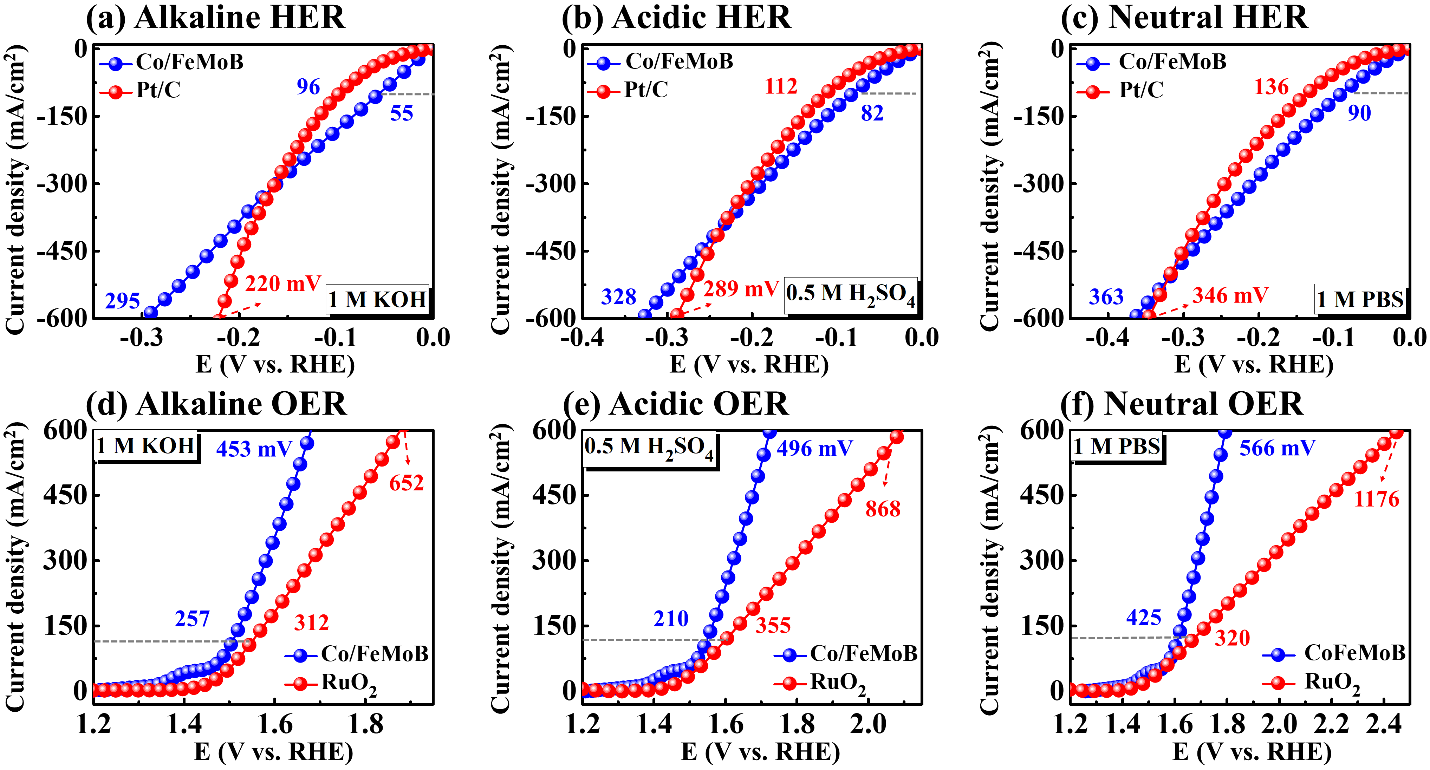
**

**Figure S34.** Three-electrode (3-E) HER/OER activity of Co/FeMoB MP electrodes. (a-c–d-f) HER/OER LSV in different pH electrolytes (alkaline, acidic and neutral) compared with benchmarks Pt/C and RuO_2_.

**S-3.1.10. Electrochemical: different pH TOF activity**

**
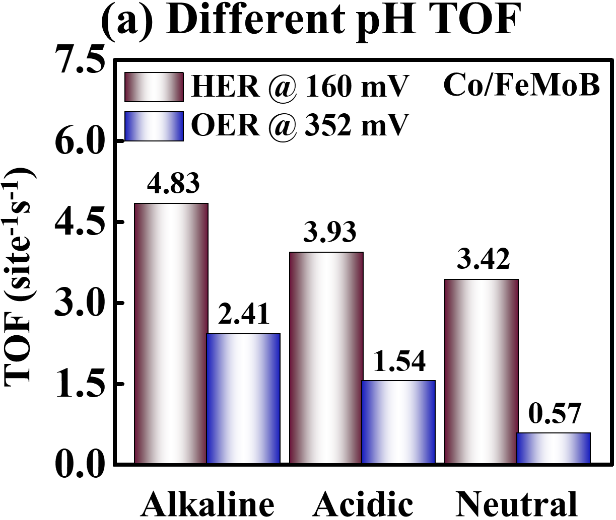
**

**Figure S35.** 3-E TOF in different pH electrolytes (alkaline, acidic and neutral) based on HER/OER LSV performances (Fig. S34). (a) HER/OER TOF in different pH solutions.

**S-3.1.11. Electrochemical: electrolyte temperature effect**

**
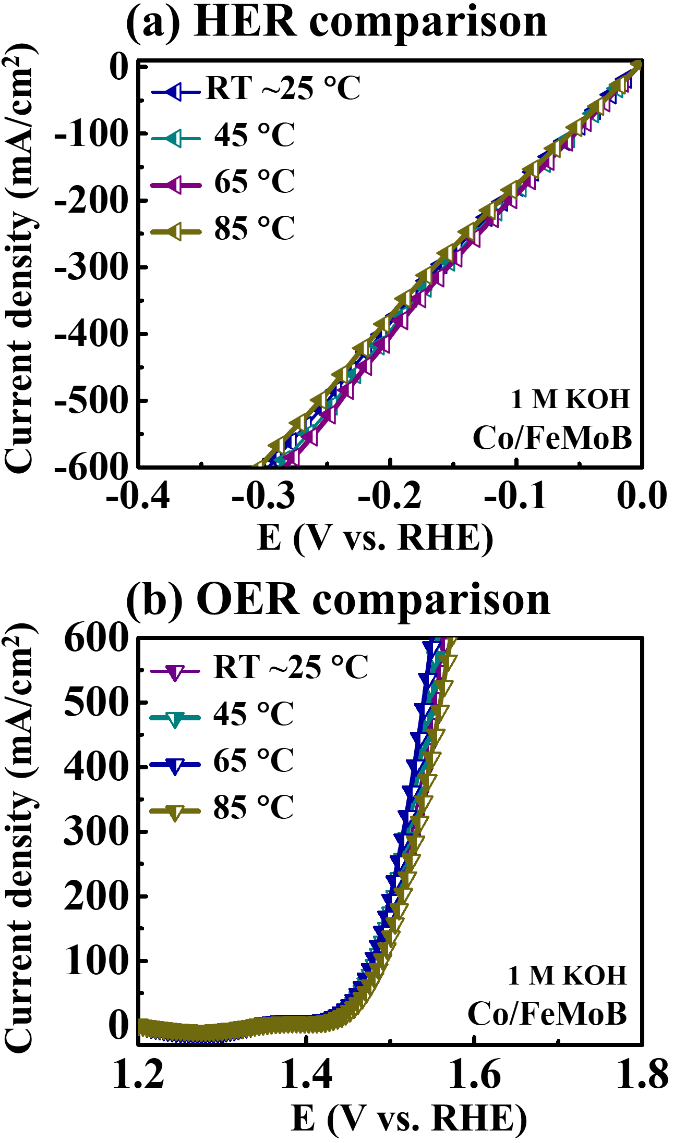
**

**Figure S36.** HER/OER performances of the best Co/FeMoB MP electrocatalyst in alkaline (1 m KOH) solution at different temperatures. The electrolyte temperatures were controlled from 25 °C (room temperature, RT) to 85 °C. (a) HER LSV curves (b) OER LSV curves.

**S-3.1.12. Electrochemical: ECSA-normalized HER/OER**

**
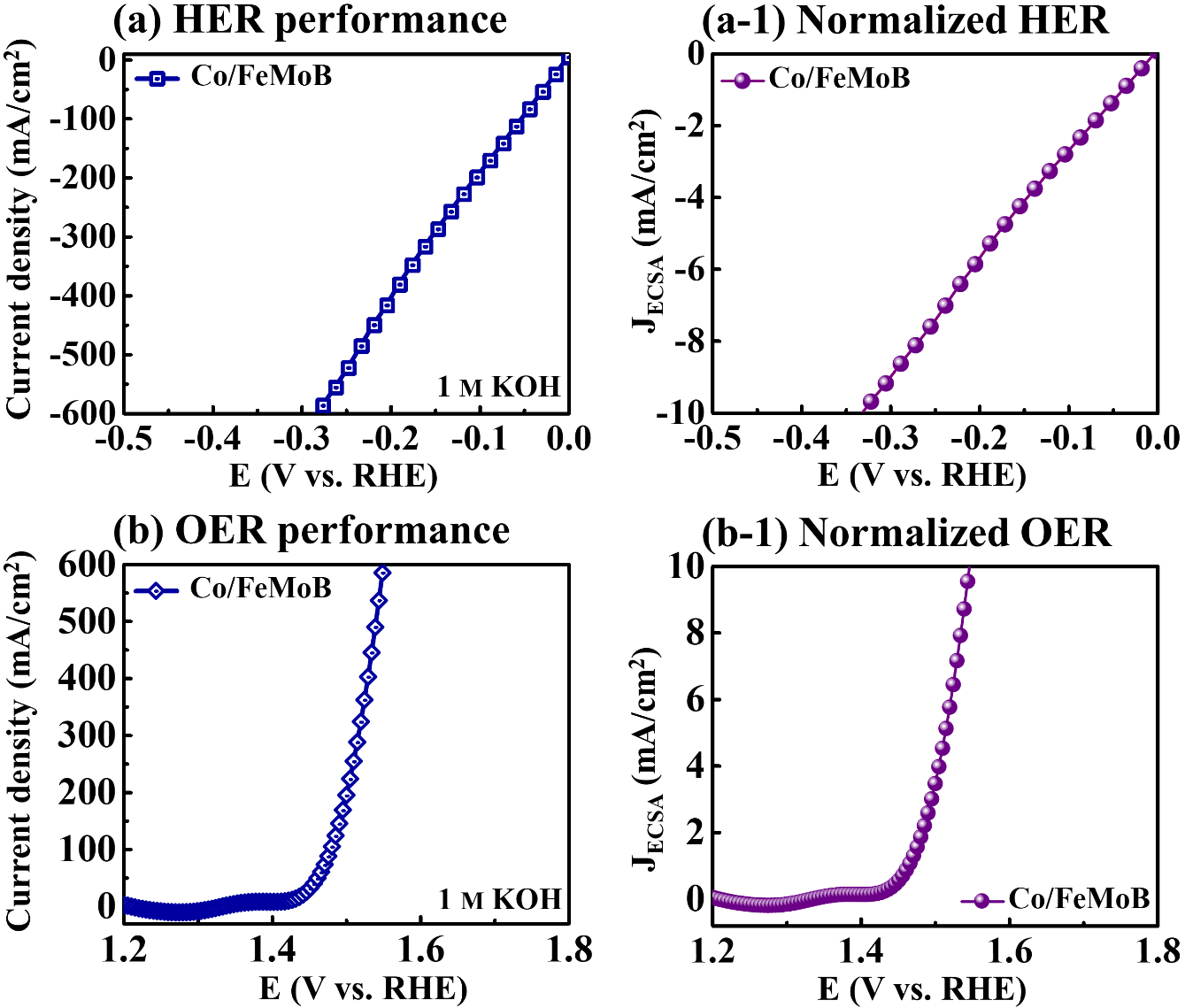
**

**Figure S37.** HER/OER performance of the 300 °C-annealed Co/FeMoB MP electrode (best-performing sample). (a–b) HER/OER polarization curves recorded in 1 m KOH solution; (a-1–b-1) Corresponding ECSA-normalized HER/OER LSV curves.

**S-3.1.13. Electrochemical: FE measurement**

**
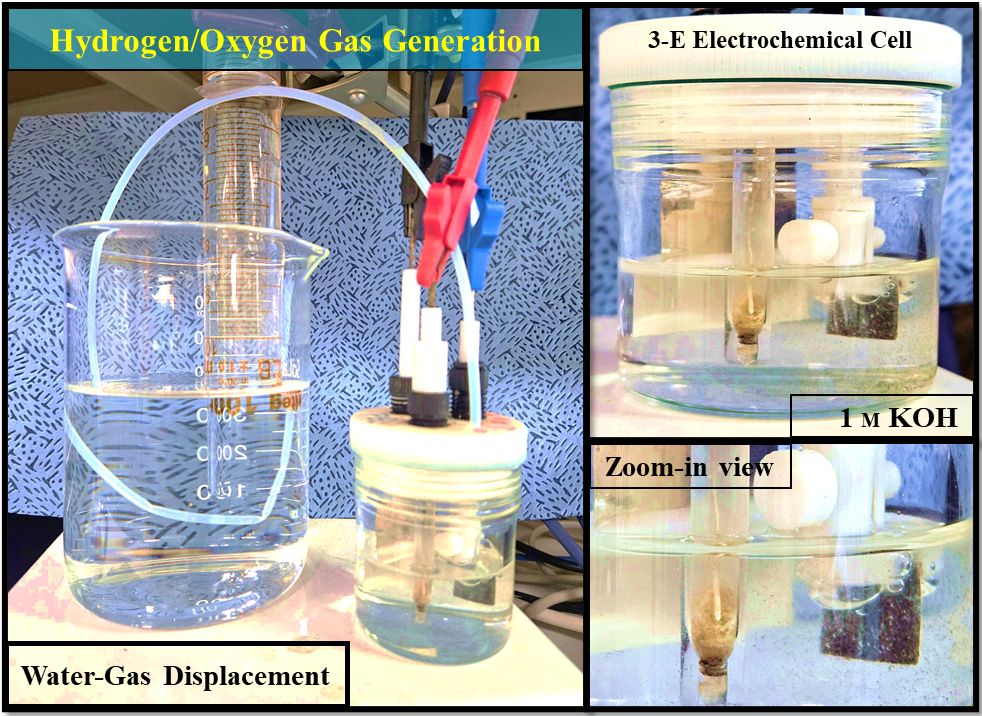
**

**Figure S38.** Digital image of the water-gas displacement setup with a zoomed-in view highlighting hydrogen/oxygen gas evolution. The enlarged image clearly shows the formation of copious gas bubbles. A more detiled discussion related to the Faradaic efficiency (FE) measurement is provided in Supplementary text (S-1.9).

**S-3.1.13. Electrochemical: FE measurement for HER/OER**

**
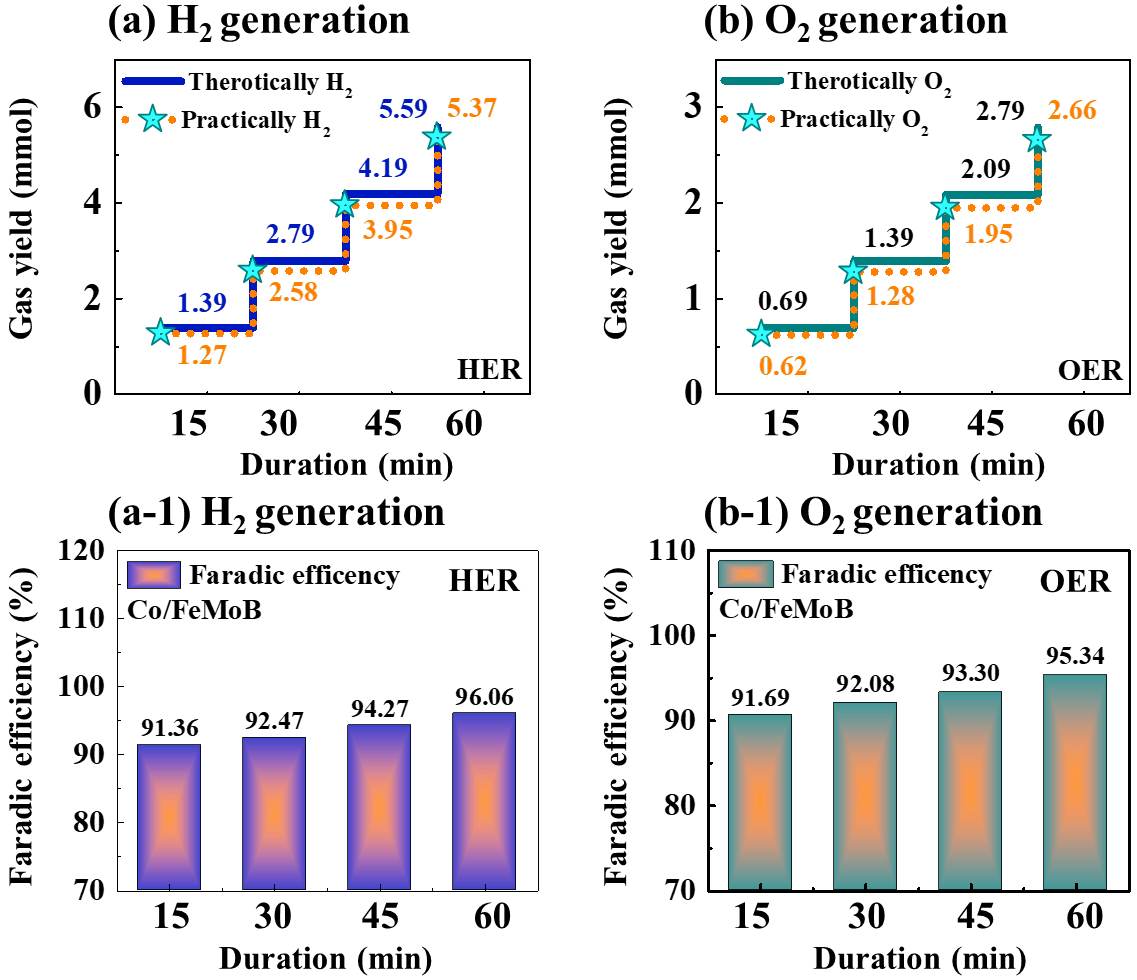
**

**Figure S39.** (a–b) Faradaic efficiency (FE) measurements for HER/OER using the Co/FeMoB electrode in 3-E setup. (a-1–b-1) FE (%) for HER/OER. Theoretical (blue line) and experimental (green line) gas yields were compared for 15 to 60 minutes of H_2_ and O_2_ generation at 300 mA/cm^2^ fixed current density.

**S-3. Analysis of the best Co/FeMoB catalyst**

**S-3.1. Physical and Electrochemical analysis**

**S-3.1.1. Physical: CS EDS**

**S-3.1.2. Physical: HR-TEM**

**S-3.1.3. Physical: Raman**

**S-3.1.4. Physical: XRD pattern**

**S-3.1.5. Electrochemical: CA/LSV**

**S-3.1.6. Electrochemical: Repeatability**

**S-3.1.7. Electrochemical: HER/OER stability**

**S-3.1.8. Electrochemical: Comparison NF LSV**

**S-3.1.9. Electrochemical: HER/OER in different pH**

**S-3.1.10. Electrochemical: different pH TOF activity**

**S-3.1.11. Electrochemical: electrolyte temperature effect**

**S-3.1.12. Electrochemical: ECSA-normalized HER/OER**

**S-3.1.13. Electrochemical: FE measurement for HER/OER**

**S-3.2. Co-doping effect on framework FeMoB catalyst**

**S-3.2.1. EDS spectra comparison**

**S-3.2.2. HER/OER EIS comparison**

**S-3.2.3. HER/OER CV curve comparison**

**S-3.2.4. HER/OER anodic/cathodic comparison**

**S-3.2.5. HER/OER overpotential comparison**

**S-3.2.6. Normalized HER/OER comparison**

**S-3.2.7. CP-stability comparison for HER/OER**

**S-3.2.8. HER/OER performance comparison**

**S-3.3. Bifunctional system of Co/FeMoB**

**S-3.3.1. Different pH operations**

**S-3.3.2. CV/LSV comparison**

**S-3.3.3. Repeatability test**

**S-3.3.4. Natural water LSV**

**S-3.3.5. Stability in NW**

**S-3.3.6. Additional CP-stability**

**S-3.4. Hybrid system of Co/FeMoB**

**S-3.4.1. Different pH operations**

**S-3.4.2. CV/LSV comparison**

**S-3.4.3. Repeatability test**

**S-3.4.4. Natural water LSV**

**S-3.5. Post-stability of Co/FeMoB**

**S-3.5.1. Micrographs of SEM**

**S-3.5.2. EDS spectra**

**S-3.5.3. Raman spectra**

**S-3.5.4. XRD pattern**

**S-3.5.5. Full-scan XPS**

**S-3.5.6. High-resolution XPS**

**S-3.5.7. 3-E HER/OER LSV**

**S-3.5.7. 2-E OWE LSV**

**S-3.2.1. EDS spectra comparison**

**
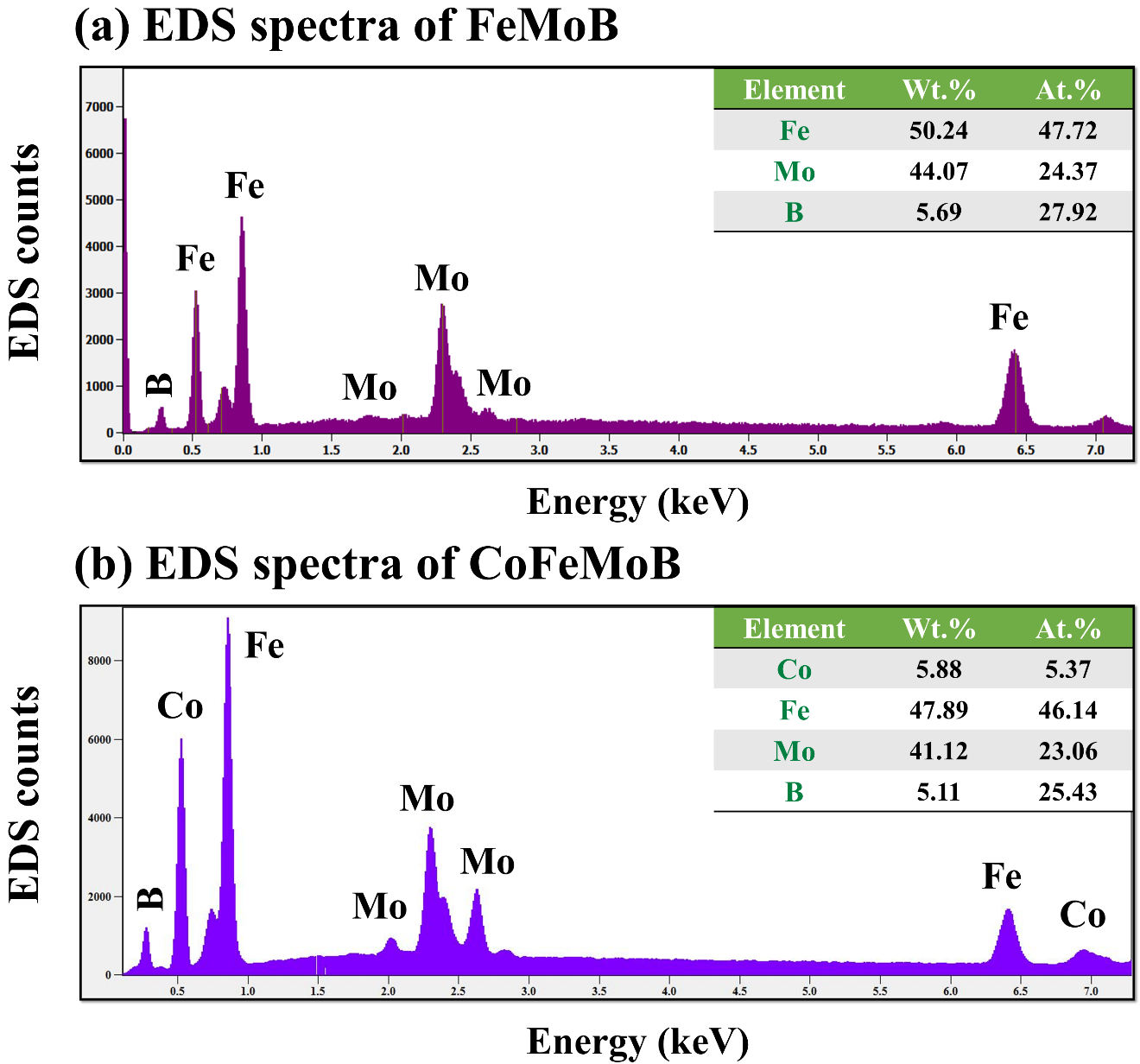
**

**Figure S40.** (a–b) EDS spectra of FeMoB and Co/FeMoB electrodes, along with summaries of elemental weight (Wt.) and atomic (At.) percentages (%).

**S-3.2.2. HER/OER EIS comparison**

**
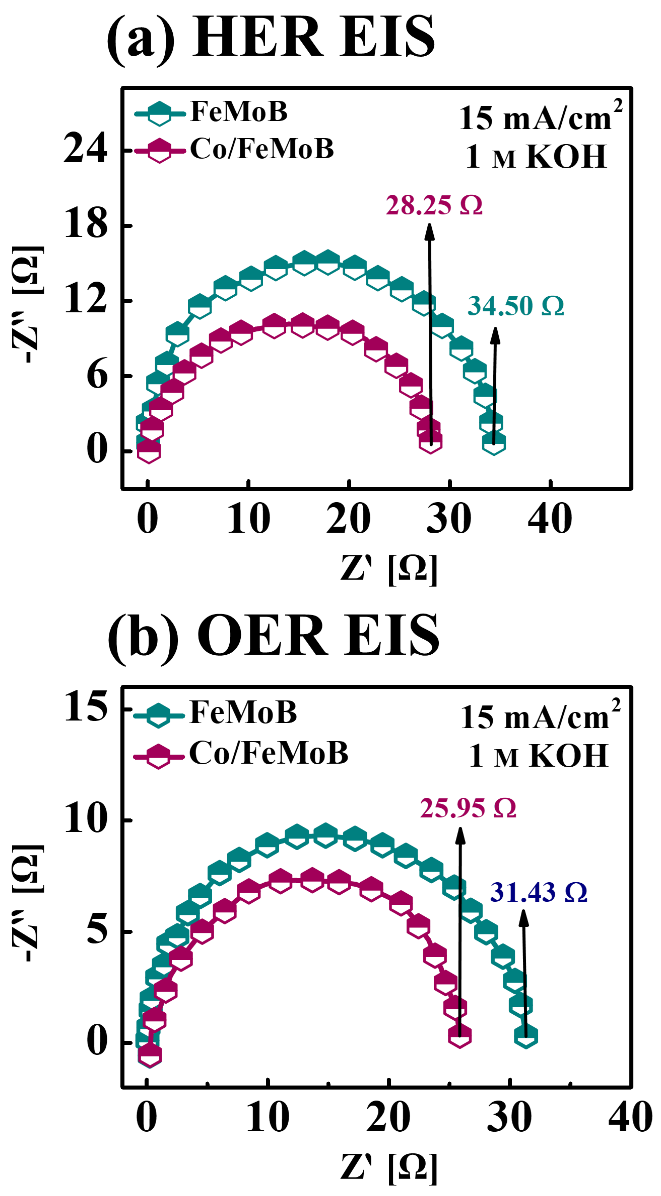
**

**Figure S41.** HER/OER EIS measurements of FeMoB and Co/FeMoB electrodes reveal that the charge transfer resistances (R_ct_) significantly after Co doping, decreasing from 31.22 to 19.87 Ω for HER and from 37.25 to 23.36 Ω for OER.

**S-3.2.3. HER/OER CV comparison**

**
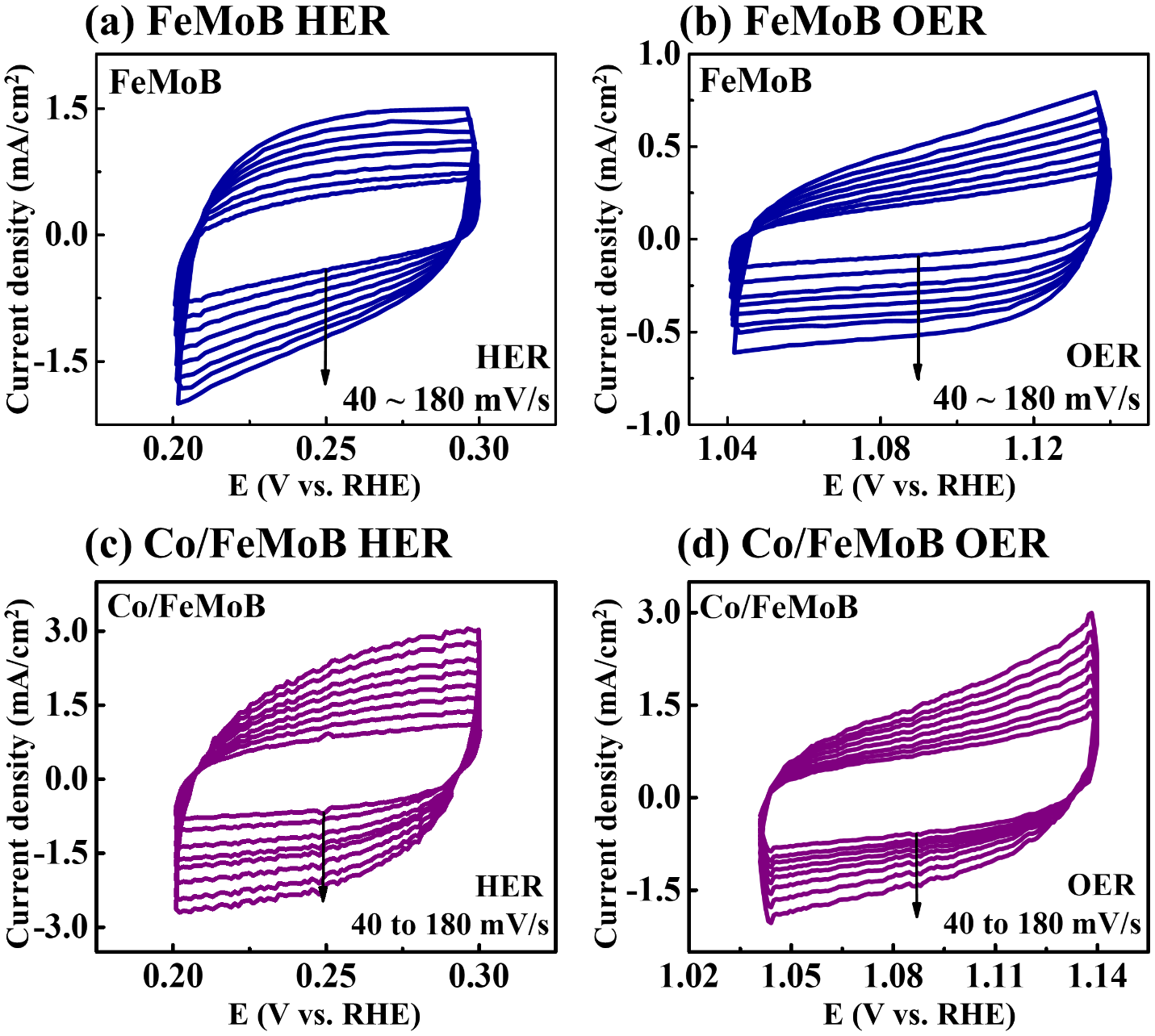
**

**Figure S42.** (a–b) and (c–d) show the HER/OER cyclic voltammetry (CV) curves of FeMoB and Co/FeMoB electrodes respectively.

**S-3.2.4. HER/OER anodic/cathodic comparison**

**
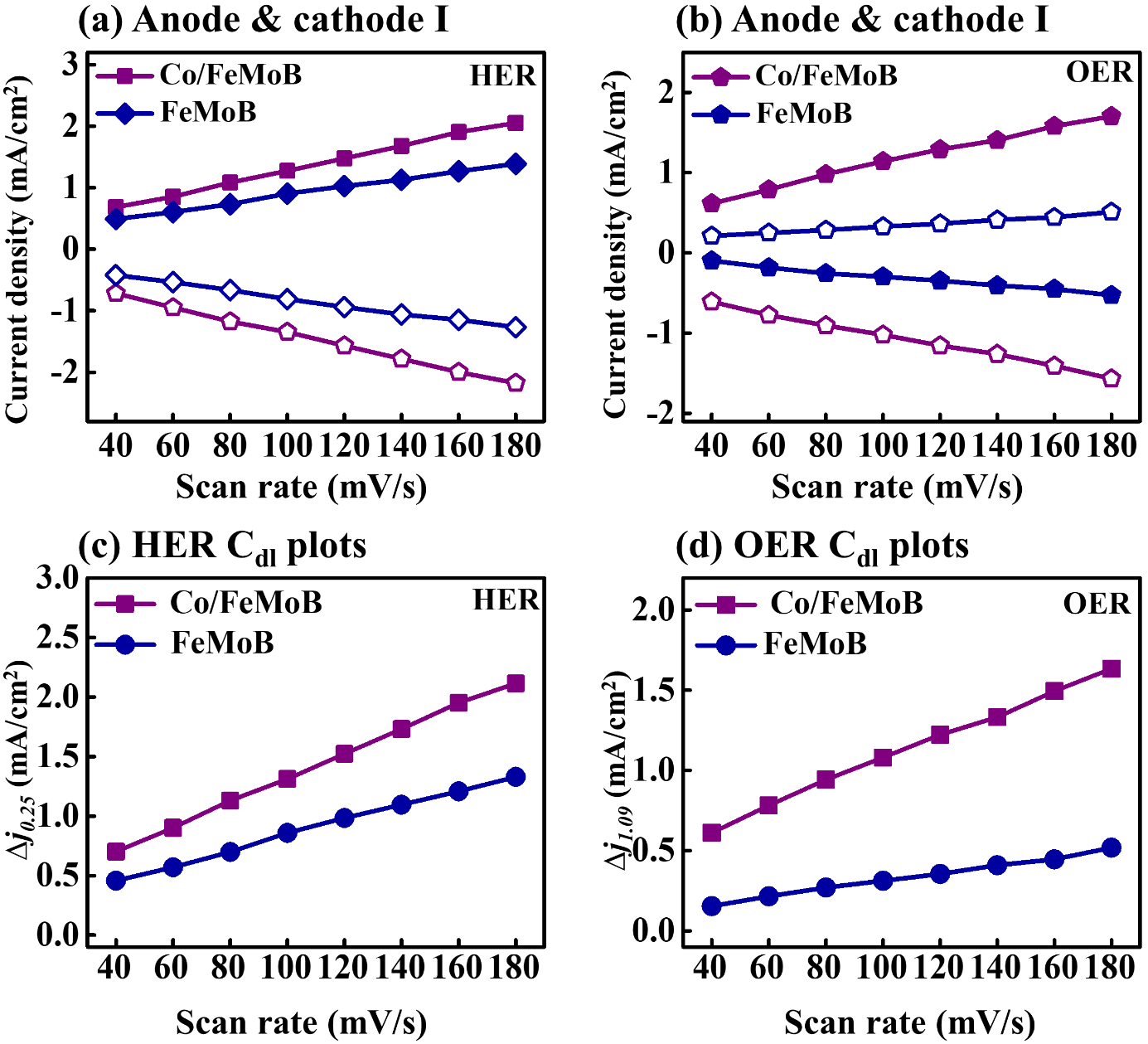
**

**Figure S43.** (a–b) HER/OER anodic and cathodic current densities of FeMoB and Co/FeMoB electrodes plotted against CV scan rates. (c–d) HER/OER current density differences (ΔJ = (Ja − Jc)/2) versus scan rates, with the corresponding slopes representing the double-layer capacitance (C_dl_) values for FeMoB and Co/FeMoB.

**S-3.2.5. HER/OER overpotential comparison**

**
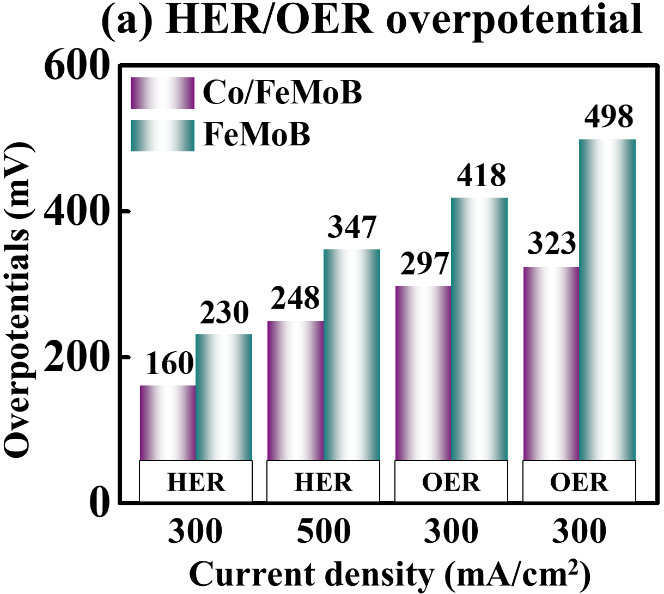
**

**Figure S44.** (a) Corresponding HER/OER overpotential bar graphs at 300 and 500 mA/cm^2^ for FeMoB and Co/FeMoB electrodes in 1 m KOH. A significant enhancement in electrocatalytic activity is observed after Co incorporation.

**S-3.2.6. Normalized HER/OER comparison**

**
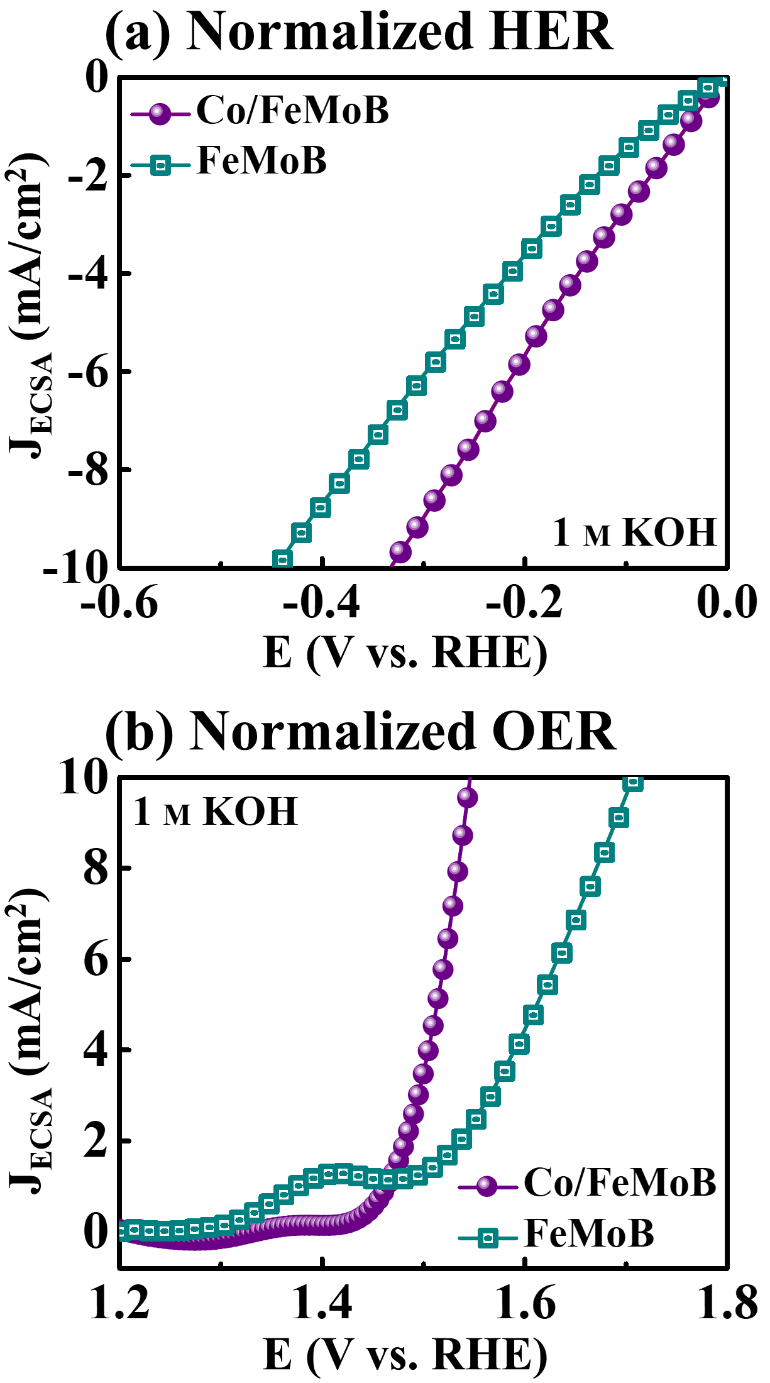
**

**Figure S45.** (a–b) HER/OER ECSA nominalized LSV curves comparision for FeMoB and Co/FeMoB electrocatalysts.

**S-3.2.7. CP-stability comparison for HER/OER**

**
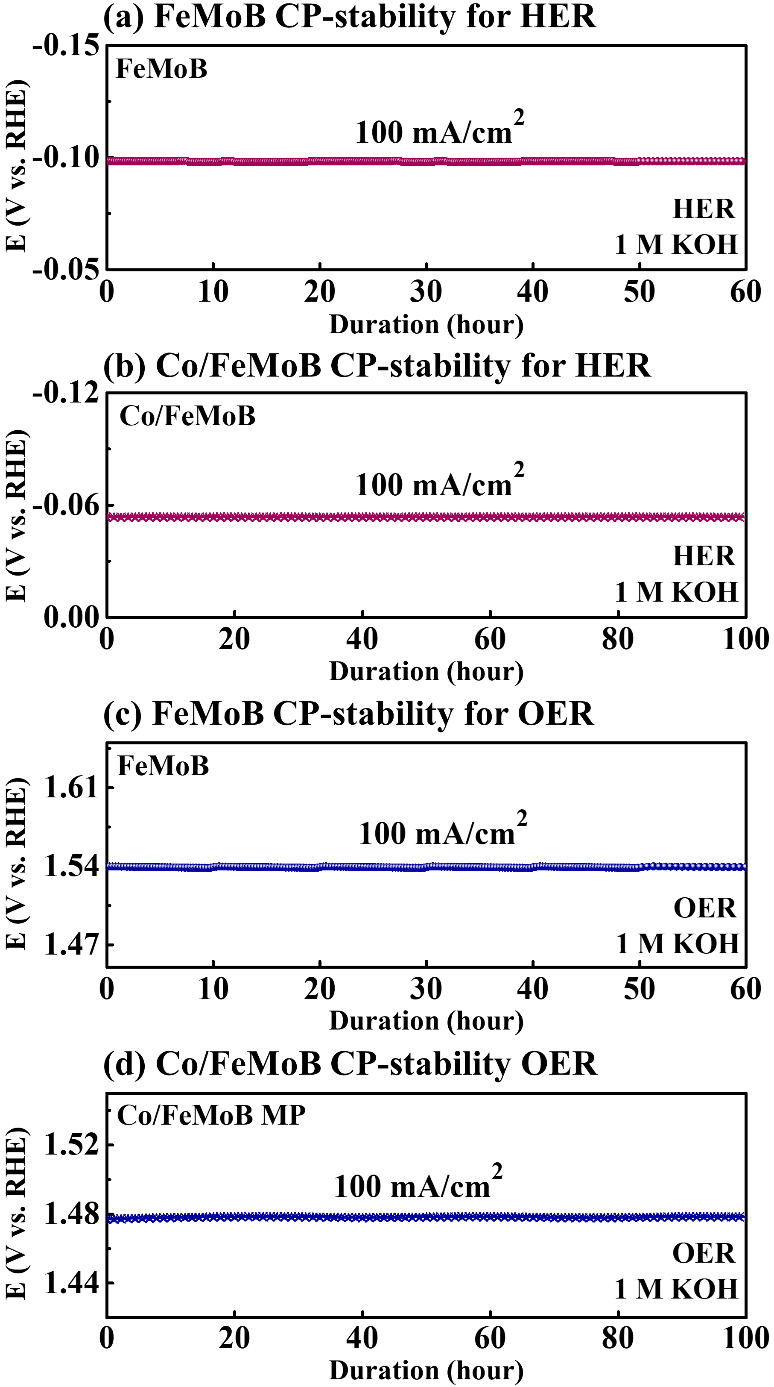
**

**Figure S46.** (a–d) HER/OER choronopotentiomentric (CP) stability oparation for FeMoB and Co/FeMoB electrodes. The stability oparation was conducted for 60 and 100 hours for in 1 m KOH under 100 mA/cm^2^ current density.

**S-3.2.8. HER/OER performance comparison**

**
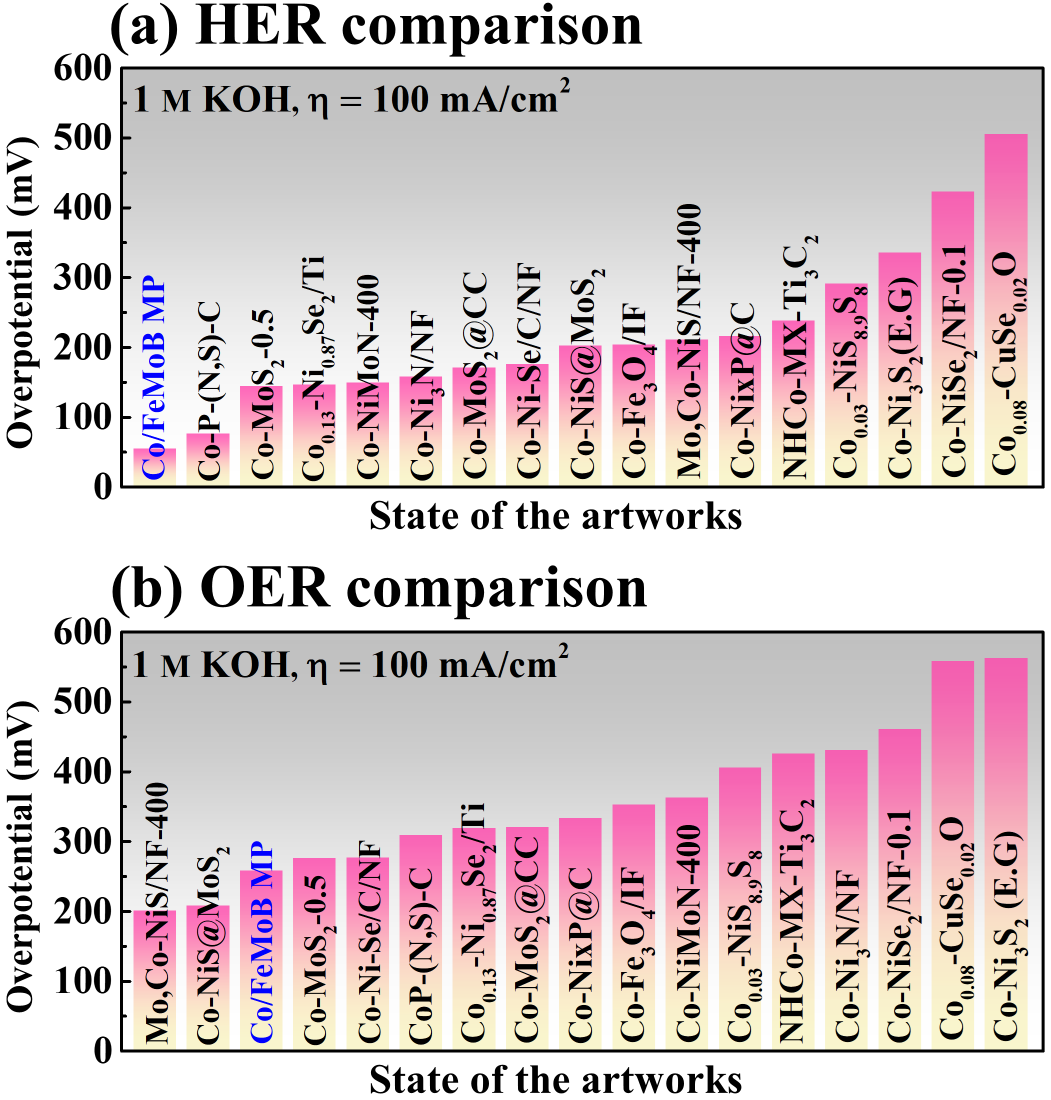
**

**Figure S47.** Comparison of Co-doped FeMoB with recently reported Co-doped transition metal (TM)-based electrocatalysts at a current density of 100 mA/cm². (a) HER performance comparison and (b) OER performance comparison.

**S-3. Analysis of the best Co/FeMoB catalyst**

**S-3.1. Physical and Electrochemical analysis**

**S-3.1.1. Physical: CS EDS**

**S-3.1.2. Physical: HR-TEM**

**S-3.1.3. Physical: Raman**

**S-3.1.4. Physical: XRD pattern**

**S-3.1.5. Electrochemical: CA/LSV**

**S-3.1.6. Electrochemical: Repeatability**

**S-3.1.7. Electrochemical: HER/OER stability**

**S-3.1.8. Electrochemical: Comparison NF LSV**

**S-3.1.9. Electrochemical: HER/OER in different pH**

**S-3.1.10. Electrochemical: different pH TOF activity**

**S-3.1.11. Electrochemical: electrolyte temperature effect**

**S-3.1.12. Electrochemical: ECSA-normalized HER/OER**

**S-3.1.13. Electrochemical: FE measurement for HER/OER**

**S-3.2. Co-doping effect on framework FeMoB catalyst**

**S-3.2.1. EDS spectra comparison**

**S-3.2.2. HER/OER EIS comparison**

**S-3.2.3. HER/OER CV curve comparison**

**S-3.2.4. HER/OER anodic/cathodic comparison**

**S-3.2.5. HER/OER overpotential comparison**

**S-3.2.6. Normalized HER/OER comparison**

**S-3.2.7. CP-stability comparison for HER/OER**

**S-3.2.8. HER/OER performance comparison**

**S-3.3. Bifunctional system of Co/FeMoB**

**S-3.3.1. Different pH operations**

**S-3.3.2. CV/LSV comparison**

**S-3.3.3. Repeatability test**

**S-3.3.4. Natural water LSV**

**S-3.3.5. Stability in NW**

**S-3.3.6. Additional CP-stability**

**S-3.4. Hybrid system of Co/FeMoB**

**S-3.4.1. Different pH operations**

**S-3.4.2. CV/LSV comparison**

**S-3.4.3. Repeatability test**

**S-3.4.4. Natural water LSV**

**S-3.5. Post-stability of Co/FeMoB**

**S-3.5.1. Micrographs of SEM**

**S-3.5.2. EDS spectra**

**S-3.5.3. Raman spectra**

**S-3.5.4. XRD pattern**

**S-3.5.5. Full-scan XPS**

**S-3.5.6. High-resolution XPS**

**S-3.5.7. 3-E HER/OER LSV**

**S-3.5.7. 2-E OWE LSV**

**S-3.3.1. Different pH operations**

**
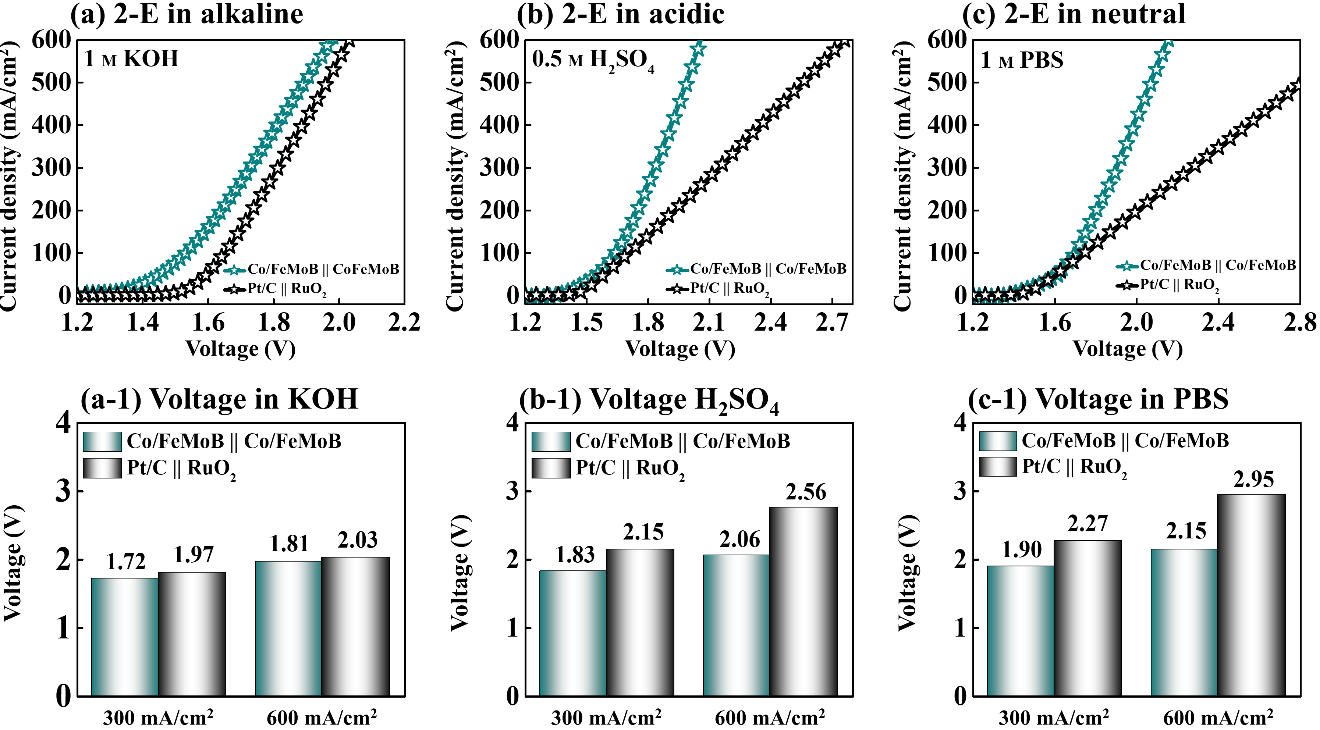
**

**Figure S48.** 2-electrode (2-E) bifunctional configuration Co/FeMoB (– ̸̸̸ ̸ +) LSV performance compared with benchmark electrodes across various pH environments. (a–c) 2-E performances in alkaline, acidic and neutral media. (a-1–c-1) Corresponding overpotential summaries at current densities of 150 and 500 mA/cm^2^.

**S-3.3.2. CV/LSV current comparison**

**
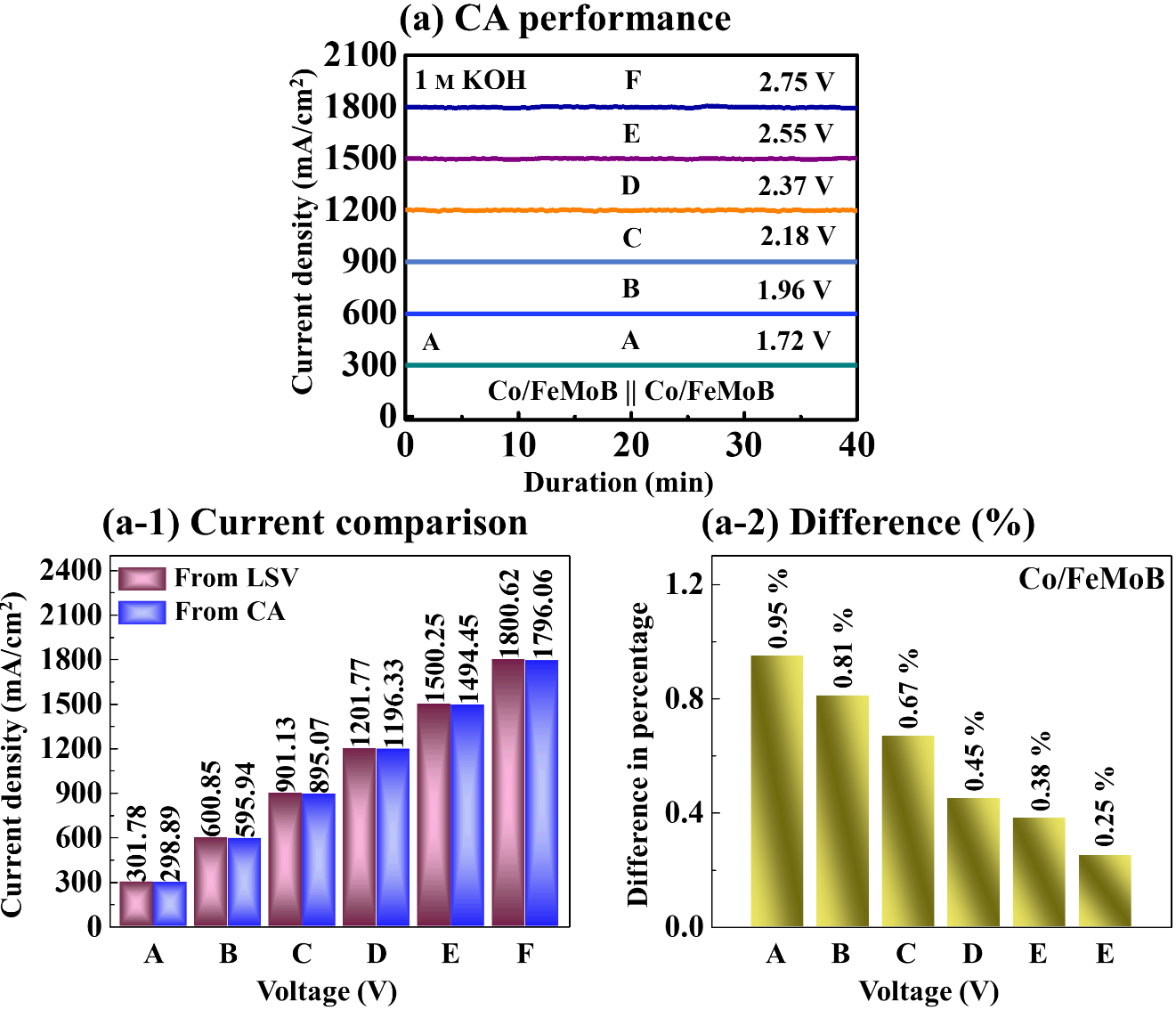
**

**Figure S49.** (a) 2-E steady-state chronoamperometry (CA) measurements of bifuctional Co/FeMoB at various applied voltages in 1  m KOH. (a-1) Comparison of current densities obtained from LSV and CA. (a-2) Corresponding percentag (%) differences between LSV and CA results.

**S-3.3.3. Repeatability test**

**
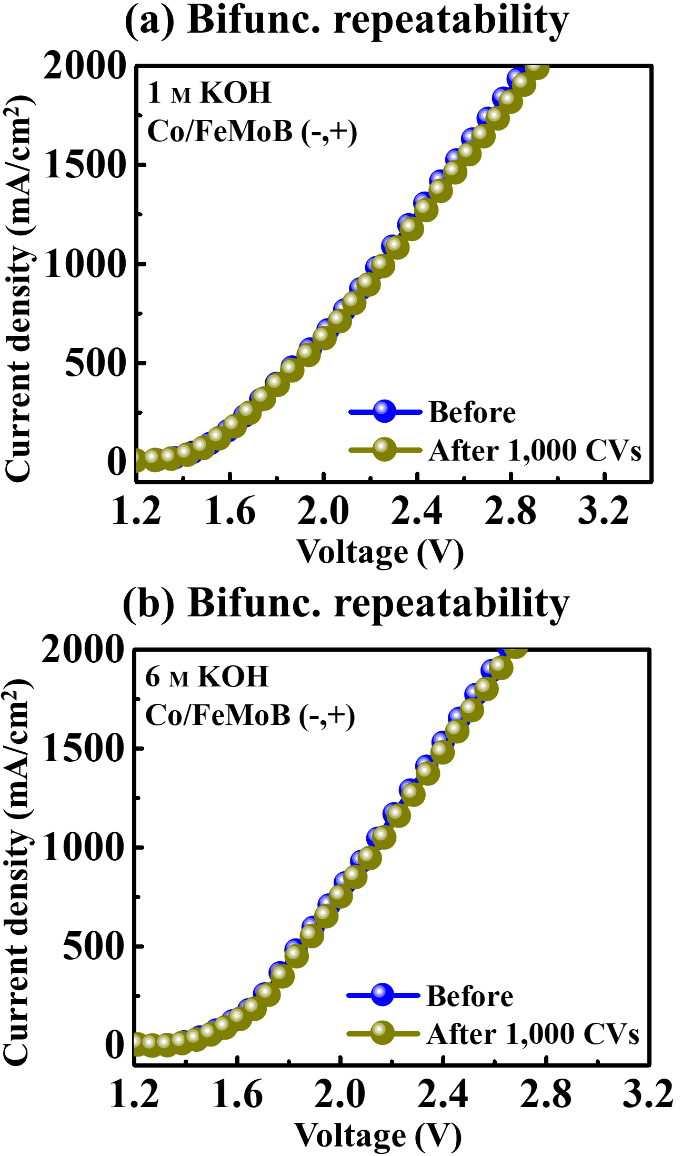
**

**Figure S50.** Repeatability test of the 2-E bifunctional Co/FeMoB (– ̸̸̸ ̸ +) system after 1,500 CV cycles for 12 hours (a–b) Post-cycling performance evaluation in 1 and 6 m KOH solution.

**S-3.3.4. Natural water LSV**

**
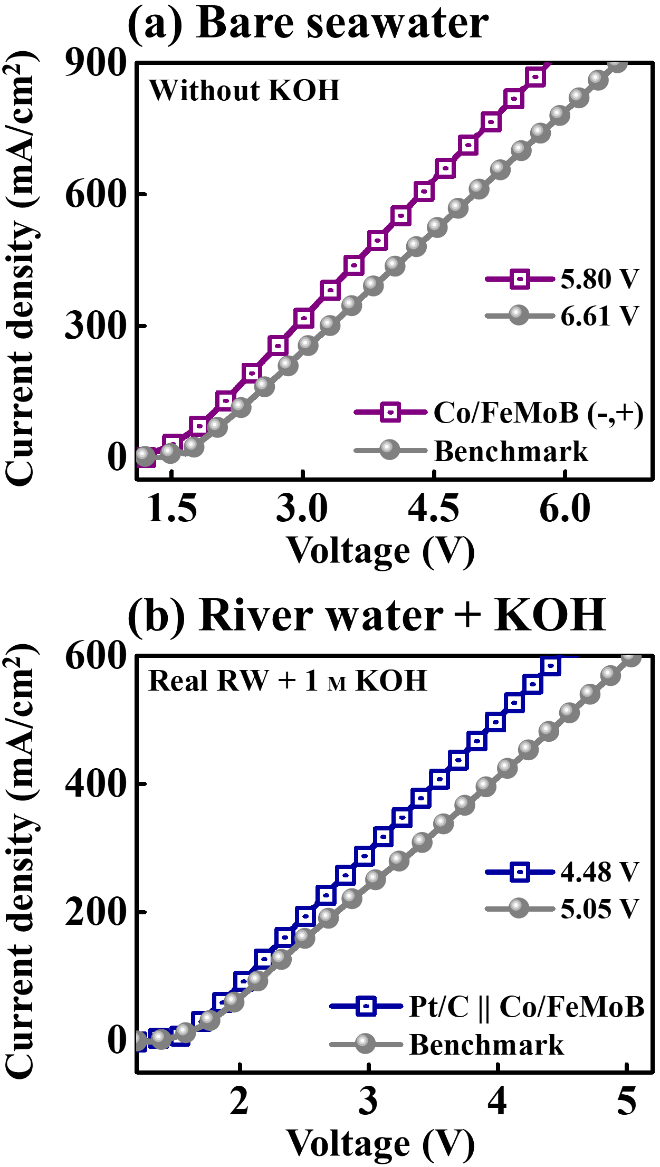
**

**Figure S51.** Bifunctional 2-E activity in fresh seawater (SW) and alkaline river water (RW + 1  m KOH). (a) Performance comparison of the Co/FeMoB (– ̸̸̸ ̸ +) and benchmark Pt/C || RuO_2_ systems in bare seawater. (b) 2-E LSV curves of the bifunctional system compared with the benchmarks in RW + 1  m KOH, where the Co/FeMoB (– ̸̸̸ ̸ +) exhibits a lower overpotential under both conditions.

**S-3.3.5. Stability in NW**

**
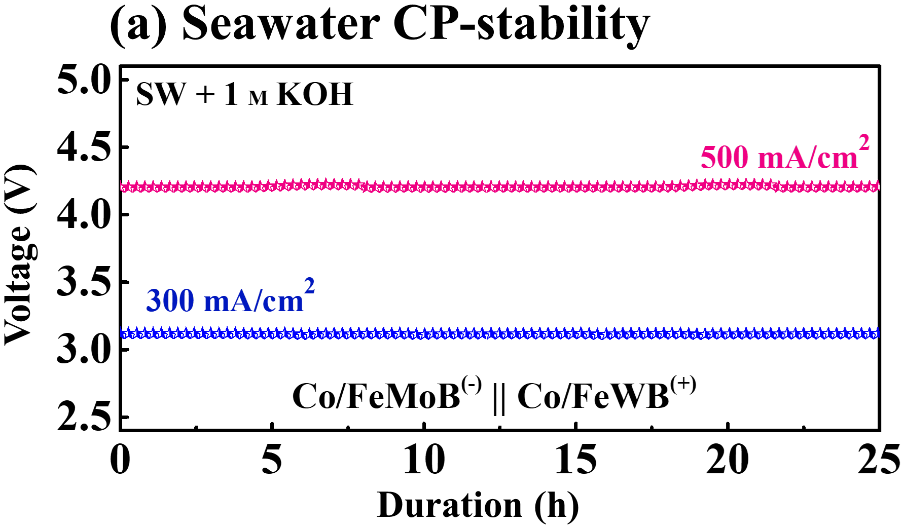
**

**Figure S52.** 2-E bifunctional Co/FeMoB (– ̸̸̸ ̸ +) chronopotentiometric (CP) stability test in alkaline seawater (SW + 1 m KOH). (a) Stability oparation over 25 hours at current densities of 300 and 500 mA/cm^2^.

**S-3.3.6. Additional CP-stability**

**
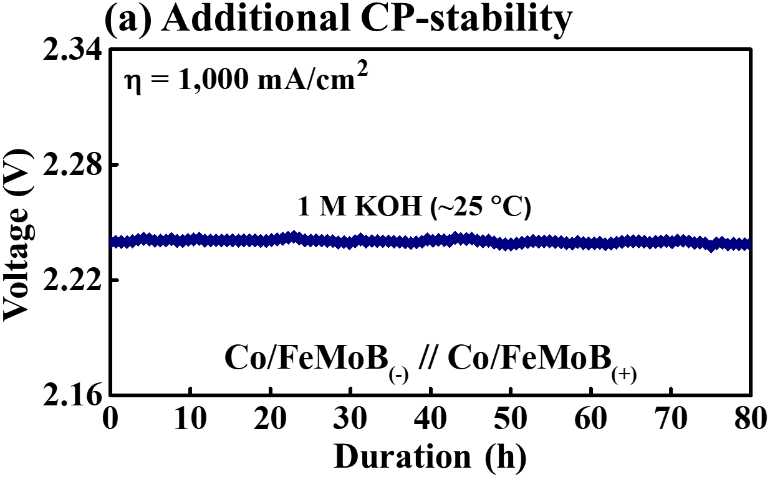
**

**Figure S53.** 2-E additional bifunctional Co/FeMoB (– ̸̸̸ ̸ +) chronopotentiometric (CP) stability test in alkaline media. (a) Stability oparation over 80 hours at 1,000 mA/cm^2^ current density.

**S-3. Analysis of the best Co/FeMoB catalyst**

**S-3.1. Physical and Electrochemical analysis**

**S-3.1.1. Physical: CS EDS**

**S-3.1.2. Physical: HR-TEM**

**S-3.1.3. Physical: Raman**

**S-3.1.4. Physical: XRD pattern**

**S-3.1.5. Electrochemical: CA/LSV**

**S-3.1.6. Electrochemical: Repeatability**

**S-3.1.7. Electrochemical: HER/OER stability**

**S-3.1.8. Electrochemical: Comparison NF LSV**

**S-3.1.9. Electrochemical: HER/OER in different pH**

**S-3.1.10. Electrochemical: different pH TOF activity**

**S-3.1.11. Electrochemical: electrolyte temperature effect**

**S-3.1.12. Electrochemical: ECSA-normalized HER/OER**

**S-3.1.13. Electrochemical: FE measurement for HER/OER**

**S-3.2. Co-doping effect on framework FeMoB catalyst**

**S-3.2.1. EDS spectra comparison**

**S-3.2.2. HER/OER EIS comparison**

**S-3.2.3. HER/OER CV curve comparison**

**S-3.2.4. HER/OER anodic/cathodic comparison**

**S-3.2.5. HER/OER overpotential comparison**

**S-3.2.6. Normalized HER/OER comparison**

**S-3.2.7. CP-stability comparison for HER/OER**

**S-3.2.8. HER/OER performance comparison**

**S-3.3. Bifunctional system of Co/FeMoB**

**S-3.3.1. Different pH operations**

**S-3.3.2. CV/LSV comparison**

**S-3.3.3. Repeatability test**

**S-3.3.4. Natural water LSV**

**S-3.3.5. Stability in NW**

**S-3.3.6. Additional CP-stability**

**S-3.4. Hybrid system of Co/FeMoB**

**S-3.4.1. Different pH operations**

**S-3.4.2. CV/LSV comparison**

**S-3.4.3. Repeatability test**

**S-3.4.4. Natural water LSV**

**S-3.5. Post-stability of Co/FeMoB**

**S-3.5.1. Micrographs of SEM**

**S-3.5.2. EDS spectra**

**S-3.5.3. Raman spectra**

**S-3.5.4. XRD pattern**

**S-3.5.5. Full-scan XPS**

**S-3.5.6. High-resolution XPS**

**S-3.5.7. 3-E HER/OER LSV**

**S-3.5.7. 2-E OWE LSV**

**S-3.4.1. Different pH operations**

**
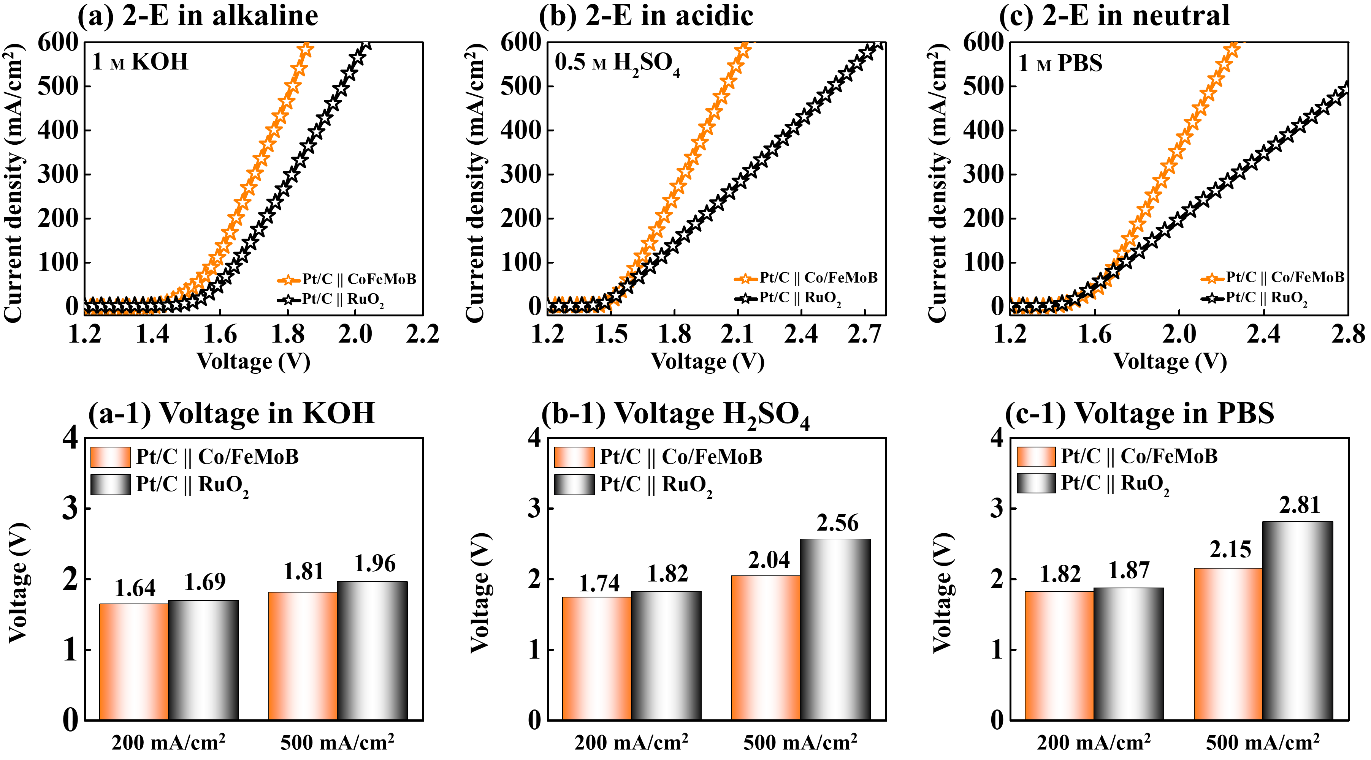
**

**Figure S54.** 2-E hybrid system Pt/C (−) || Co/FeMoB (+) LSV performance in various pH environments, compared with a reference electrode. (a–c) 2-E performance in 1  m KOH, 0.5 _M_ H_2_SO_4_ and 1  m PBS, respectively. (a-1–c-1) Corresponding overpotentials at current densities of 200 and 500 mA/cm^2^.

**S-3.4.2. CV/LSV comparison**

**
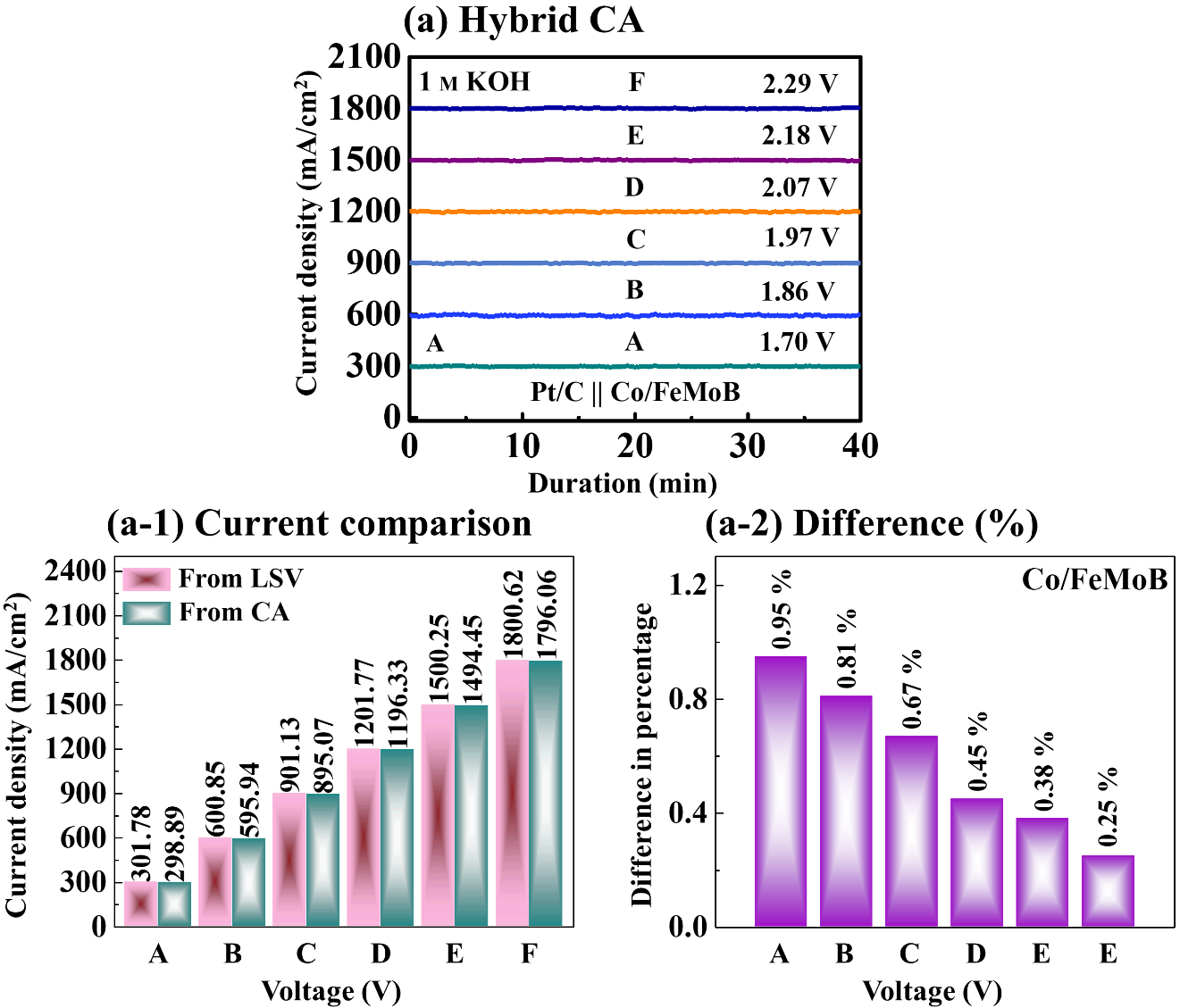
**

**Figure S55.** 2-E steady-state evaluation of the hybrid Pt/C (−) || Co/FeMoB (+) system using LSV and CA in 1  m KOH. (a) CA measurements at different applied voltages. (a-1) Comparison of current densities obtained from LSV and CA. (a-2) Corresponding % differences between LSV and CA results, indicating performance consistency under steady-state and dynamic conditions.

**S-3.4.3. Repeatability test**

**
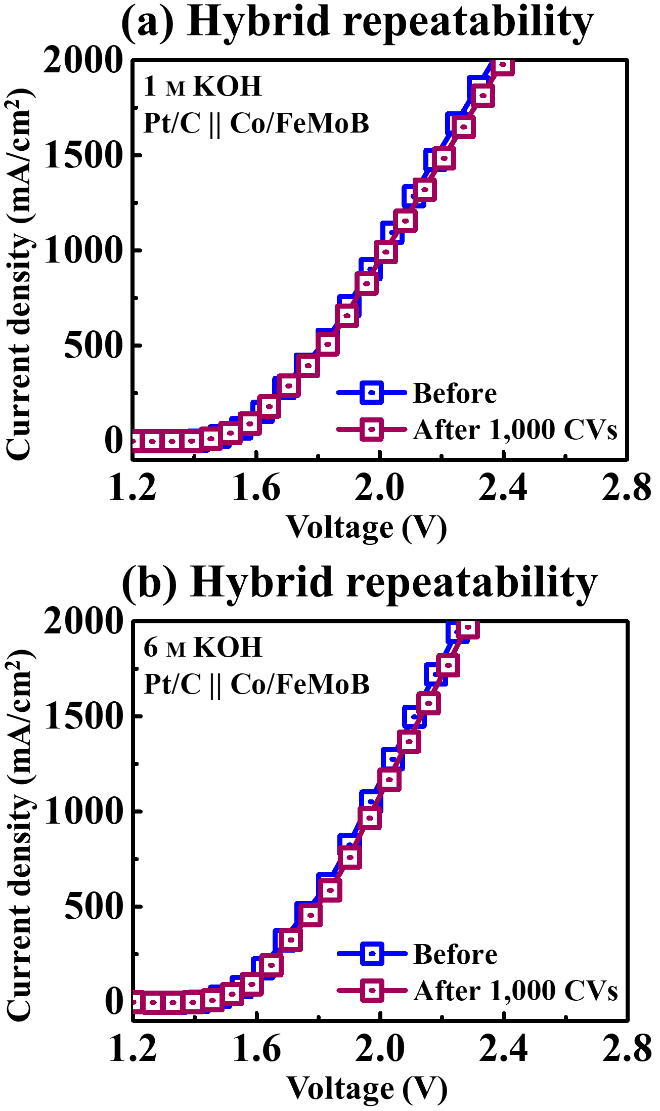
**

**Figure S56.** Repeatability test of the hybrid Pt/C (−) || Co/FeMoB (+) system after 1,500 CV cycles. (a) Post-cycling performance evaluation in 1  m KOH solution.

**S-3.4.4. Natural water LSV**

**
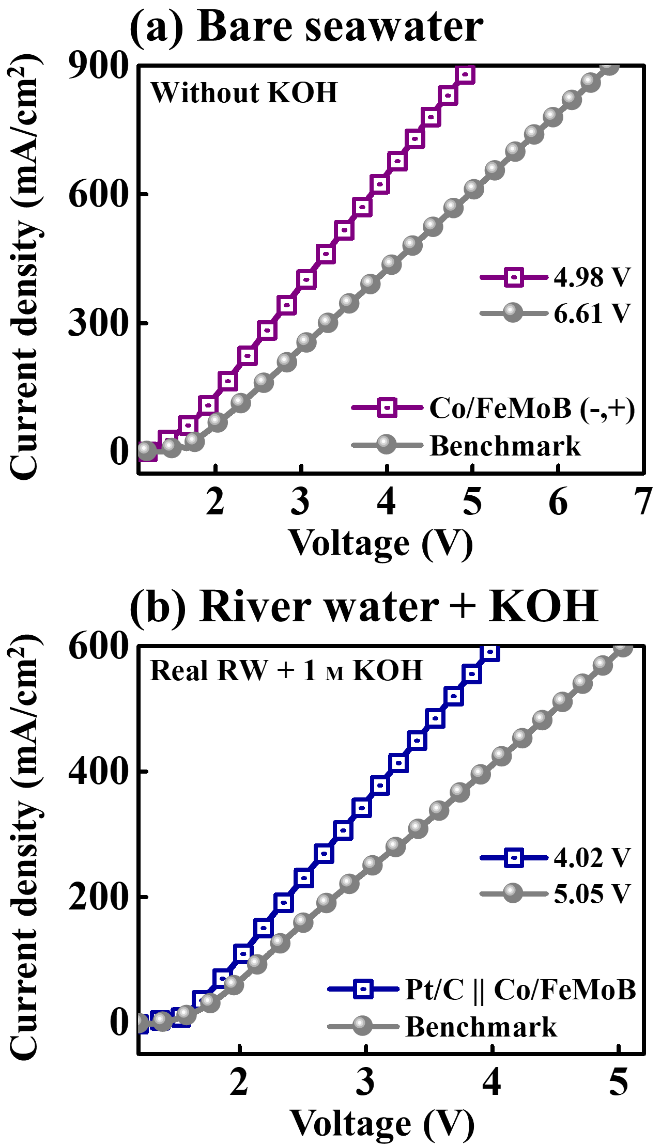
**

**Figure S57.** (a) 2-E hybrid Pt/C (−) || Co/FeMoB (+) setup activity in bare seawater (SW) without adding any chemicals. (b) Hybrid performance in river water (RW) with 1 m KOH.

**S-3. Analysis of the best Co/FeMoB catalyst**

**S-3.1. Physical and Electrochemical analysis**

**S-3.1.1. Physical: CS EDS**

**S-3.1.2. Physical: HR-TEM**

**S-3.1.3. Physical: Raman**

**S-3.1.4. Physical: XRD pattern**

**S-3.1.5. Electrochemical: CA/LSV**

**S-3.1.6. Electrochemical: Repeatability**

**S-3.1.7. Electrochemical: HER/OER stability**

**S-3.1.8. Electrochemical: Comparison NF LSV**

**S-3.1.9. Electrochemical: HER/OER in different pH**

**S-3.1.10. Electrochemical: different pH TOF activity**

**S-3.1.11. Electrochemical: electrolyte temperature effect**

**S-3.1.12. Electrochemical: ECSA-normalized HER/OER**

**S-3.1.13. Electrochemical: FE measurement for HER/OER**

**S-3.2. Co-doping effect on framework FeMoB catalyst**

**S-3.2.1. EDS spectra comparison**

**S-3.2.2. HER/OER EIS comparison**

**S-3.2.3. HER/OER CV curve comparison**

**S-3.2.4. HER/OER anodic/cathodic comparison**

**S-3.2.5. HER/OER overpotential comparison**

**S-3.2.6. Normalized HER/OER comparison**

**S-3.2.7. CP-stability comparison for HER/OER**

**S-3.2.8. HER/OER performance comparison**

**S-3.3. Bifunctional system of Co/FeMoB**

**S-3.3.1. Different pH operations**

**S-3.3.2. CV/LSV comparison**

**S-3.3.3. Repeatability test**

**S-3.3.4. Natural water LSV**

**S-3.3.5. Stability in NW**

**S-3.3.6. Additional CP-stability**

**S-3.4. Hybrid system of Co/FeMoB**

**S-3.4.1. Different pH operations**

**S-3.4.2. CV/LSV comparison**

**S-3.4.3. Repeatability test**

**S-3.4.4. Natural water LSV**

**S-3.5. Post-stability of Co/FeMoB**

**S-3.5.1. Micrographs of SEM**

**S-3.5.2. EDS spectra**

**S-3.5.3. Raman spectra**

**S-3.5.4. XRD pattern**

**S-3.5.5. Full-scan XPS**

**S-3.5.6. High-resolution XPS**

**S-3.5.7. 3-E HER/OER LSV**

**S-3.5.7. 2-E OWE LSV**

**S-3.5.1. Micrographs of SEM**

**
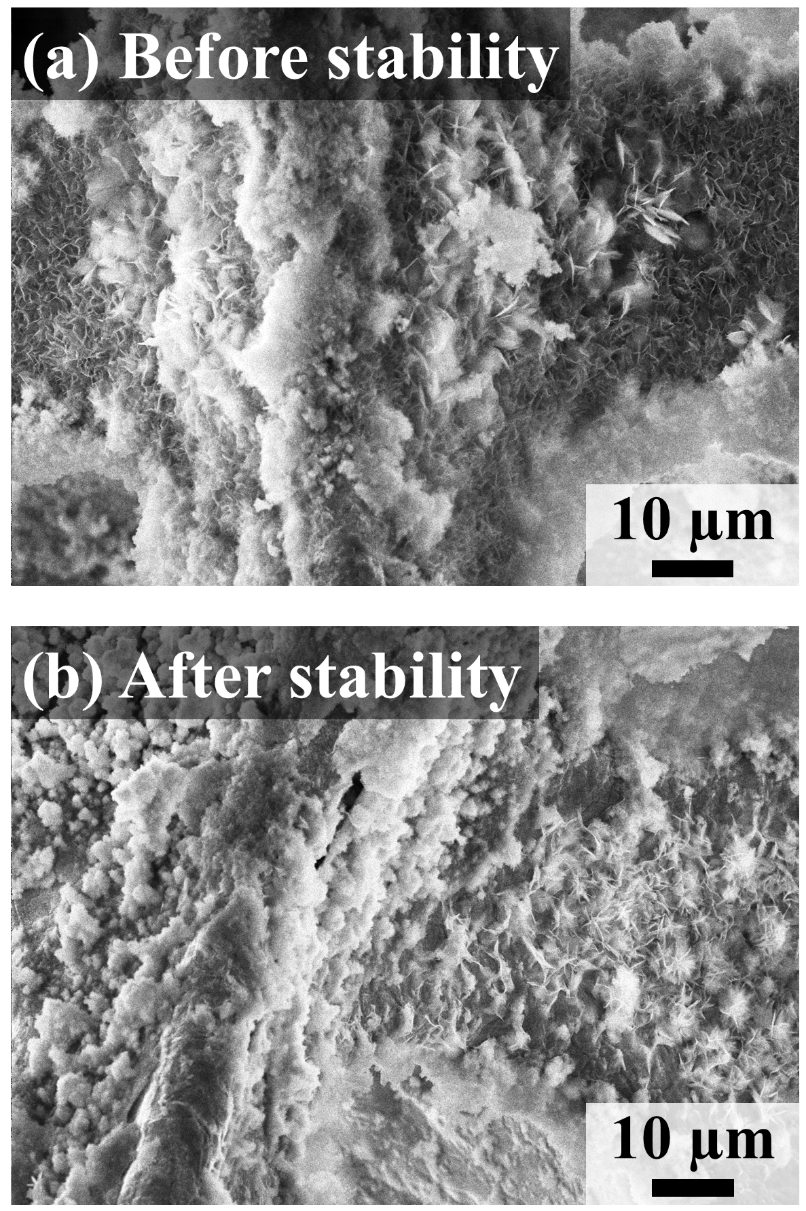
**

**Figure S58.** Post-stability analysis of Co/FeMoB electrodes: CP-stability test was conducted at a high current density of 1,000 mA/cm^2^ for 30 hours. (a–b) SEM micrographs of the Co/FeMoB electrodes (a) before and (b) after the stability operation.

**S-3.5.2. EDS spectra**

**
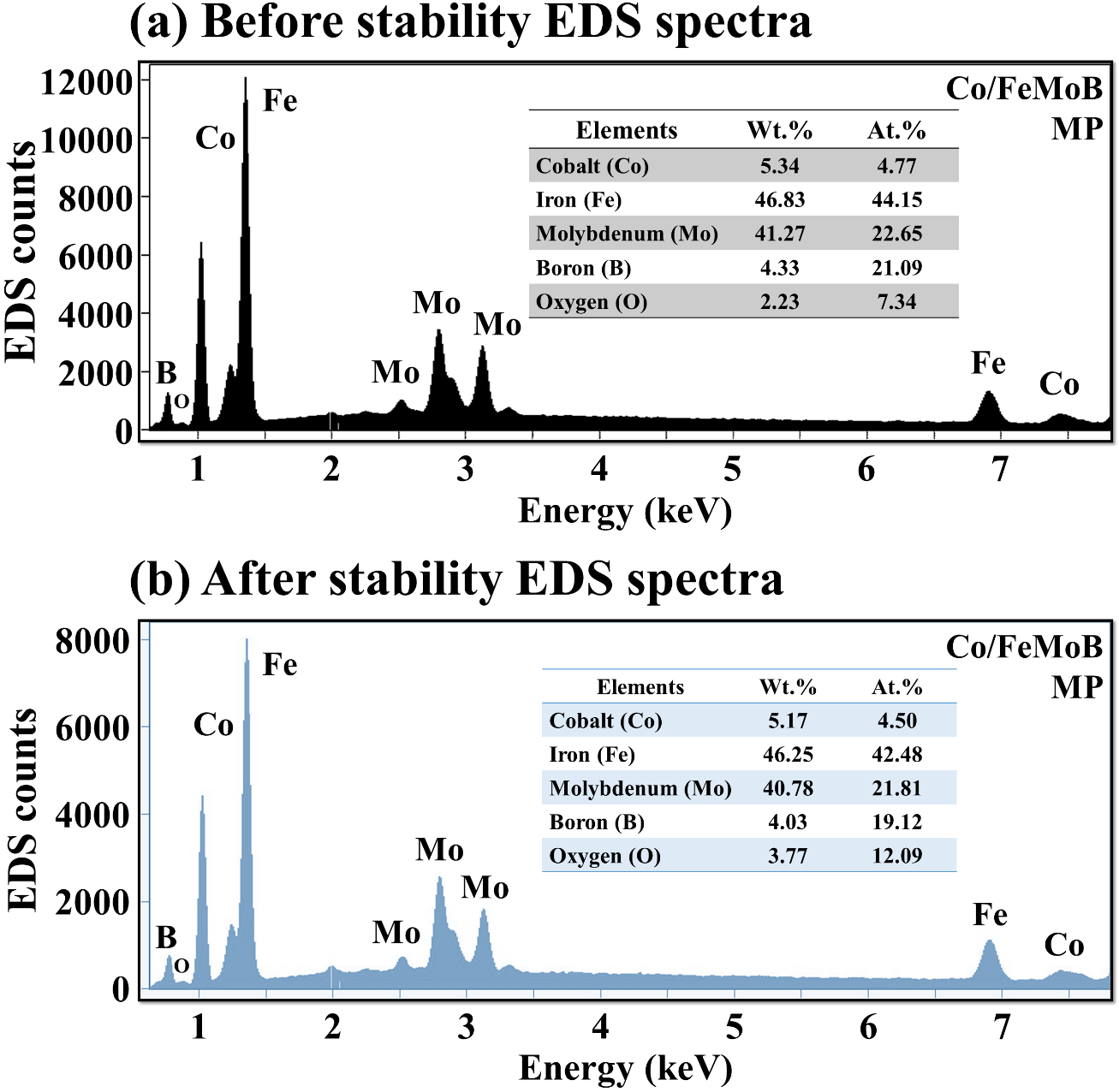
**

**Figure S59.** Post-stability analysis of Co/FeMoB electrodes: EDS spectra. (a) before and (b) after the stability operation.

**S-3.5.3. Raman spectra**

**
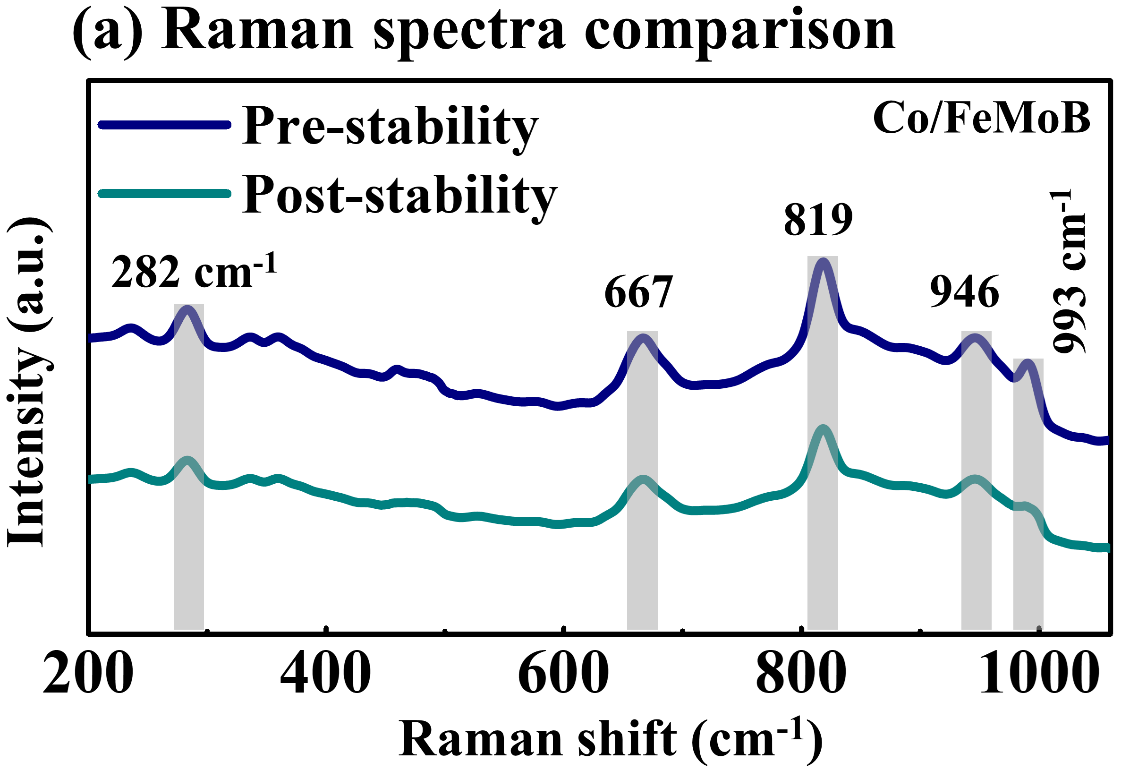
**

**Figure S60.** Raman spectrocopy of Co/FeMoB MP electrodes long-run and large-current stability oparation. (a) Before and after stability.

**S-3.5.4. XRD pattern**

**
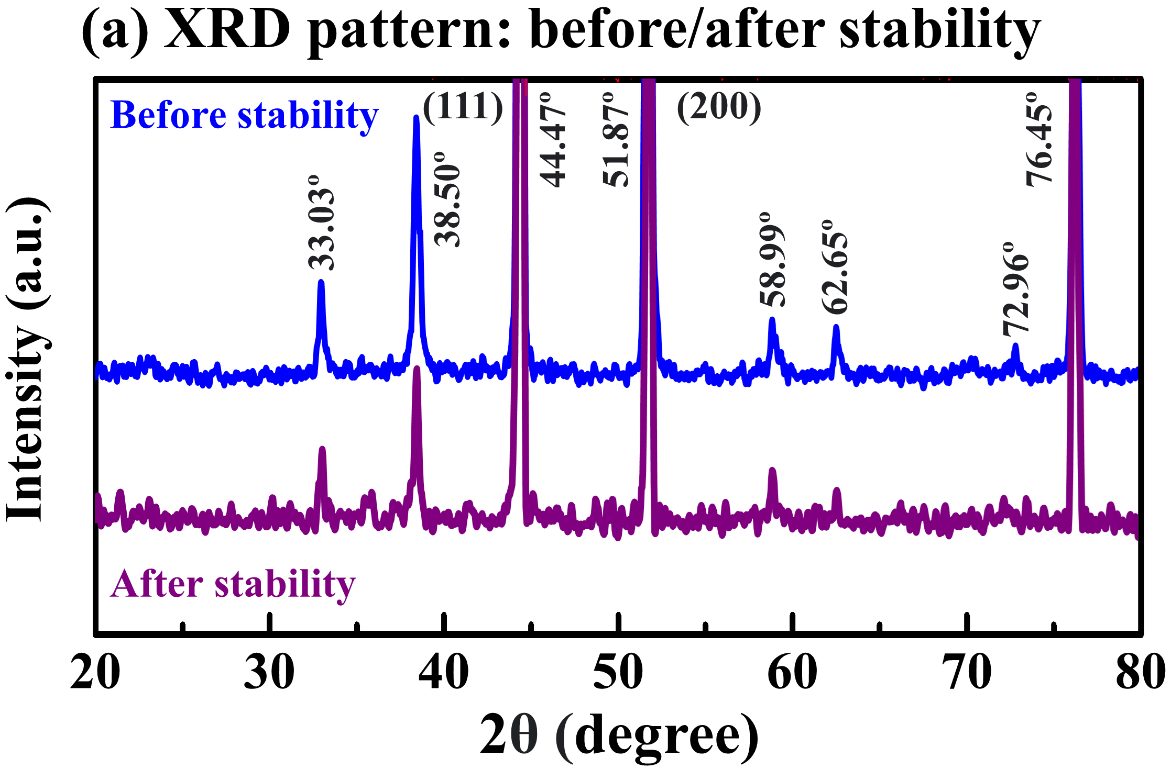
**

**Figure S61.** Full-range XRD diffraction pattern of Co/FeMoB electrodes before/after stability oparation. (a) Before stability. (b) After stability.

**S-3.5.5. Full-scan XPS**

**
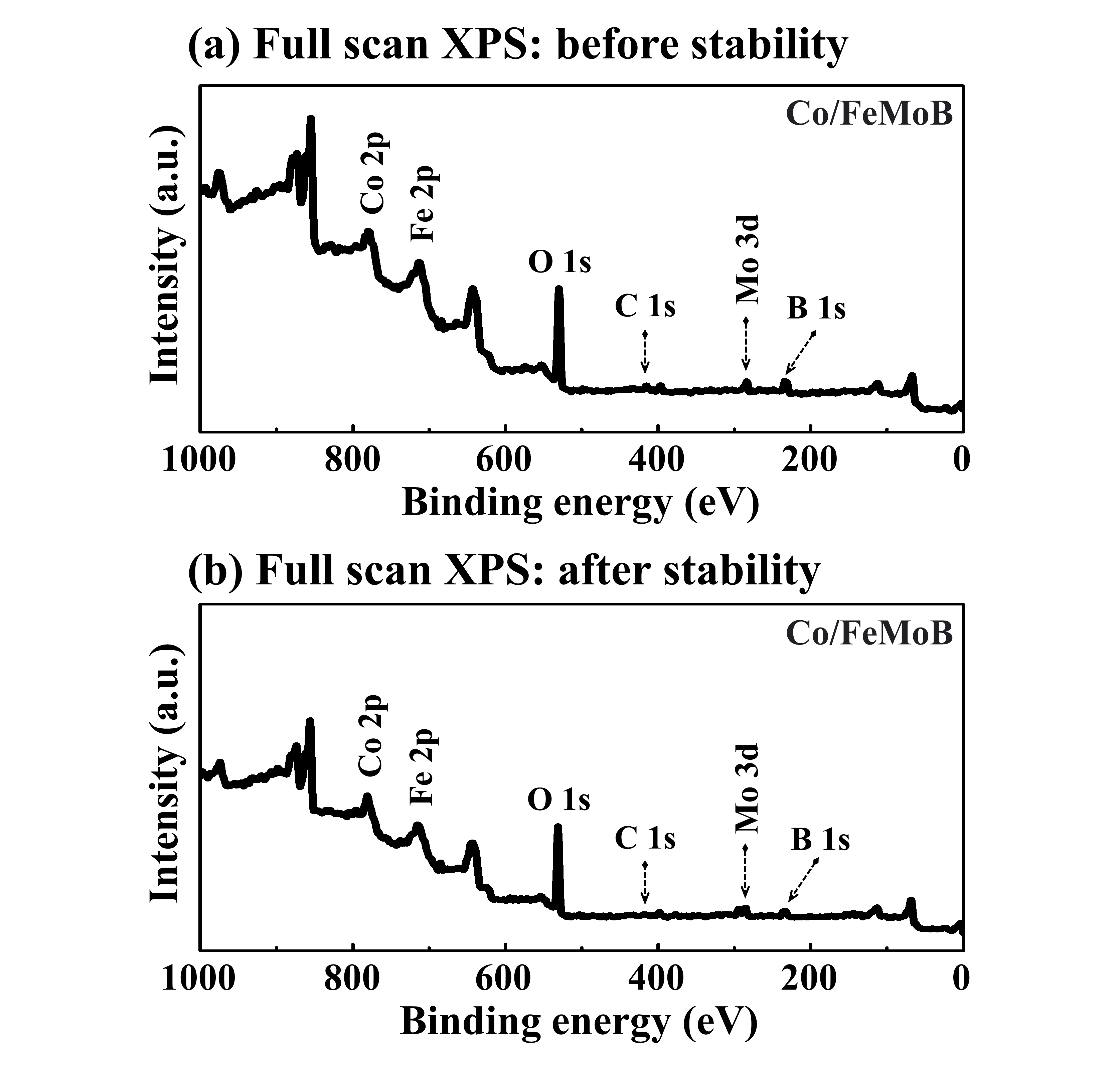
**

**Figure S62.** Full-scan XPS spectra of Co/FeMoB electrodes before/after stability oparation. (a) Before stability. (b) After stability.

**S-3.5.6. High-resolution XPS**

**
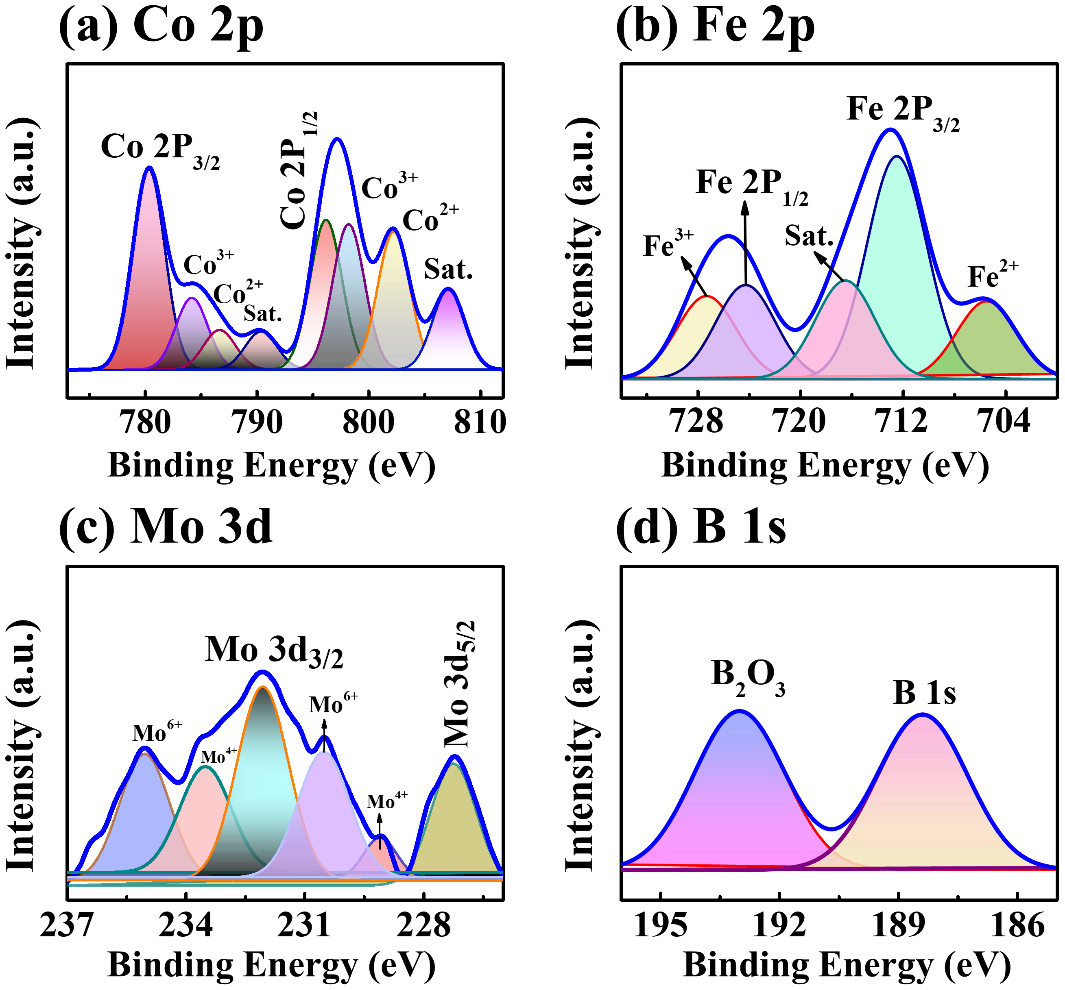
**

**Figure S63.** High-resolution XPS spectra of Co/FeMoB electrode after 20-hour stability test at 1,000 mA/cm^2^: (a–d) Core-level spectra of (a) Co 2p (b) Fe 2p (c) Mo 3d and (d) B 1s recorded after the long-term CP stability operation, providing insights into the chemical state and surface composition of the electrode.

**S-3.5.7. 3-E HER/OER and 2-E OWE**

**
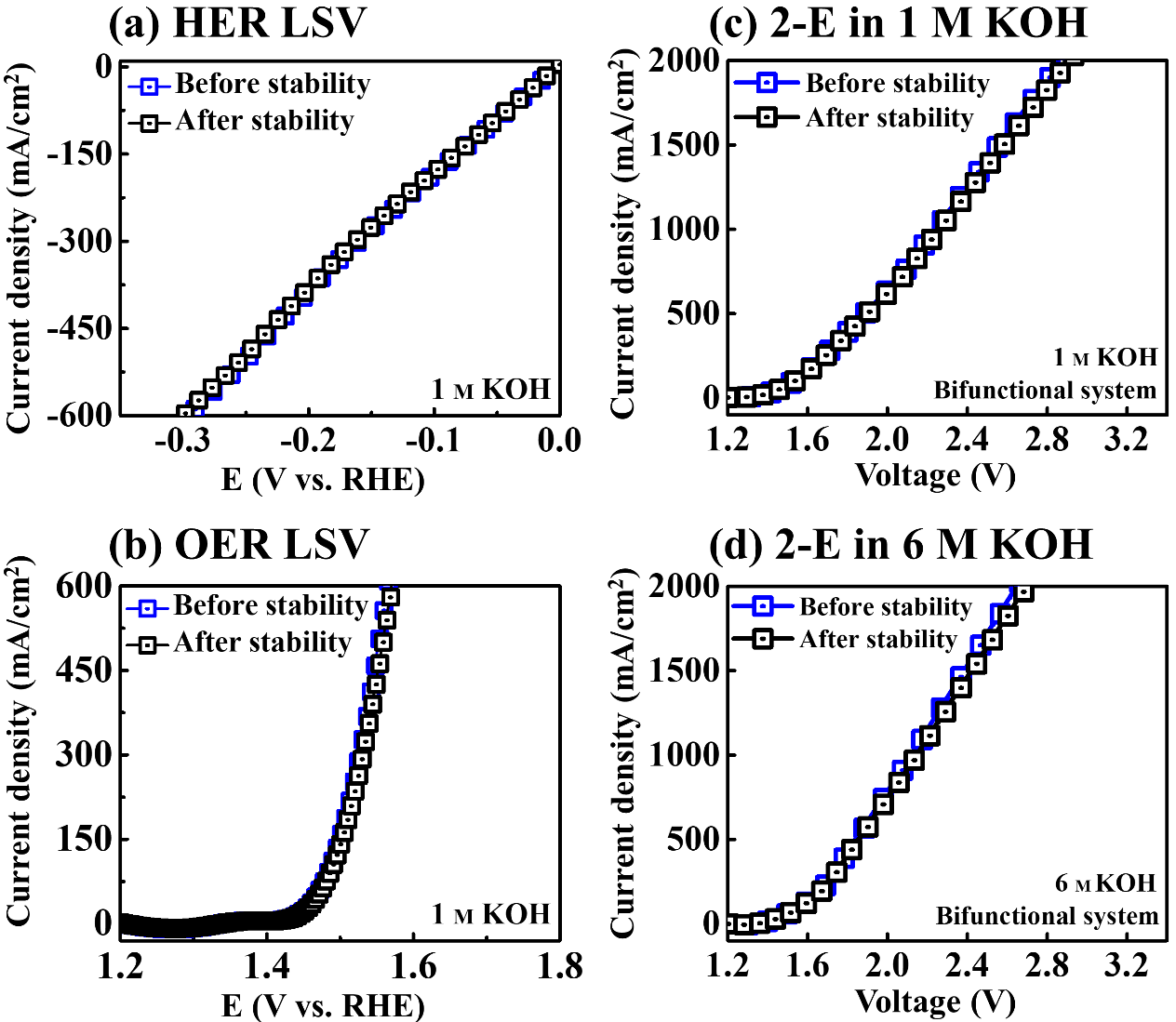
**

**Figure S64.** 3-E and 2-E electrocatalytic activity of Co/FeMoB electrode after 30-hour stability test: (a–b) LSV polarization curves for HER/OER in 1 m KOH. (c–d) 2-E high-current bifunctional electrochemical performance evaluated in both 1/6 m KOH demonstrates excellent catalytic activity with superior durability of the Co/FeMoB electrocatalyst under harsh conditions.

**Table S1.** 3-E HER/OER electrochemical performance of the best Co/FeMoB in 1 **m** KOH.

| **Co/FeMoB** | **HER activity** | **OER activity** |
| --- | --- | --- |
| **EIS values** | 28.25 Ω | 25.95 Ω |
| **Tafel slopes** | 53 mV/dec | 123 mV/dec |
| **TOF values** | 4.83 site^-1^-s^-1^ | 2.41 site^-1^-s^-1^ |
| **C_dl_ values** | 6.31 mF/cm^2^ | 2.48 mF/cm^2^ |
| **ECSA** | 63.82 cm^2^ | 44.75 cm^2^ |
| **Faradic efficiency** | 96.06 % | 95.34% |

**Table S2.** 3-E HER/OER activity of the Co/FeMoB electrode in different pH solutions at 600 mA/cm^2^ current density.

| **Different pH** | **HER**  **@ 600 mA/cm^2^** | **OER**  **@ 600 mA/cm^2^** |
| --- | --- | --- |
| **1 m KOH** | 295 mV | 334 mV |
| **0.5 m H_2_SO_4_** | 328 mV | 496 mV |
| **1 m PBS** | 369 mV | 566 mV |

**Table S3.** 2-E bifunctional activity of the Co/FeMoB electrode in different pH solutions at 100 and 500 mA/cm^2^.

| **Different pH** | **Potential (V)**  **@ 100 mA/cm^2^** | **Potential (V)**  **@ 600 mA/cm^2^** |
| --- | --- | --- |
| **1 m KOH** | 1.65 V | 1.96 V |
| **0.5 m H_2_SO_4_** | 1.69 V | 2.06 V |
| **1 m PBS** | 1.73 V | 2.15 V |

**Table S4.** 2-E summary of bifunctional Co/FeMoB and benchmarks at high-current density in 1 and 6 m KOH.

| **2-E performance** | **Electrolyte** | **2-E**  **@ 2,000 mA/cm^2^** |
| --- | --- | --- |
| **Co/FeMoB (-,+)** | 1 m KOH | 2.87 V |
| **Pt/C (-) \|\| RuO_2_ (+)** | 1 m KOH | 3.26 V |
| **Co/FeMoB (-,+)** | 6 m KOH | 2.65 V |

**Table S5.** 2-E hybrid system activity of the Co/FeMoB electrodes in different pH solutions.

| **Different pH** | **Potential (V)**  **@ 100 mA/cm^2^** | **Potential (V)**  **@ 600 mA/cm^2^** |
| --- | --- | --- |
| **1 m KOH** | 1.67 V | 1.86 V |
| **0.5 m H_2_SO_4_** | 1.71 V | 2.14 V |
| **1 m PBS** | 1.76 V | 2.28 V |

**Table S6.** 2-E summary of hybrid Co/FeMoB MCC and benchmarks at high-current density in 1 and 6 m KOH.

| **2-E performance** | **Electrolyte** | **2-E**  **@ 2,000 mA/cm^2^** |
| --- | --- | --- |
| **Pt/C (-) \|\| Co/FeMoB (+)** | 1 m KOH | 2.26 V |
| **Pt/C (-) \|\| Co/FeMoB (+)** | 6 m KOH | 2.37 V |
| **Pt/C (-) \|\| RuO_2_ (+)** | 1 m KOH | 3.14 V |

**Table S7.** 2-E catalysis summary of bifunctional and hybrid Co/FeMoB MCC and benchmarks in natural waters with/without KOH electrolytes.

| **2-E performance** | **Electrolyte** | **2-E**  **Cell voltage (V)** |
| --- | --- | --- |
| **Co/FeMoB (-,+)** | Fresh SW | 4.98 V, ɳ= 900 mA/cm^2^ |
| **Pt/C (-) \|\| RuO_2_ (+)** | Fresh SW | 7.16 V, ɳ=1,000 mA/cm^2^ |
| **Pt/C (-) \|\| Co/FeMoB (+)** | Fresh SW | 5.38 V, ɳ=1,000 mA/cm^2^ |
| **Co/FeMoB (-,+)** | SW + 1 m KOH | 3.40 V, ɳ=1,200 mA/cm^2^ |
| **Pt/C (-) \|\| RuO_2_ (+)** | SW + 1 m KOH | 5.15 V, ɳ=1,200 mA/cm^2^ |
| **Pt/C (-) \|\| Co/FeMoB (+)** | SW + 1 m KOH | 3.12 V, ɳ=1000 mA/cm^2^ |
| **Co/FeMoB (-,+)** | RW + 1 m KOH | 4.48 V, ɳ=600 mA/cm^2^ |
| **Pt/C \|\| RuO_2_ (+)** | RW + 1 m KOH | 5.05 V, ɳ=500 mA/cm^2^ |
| **Pt/C (-) \|\| Co/FeMoB (+)** | RW + 1 m KOH | 4.02 V, ɳ=600 mA/cm^2^ |

**Table S8.** Three electrode (3-E) HER comparisons recently reported electrocatalysts at 100, 500 and 600 mA/cm^2^ current densities in alkaline (1 m KOH) solution.

| Electrocatalysts | Overpotential  [mV]  100 mA/cm^2^ | Overpotential  [mV]  300 mA/cm^2^ | Overpotential  [mV]  600 mA/cm^2^ | Year | | References | | |
| --- | --- | --- | --- | --- | --- | --- | --- | --- |
| Co/FeMoB | **54** | **160** | **248** | | **-** | | **This work** |  |
| Ru-CoO_x_/NF | 78 | 127 | 196 | | 2021 | | ^[48]^ |  |
| MnO-CoP | 92 | 148 | 202 | | 2023 | | ^[49]^ |  |
| Cr/NiMoB SC | 102 | 195 | 278 | | 2025 | | ^[10]^ |  |
| (Ni-Fe)S_x_/(OH)_y_ | 132 | 170 | N/A | | 2019 | | ^[50]^ |  |
| FeWB//NF | 135 | 219 | N/A | | 2025 | | ^[51]^ |  |
| Ni-B-P/NF | | 139 | 265 | N/A | | 2023 | | ^[25]^ |
| (Fe, Ni)_2_P@Ni_2_P | | 150 | 197 | 235 | | 2023 | | ^[52]^ |
| Fe-Ni_2_P/CeO_2_ | | 180 | 152 | 202 | | 2024 | | ^[53]^ |
| Li-V/Ni_3_S_2_ | | 183 | 265 | 352 | | 2023 | | ^[54]^ |
| Pt_SA_/Se-DM-Co_1-x_ | | 185 | 157 | N/A | | 2025 | | ^[55]^ |
| NiFe_2_O_4_/NiMoO_4_ | | 198 | 275 | N/A | | 2025 | | ^[56]^ |
| Mo-NiFeP | 218 | 280 | N/A | | 2023 | | ^[57]^ |  |
| Ni_2_P-Fe_2_P | 226 | 273 | 310 | | 2021 | | ^[58]^ |  |
| Mo/FCL/MX | 250 | 320 | N/A | | 2025 | | ^[59]^ |  |

**Table S9.** Three electrode (3-E) OER comparisons recently reported electrocatalysts at 100, 500 and 600 mA/cm^2^ current densities in alkaline (1 m KOH) solution.

| Electrocatalysts | Overpotential  [mV]  100 mA/cm^2^ | Overpotential  [mV]  300 mA/cm^2^ | Overpotential  [mV]  600 mA/cm^2^ | Year | | References | | |
| --- | --- | --- | --- | --- | --- | --- | --- | --- |
| MnO-CoP | 162 | 365 | 382 | | 2023 | | ^[49]^ |  |
| Cr/NiMoB | | 200 | 386 | 495 | | 2025 | | ^[10]^ |
| Ni_2_P-Fe_2_P | | 226 | 297 | 318 | | 2021 | | ^[58]^ |
| Co/FeMoB | **257** | **298** | **334** | | **-** | | **This work** |  |
| Ru-Fe/Ni_2_P | 265 | 282 | 305 | | 2023 | | ^[60]^ |  |
| Fe/NiBP | **280** | **320** | **705** | | **2022** | | ^[61]^ |  |
| Mo-NiFeP | 286 | 328 | N/A | | 2023 | | ^[57]^ |  |
| Ni-B-P/NF | | 290 | 360 | N/A | | 2023 | | ^[25]^ |
| (Ni-Fe)S_x_/(OH)_y_ | | 292 | 358 | 420 | | 2019 | | ^[50]^ |
| FeP_4_/NiP_2_ | | 310 | 342 | N/A | | 2022 | | ^[62]^ |
| Cr-Co_x_P | | 315 | 358 | 382 | | 2023 | | ^[63]^ |
| Ru/NiMnB | | 320 | 395 | 450 | | 2024 | | ^[40]^ |
| NiCoS_x_Se_y_ | | 350 | 375 | 394 | | 2022 | | ^[64]^ |
| Ru-FeP_4_/IF | | 372 | 420 | 462 | | 2022 | | ^[65]^ |
| NiMoB/NF | | 380 | 432 | 605 | | 2022 | | ^[66]^ |

**Table S10.** Comparison of three-electrode (3-E) HER/OER performances of recently reported Co-doped transition metal (TM)-based electrocatalysts at 100 mA/cm² current density.

| Electrocatalysts | HER  Overpotential (mV) | OER  Overpotential (mV)_(Position)_ | Electrolyte | Year | | References | | |
| --- | --- | --- | --- | --- | --- | --- | --- | --- |
| Co/FeMoB MP | **54** | **257_(3)_** | **1** m **KOH** | | **-** | | **This work** |  |
| CoP-(N, S)-C | 75 | 308_(6)_ | 1 m KOH | | 2023 | | ^[67]^ |  |
| Co-MoS_2_-0.5 | 143 | 275_(4)_ | 1 m KOH | | 2018 | | ^[68]^ |  |
| Co_0.13_-Ni_0.87_Se_2_/Ti | 145 | 318_(7)_ | 1 m KOH | | 2016 | | ^[69]^ |  |
| Co-NiMoN-400 | 148 | 362_(11)_ | 1 m KOH | | 2019 | | ^[70]^ |  |
| Co-Ni_3_N/NF | 157 | 430_(14)_ | 1 m KOH | | 2021 | | ^[71]^ |  |
| Co-MoS_2_@CC | 170 | 320_(8)_ | 1 m KOH | | 2021 | | ^[72]^ |  |
| Co-Ni-Se/C/NF | 175 | 276_(5)_ | 1 m KOH | | 2016 | | ^[73]^ |  |
| Co-NiS@MoS_2_ | 201 | 207_(2)_ | 1 m KOH | | 2021 | | ^[74]^ |  |
| Co-Fe_3_O_4_/IF | 203 | 352_(10)_ | 1 m KOH | | 2025 | | ^[75]^ |  |
| Mo,Co-NiS/NF-400 | 210 (ɳ:60 mA) | 200_(1)_ | 1 m KOH | | 2019 | | ^[76]^ |  |
| Co-NixP@C | 215 | 333_(9)_ | 1 m KOH | | 2022 | | ^[77]^ |  |
| NHCo-MX-Ti_3_C_2_ | 237 | 425_(13)_ | 1 m KOH | | 2025 | | ^[78]^ |  |
| Co_0.03_-NiS_8.9_S_8_ | 290 | 405_(12)_ | 1 m KOH | | 2022 | | ^[79]^ |  |
| Co-Ni_3_S_2_(E.G) | 335 | 562_(17)_ | 1 m KOH | | 2025 | | ^[80]^ |  |
| Co-NiSe_2_/NF-0.1 | 422 | 460_(15)_ | 1 m KOH | | 2025 | | ^[81]^ |  |
| Co_0.08_-CuSe_0.02_O | 505 (ɳ:80 mA) | 558_(16)_ | 1 m KOH | | 2024 | | ^[82]^ |  |

**Table S11.** Two electrode (2-E) overall water electrolysis (OWE) comparisons recently reported electrocatalysts at 100, 500 and 1,000 mA/cm^2^ current densities in alkaline (1 m KOH) solution.

| Electrocatalysts | Voltage  [V]  100 mA/cm^2^ | Voltage  [V]  500 mA/cm^2^ | Voltage  [V]  1,000 mA/cm^2^ | Year | | References | | |
| --- | --- | --- | --- | --- | --- | --- | --- | --- |
| Ru&Fe-WO_3_ | 1.51 | 1.64 | N/A | | 2021 | | ^[83]^ |  |
| Bi. Co/FeMoB | **1.53** | **1.88** | **2.24** | | **-** | | **This work** |  |
| Hy. Co/FeMoB | | **1.58** | **1.81** | **2.00** | | **-** | | **This work** |
| MnO-CoP | 1.60 | 1.76 | N/A | | 2023 | | ^[49]^ |  |
| Cr/NiMoB SC | 1.63 | 1.93 | 2.24 | | 2025 | | ^[10]^ |  |
| (Fe, Ni)_2_P@Ni_2_P | 1.66 | 1.84 | 1.95 | | 2023 | | ^[52]^ |  |
| Ni_2_P-Fe_2_P | 1.68 | 1.86 | 1.96 | | 2021 | | ^[58]^ |  |
| Mo-NiFeP | 1.69 | 1.80 | 1.88 | | 2023 | | ^[57]^ |  |
| Ni_2_P/FeP | 1.70 | N/A | N/A | | 2023 | | ^[84]^ |  |
| Li-V/Ni_3_S_2_ | 1.71 | 1.80 | 1.94 | | 2023 | | ^[54]^ |  |
| (Ni-Fe)S_x_/(OH)_y_ | 1.73 | 2.54 | N/A | | 2019 | | ^[50]^ |  |
| NC-PB@CNT | 1.75 | N/A | N/A | | 2021 | | ^[85]^ |  |
| NiMoB/NF | 1.76 | 2.20 | 2.45 | | 2022 | | ^[66]^ |  |
| NiFe/Ni/Ni | 1.79 | 1.96 | N/A | | 2019 | | ^[86]^ |  |
| MoNiFe | 1.82 | 1.98 | N/A | | 2023 | | ^[46]^ |  |
| Fe_2_O_3_@Ni_2_P | 1.97 | 2.49 | 3.05 | | 2019 | | ^[56]^ |  |
| Fe-Ni_2_P/CeO_2_ | 2.05 | 2.31 | 2.88 | | 2024 | | ^[53]^ |  |

**Table S12.** Two electrode (2-E) overall water electrolysis (OWE) comparisons recently reported electrocatalysts at 100, 500 and 1,000 mA/cm^2^ current densities in alkaline seawater (SW + 1 m KOH) solution.

| Electrocatalysts | Voltage  [V]  100 mA/cm^2^ | Voltage  [V]  500 mA/cm^2^ | Voltage  [V]  1,000 mA/cm^2^ | Year | | References | | |
| --- | --- | --- | --- | --- | --- | --- | --- | --- |
| Ru/NiMnB | 1.58 | 2.42 | N/A | | 2024 | | ^[40]^ |  |
| Hy. Co/FeMoB | **1.66** | **2.19** | **2.83** | | **-** | | **This work** |  |
| Bifun. Co/FeMoB | **1.69** | **2.29** | **3.07** | | **-** | | **This work** |  |
| Ru-Ni_2_P/Fe_2_P | 1.72 | 1.91 | N/A | | 2024 | | ^[87]^ |  |
| Cr/NiMoB SC | 1.82 | 2.99 | 4.28 | | 2025 | | ^[10]^ |  |
| Mn-NiP/Fe_2_P | 1.83 | 2.08 | N/A | | 2022 | | ^[88]^ |  |
| FeWB/NF | 1.85 | 2.26 | 2.88 | | 2025 | | ^[51]^ |  |
| Se-FeCo-LDH | 1.87 | N/A | N/A | | 2023 | | ^[89]^ |  |
| Ru-Ni_2_P/Fe_2_P | 1.88 | 2.21 | N/A | | 2023 | | ^[90]^ |  |
| MnCo/NiSe | 1.88 | 2.04 | 2.03 | | 2024 | | ^[91]^ |  |
| Ni-BP/NF | 1.90 | 2.76 | 4.04 | | 2023 | | ^[25]^ |  |
| Co-N_3_-P_1_ HCS | 2.07 | N/A | N/A | | 2022 | | ^[92]^ |  |
| N-S/Cu8S5 | 2.10 | N/A | N/A | | 2023 | | ^[93]^ |  |
| Mo/CoPx | 2.17 | N/A | N/A | | 2022 | | ^[94]^ |  |
| Ir-Co_2_P/Co_2_P_2_O_7_ | 3.09 | N/A | N/A | | 2023 | | ^[95]^ |  |

**Reference:**

[1] J. Gautam, S.-Y. Lee, S.-J. Park, *Adv. Compos. Hybrid Mater.* **2024**, *7*, 155.

[2] M. Ahasan Habib, R. Mandavkar, S. Lin, S. Burse, T. Khalid, M. Hasan Joni, J. H. Jeong, J. Lee, *Chem. Eng. J.* **2023**, *462*, 142177.

[3] B. Zhang, C. Xiao, S. Xie, J. Liang, X. Chen, Y. Tang, *Chem. Mater.* **2016**, *28*, 6934.

[4] L. He, W. Zhang, Q. Mo, W. Huang, L. Yang, Q. Gao, *Angew. Chemie* **2020**, *132*, 3572.

[5] M. A. Habib, S. Lin, S. A. Dristy, M. H. Joni, R. Mandavkar, J. H. Jeong, J. Lee, *Small Methods* **2025**, *2401939*, 1.

[6] H. Jin, X. Liu, S. Chen, A. Vasileff, L. Li, Y. Jiao, L. Song, Y. Zheng, S.-Z. Qiao, *ACS Energy Lett.* **2019**, *4*, 805.

[7] X. F. Lu, L. Yu, X. W. Lou, *Sci. Adv.* **2019**, *5*, eaav6009.

[8] X. Xu, C. Su, W. Zhou, Y. Zhu, Y. Chen, Z. Shao, *Adv. Sci.* **2016**, *3*, 1500187.

[9] M. A. Habib, S. Burse, S. Lin, R. Mandavkar, M. H. Joni, J. H. Jeong, S. S. Lee, J. Lee, *Small* **2023**, *2307533*, 1.

[10] M. H. Joni, M. A. Habib, S. Lin, S. A. Dristy, R. Mandavkar, J. Lee, *J. Power Sources* **2025**, *630*, 236188.

[11] A. Cremona, C. Colombo, .

[12] Y. Lin, Y. Pan, J. Zhang, Y. Chen, K. Sun, Y. Liu, C. Liu, *Electrochim. Acta* **2016**, *222*, 246.

[13] H. Zhao, T. Xing, L. Li, X. Geng, K. Guo, C. Sun, W. Zhou, H. Yang, R. Song, B. An, *Int. J. Hydrogen Energy* **2019**, *44*, 25180.

[14] A. Manuscript, R. Society, A. Manuscripts, T. A. Manuscript, A. Manuscripts, R. Society, A. Manuscript, .

[15] S. Anantharaj, S. Noda, M. Driess, P. W. Menezes, *ACS Energy Lett.* **2021**, *6*, 1607.

[16] L. Yu, Z. Ren, *Mater. Today Phys.* **2020**, *14*, 100253.

[17] S. A. Dristy, M. A. Habib, M. H. Joni, M. Najibullah, R. Mandavkar, S. Lin, J. Lee, *Chinese J. Struct. Chem.* **2025**, 100747.

[18] Q. Zhou, G. Song, J. Zou, S. Luo, A. Meng, Z. Li, *Int. J. Hydrogen Energy* **2023**, *48*, 15921.

[19] Y. Huang, L. Jiang, B. Shi, K. M. Ryan, J. Wang, *Adv. Sci.* **2021**, *8*, 2101775.

[20] S. Lin, M. A. Habib, M. H. Joni, S. A. Dristy, R. Mandavkar, J.-H. Jeong, Y.-U. Chung, J. Lee, *CoFeBP Micro Flowers (MFs) for Highly Efficient Hydrogen Evolution Reaction and Oxygen Evolution Reaction Electrocatalysts*, Vol. 14, **2024**.

[21] S. Anantharaj, P. E. Karthik, S. Noda, *Angew. Chemie Int. Ed.* **2021**, *60*, 23051.

[22] R. A. Qureshi, A. Ali, M. Y. Solangi, M. A. Shar, A. Alhazaa, I. A. Soomro, M. A. Qureshi, M. Kumar, H. M. Ansari, A. Hanan, U. Aftab, *Int. J. Hydrogen Energy* **2025**, *155*, 150288.

[23] L. E. Briand, A. M. Hirt, I. E. Wachs, *J. Catal.* **2001**, *202*, 268.

[24] T. Kim, S. B. Roy, S. Moon, S. H. Yoo, H. Choi, V. G. Parale, Y. Kim, J. Lee, S. C. Jun, K. Kang, S. H. Chun, K. Kanamori, H. H. Park, *ACS Nano* **2022**, *16*, 1625.

[25] M. Ahasan Habib, R. Mandavkar, S. Lin, S. Burse, T. Khalid, M. Hasan Joni, J. H. Jeong, J. Lee, *Chem. Eng. J.* **2023**, *462*, 142177.

[26] X. Liu, H. Lu, S. Zhu, Z. Cui, Z. Li, S. Wu, W. Xu, Y. Liang, G. Long, H. Jiang, *Angew. Chemie Int. Ed.* **2023**, *62*, e202300800.

[27] X. Pan, Z. Zheng, X. Zhang, X. He, Y. An, Y. Hao, K. Huang, M. Lei, *Eng. Sci.* **2022**, *19*, 253.

[28] M. Liu, J.-A. Wang, W. Klysubun, G.-G. Wang, S. Sattayaporn, F. Li, Y.-W. Cai, F. Zhang, J. Yu, Y. Yang, *Nat. Commun.* **2021**, *12*, 5260.

[29] N. Danilovic, R. Subbaraman, D. Strmcnik, V. Stamenkovic, N. Markovic, *J. Serbian Chem. Soc.* **2013**, *78*.

[30] Y. Lin, Y. Dong, X. Wang, L. Chen, *Adv. Mater.* **2023**, *35*, 2210565.

[31] J. Chen, Y. Ma, C. Cheng, T. Huang, R. Luo, J. Xu, X. Wang, T. Jiang, H. Liu, S. Liu, *J. Am. Chem. Soc.* **2025**, *147*, 8720.

[32] D. Zheng, D. Xu, X. Lyu, T. Guo, D. Liu, *Colloids Surfaces A Physicochem. Eng. Asp.* **2025**, *708*, 135971.

[33] Y. Cao, J. Zeng, X. Zheng, Y. Liu, J. Lu, J. Zhang, Y. Wang, Y. Deng, W. Hu, *J. Mater. Sci. Technol.* **2025**, *227*, 67.

[34] H. Tüysüz, *Acc. Chem. Res.* **2024**, *57*, 558.

[35] R. Kothari, D. Buddhi, R. L. Sawhney, *Int. J. Hydrogen Energy* **2005**, *30*, 261.

[36] Y. Hu, Y. Li, B. Yao, G. Sun, F. Yu, C. Chi, C. Zhang, T. Liu, P. Zhang, H. Li, *ACS Catal.* **2025**, *15*, 13260.

[37] S. A. Dristy, S. Lin, M. A. Habib, M. H. Joni, R. Mandavkar, J. Lee, *Int. J. Hydrogen Energy* **2024**, *96*, 321.

[38] M. A. Habib, R. Mandavkar, S. Burse, S. Lin, R. Kulkarni, C. S. Patil, J.-H. Jeong, J. Lee, *Mater. Today Energy* **2022**, *26*, 101021.

[39] M. Kumar, T. C. Nagaiah, *J. Mater. Chem. A* **2023**, *11*, 18336.

[40] M. A. Habib, S. Lin, M. H. Joni, S. A. Dristy, R. Mandavkar, J.-H. Jeong, J. Lee, *J. Energy Chem.* **2025**, *100*, 397.

[41] M. Rafiq, K. Harrath, M. Feng, R. Li, A. R. Woldu, P. K. Chu, L. Hu, F. Lu, X. Yao, *Adv. Energy Mater.* **2024**, *14*, 2402866.

[42] Z. Sun, L. Lin, C. Nan, H. Li, G. Sun, X. Yang, *ACS Sustain. Chem. Eng.* **2018**, *6*, 14257.

[43] G. F. Chen, T. Y. Ma, Z. Q. Liu, N. Li, Y. Z. Su, K. Davey, S. Z. Qiao, *Adv. Funct. Mater.* **2016**, *26*, 3314.

[44] M. H. Joni, M. A. Habib, S. A. Dristy, M. Najibullah, S. Lin, R. Mandavkar, J. Lee, *Int. J. Hydrogen Energy* **2025**, *185*, 152003.

[45] Z. H. Zhang, Y. Zhang, A. Barras, A. Addad, P. Roussel, L. C. Tang, M. A. Amin, S. Szunerits, R. Boukherroub, *ACS Appl. Energy Mater.* **2022**, *5*, 15269.

[46] N. S. Gultom, T.-S. Chen, M. Z. Silitonga, D.-H. Kuo, *Appl. Catal. B Environ.* **2023**, *322*, 122103.

[47] Y. Zhang, L. Gao, E. J. M. Hensen, J. P. Hofmann, *ACS energy Lett.* **2018**, *3*, 1360.

[48] D. Wu, D. Chen, J. Zhu, S. Mu, *Small* **2021**, *17*, 2102777.

[49] Y. Dong, Z. Deng, H. Zhang, G. Liu, X. Wang, *Nano Lett.* **2023**, *23*, 9087.

[50] Q. Che, Q. Li, Y. Tan, X. Chen, X. Xu, Y. Chen, *Appl. Catal. B Environ.* **2019**, *246*, 337.

[51] M. H. Joni, S. Lin, M. A. Habib, S. A. Dristy, R. Mandavkar, J. Lee, *Nano Mater. Sci.* **2025**.

[52] Y. Li, X. Yu, J. Gao, Y. Ma, *Chem. Eng. J.* **2023**, *470*, 144373.

[53] Y. Huang, X. Ding, B. Huang, Z. Xie, *J. Alloys Compd.* **2024**, *981*, 173672.

[54] Q.-N. Ha, N. S. Gultom, C.-H. Yeh, D.-H. Kuo, *Chem. Eng. J.* **2023**, *472*, 144931.

[55] I. Pathak, D. Acharya, K. Chhetri, Y. R. Rosyara, T. Kim, T. H. Ko, H. Y. Kim, *ACS Appl. Mater. Interfaces* **2025**.

[56] X. Cheng, Z. Pan, C. Lei, Y. Jin, B. Yang, Z. Li, X. Zhang, L. Lei, C. Yuan, Y. Hou, *J. Mater. Chem. A* **2019**, *7*, 965.

[57] Y. Wang, P. Yang, Y. Gong, D. Liu, S. Liu, W. Xiao, Z. Xiao, Z. Li, Z. Wu, L. Wang, *Chem. Eng. J.* **2023**, *468*, 143833.

[58] L. Wu, L. Yu, F. Zhang, B. McElhenny, D. Luo, A. Karim, S. Chen, Z. Ren, *Adv. Funct. Mater.* **2021**, *31*.

[59] R. Liu, J. Yu, Z. Wang, Y. Liu, Y. Zhai, *J. Colloid Interface Sci.* **2025**, *699*, 138157.

[60] X. Zhai, Q. Yu, J. Chi, X. Wang, B. Li, B. Yang, Z. Li, J. Lai, L. Wang, *Nano Energy* **2023**, *105*, 108008.

[61] J. Zhang, L. L. Chen, B. Lu, Y. Guo, *ChemSusChem* **2022**, *15*.

[62] Y. Liu, F. Luo, X. Jiang, B. Yuan, S. Chen, *Int. J. Hydrogen Energy* **2022**.

[63] Y. Song, M. Sun, S. Zhang, X. Zhang, P. Yi, J. Liu, B. Huang, M. Huang, L. Zhang, *Adv. Funct. Mater.* **2023**, *33*, 2214081.

[64] S. Ma, J. Huang, C. Zhang, G. Chen, W. Chen, T. Shao, T. Li, X. Zhang, T. Gong, K. K. Ostrikov, *Chem. Eng. J.* **2022**, *435*, 134859.

[65] T. Cui, J. Chi, J. Zhu, X. Sun, J. Lai, Z. Li, L. Wang, *Appl. Catal. B Environ.* **2022**, *319*, 121950.

[66] R. Mandavkar, M. A. Habib, S. Lin, R. Kulkarni, S. Burse, J. H. Jeong, J. Lee, *Appl. Mater. Today* **2022**, *29*, 101579.

[67] M. R. Kandel, U. N. Pan, P. P. Dhakal, R. B. Ghising, S. Sidra, D. H. Kim, N. H. Kim, J. H. Lee, *Small* **2024**, *20*, 2307241.

[68] Q. Xiong, X. Zhang, H. Wang, G. Liu, G. Wang, H. Zhang, H. Zhao, *Chem. Commun.* **2018**, *54*, 3859.

[69] T. Liu, A. M. Asiri, X. Sun, *Nanoscale* **2016**, *8*, 3911.

[70] Z. Yin, Y. Sun, Y. Jiang, F. Yan, C. Zhu, Y. Chen, *ACS Appl. Mater. Interfaces* **2019**, *11*, 27751.

[71] M. Wang, W. Ma, Z. Lv, D. Liu, K. Jian, J. Dang, *J. Phys. Chem. Lett.* **2021**, *12*, 1581.

[72] Q. Wei, D. Wang, L. Zhang, L. Zhao, B. Zhang, G. Zhou, Y. Zhao, *Ceram. Int.* **2021**, *47*, 24501.

[73] F. Ming, H. Liang, H. Shi, X. Xu, G. Mei, Z. Wang, *J. Mater. Chem. A* **2016**, *4*, 15148.

[74] H. Gao, J. Zang, Y. Wang, S. Zhou, P. Tian, S. Song, X. Tian, W. Li, *Electrochim. Acta* **2021**, *377*, 138051.

[75] R. Liao, Z. Peng, X. Yang, J. Liu, J. Zhou, L. Yu, J. Liao, *Int. J. Hydrogen Energy* **2025**, *116*, 32.

[76] C. Wu, Y. Du, Y. Fu, D. Feng, H. Li, Z. Xiao, Y. Liu, Y. Yang, L. Wang, *Sustain. Energy Fuels* **2020**, *4*, 1654.

[77] M. Xing, D. Zhang, D. Liu, C. Song, D. Wang, *J. Colloid Interface Sci.* **2023**, *629*, 451.

[78] A. R. Manchuri, K. C. Devarayapalli, B. Kim, Y. Lim, D. S. Lee, *Green Energy Environ.* **2025**, *10*, 854.

[79] D. Rathore, S. Ghosh, J. Chowdhury, S. Pande, *ACS Appl. Nano Mater.* **2022**, *5*, 11823.

[80] S. M. Mane, K. S. Wagh, S. Lee, A. M. Teli, G. T. Chavan, J. C. Shin, J. Lee, *Solvent-Driven Structural Modulation of Co-Ni3S2 and Impact on Electrochemical Water Splitting*, Vol. 13, **2025**.

[81] Y. Wu, H. He, H. Jin, X. Zhou, Q. Wang, *Colloids Surfaces A Physicochem. Eng. Asp.* **2026**, *728*, 138644.

[82] U. Rashid, Y. Zhu, C. Cao, *J. Electroanal. Chem.* **2024**, *962*, 118267.

[83] Q. Yang, C. Zhang, B. Dong, Y. Cui, F. Wang, J. Cai, P. Jin, L. Feng, *Appl. Catal. B Environ.* **2021**, *296*, 120359.

[84] M. Jiang, H. Zhai, L. Chen, L. Mei, P. Tan, K. Yang, J. Pan, *Adv. Funct. Mater.* **2023**, *33*, 2302621.

[85] S. A. Patil, S. Cho, Y. Jo, N. K. Shrestha, H. Kim, H. Im, *Chem. Eng. J.* **2021**, *426*, 130773.

[86] P. can Wang, L. Wan, Y. qun Lin, B. guo Wang, *ChemSusChem* **2019**, *12*, 4038.

[87] X. Li, T. Wu, N. Li, S. Zhang, W. Chang, J. Chi, X. Liu, L. Wang, *Adv. Funct. Mater.* **2024**, *34*, 2400734.

[88] Y. Luo, P. Wang, G. Zhang, S. Wu, Z. Chen, H. Ranganathan, S. Sun, Z. Shi, *Chem. Eng. J.* **2023**, *454*, 140061.

[89] Y. Gong, H. Zhao, Y. Sun, D. Xu, D. Ye, Y. Tang, T. He, J. Zhang, *J. Colloid Interface Sci.* **2023**, *650*, 636.

[90] D. Wu, B. Liu, R. Li, D. Chen, W. Zeng, H. Zhao, Y. Yao, R. Qin, J. Yu, L. Chen, *Small* **2023**, *19*, 2300030.

[91] R. Andaveh, A. Sabour Rouhaghdam, J. Ai, M. Maleki, K. Wang, A. Seif, G. Barati Darband, J. Li, *Appl. Catal. B Environ.* **2023**, *325*, 122355.

[92] X. Wang, X. Zhou, C. Li, H. Yao, C. Zhang, J. Zhou, R. Xu, L. Chu, H. Wang, M. Gu, *Adv. Mater.* **2022**, *34*, 2204021.

[93] Y. Zhang, L. Chen, B. Yan, F. Zhang, Y. Shi, X. Guo, *Chem. Eng. J.* **2023**, *451*, 138497.

[94] Y. Yu, J. Li, J. Luo, Z. Kang, C. Jia, Z. Liu, W. Huang, Q. Chen, P. Deng, Y. Shen, *Mater. Today Nano* **2022**, *18*, 100216.

[95] V. H. Hoa, M. Austeria, H. Thi Dao, M. Mai, D. H. Kim, *Appl. Catal. B Environ.* **2023**, *327*, 122467.
